# Untargeted and Targeted Liquid Chromatography‐Mass Spectrometry–Based Lipidomic Profiling Revealed a Potential Biomarker Panel to Distinguish Clinical Isolates of *M. tuberculosis* From Nontuberculous *M. kansasii*

**DOI:** 10.1155/pm/5535650

**Published:** 2026-05-31

**Authors:** Meenakshi Chugh, Saif Hameed, Jitendra Singh, Zeeshan Fatima

**Affiliations:** ^1^ Amity Institute of Biotechnology, Amity University Haryana, Manesar, Gurugram, India, amity.edu; ^2^ Amity Medical School, Amity University Haryana, Manesar, Gurugram, India, amity.edu; ^3^ Department of Translational Medicine, All India Institute of Medical Sciences, Bhopal, India, aiims.edu

**Keywords:** biomarker panel, lipidomics, mass spectrometry, *Mycobacterium tuberculosis*, nontuberculous mycobacteria, tuberculosis

## Abstract

The rising incidence of nontuberculous mycobacteria (NTM) infections, particularly *Mycobacterium kansasii* (*M. kansasii*), and their overlapping clinical features with *Mycobacterium tuberculosis* (MTB) are leading to diagnostic ambiguity in tuberculosis‐endemic regions. Accurate differentiation remains limited by conventional diagnostic methods, emphasizing the need for molecular‐ and lipid‐based biomarkers. In the present era of “OMICS” sciences, herein we attempted a comprehensive analysis of MTB and *M. kansasii* lipid profiles to elucidate species‐specific lipidomic features as potential biomarkers. Three clinical isolates each of MTB and *M. kansasii* were obtained from treatment‐naïve patients with microbiologically confirmed pulmonary infections. Lipid profiling was performed by integrating untargeted and targeted profiling through ultraperformance liquid chromatography coupled with tandem mass spectrometry from bacilli total lipid and mycobacterial cell wall lipid extracts. Untargeted profiling exhibited a higher abundance of fatty acyls, GLs, selected GPLs (PE, PG, PI, and PA), and saccharolipids (SLs) specifically Ac2SGL in MTB, whereas *M. kansasii* isolates were characterized by elevated levels of GPLs like PC and LPC, polyketides, and specific SLs such as diacylated trehalose species. Targeted quantification confirmed differential expression of GL (TG and DG) and GPL (PC, PE, LPC, PI, and PS) species, supporting their diagnostic relevance. Additionally, biomarker analysis further identified five lipid species with strong discriminative potential. Collectively, these findings support the development of a robust lipidomic biomarker panel for the accurate differentiation of MTB and *M. kansasii*, with potential implications for improved diagnostics and targeted therapeutic strategies after further confirmation.

## 1. Introduction

Tuberculosis (TB), mainly caused by *Mycobacterium tuberculosis* (MTB), remains one of the deadliest communicable diseases and is among the top 10 causes of death worldwide by 2023 [1. Nontuberculous Mycobacteria (NTM) are a heterogeneous group comprising more than 169 species of environmental organisms found in soil, water, dust, and diverse animals, which can lead to opportunistic infections. The clinical presentation of NTM infections often overlaps with that of MTB, complicating diagnosis and treatment strategies. A further complex attribute of NTM species from MTB is evidenced by the fact that several presumed TB cases have been shown to be due to NTM [2, 3]. Among clinically relevant NTM, *Mycobacterium kansasii* (*M. kansasii*) is notable for its high pathogenic potential and TB‐like pulmonary manifestations [4]. Current diagnostic approaches, which primarily rely on Ziehl–Neelsen (ZN) staining and acid‐fast bacilli (AFB) culture, lack the specificity to reliably differentiate between MTB and NTM, thereby contributing to the underdiagnosis and underreporting of NTM infections in TB‐endemic regions. This diagnostic ambiguity underscores the urgent need for reliable molecular or biochemical markers capable of distinguishing between these clinically similar but biologically distinct mycobacterial groups.

A defining feature of the mycobacterial genus is its complex, lipid‐rich cell envelope, which accounts for up to 60% of its dry mass. This envelope comprises various structurally diverse lipid components including mycolic acids (MAs), sulfolipids, phosphatidylinositol (PI), phosphatidylinositol mannosides (PIMs), lipomannan (LM), lipoarabinomannan (LAM), and trehalose‐containing glycolipids, all of which contribute to cell wall integrity, virulence, and immune modulation [5, 6]. Although MTB and NTM share several of these lipid components, they also exhibit species‐specific lipid signatures. For instance, sulfoglycolipids (SL‐I) and polyacyltrehaloses (PATs) are restricted to the MTB complex and play key roles in immune evasion and intracellular survival. Conversely, *M. kansasii* and other NTM species uniquely express glycopeptidolipids, such as C‐mycosides, which are linked to biofilm formation and modulation of host immune responses [7, 8].

Previous lipidomic studies, including those by Layre et al. [9, 10], Sartain et al. [8], and Gonzalo et al. [7, 11], have provided valuable insights into mycobacterial lipid diversity but were largely limited in scope and depth. Layre et al. [9, 10] established a chemotaxonomic classification framework for differentiating *Mycobacterium tuberculosis* complex (MTBC) members based on lipid composition and identifying several virulence‐associated metabolites. However, their analyses were qualitative and restricted to MTBC species. Sartain et al. [8] developed the Mtb LipidDB for *M. tuberculosis* H37Rv using qualitative lipidomics, yet this work focused on a single strain without interspecies comparison and quantitative validation. In the present study, we utilized this database through MS‐LAMP software for accurate annotation and verification of MTB‐specific lipid masses from our IDA‐based acquired lipidomic data. Gonzalo et al. [7] analyzed clinical MTBC isolates and selected NTM species (*M. abscessus*, *M. avium*, *M. bohemicum*, *M. celatum*, *M. fortuitum*, *M. interjectum*, *M. intracellulare*, *M. kansasii*, *M. marinum*, *M. mucogenicum*, *M. nonchromogenicum*, *M. peregrinum*, *M. phlei*, *M. shimoidei*, *M. simiae*, *M. smegmatis*, *and M. xenopi*). They demonstrated lipid fingerprint patterns for rapid diagnostic differentiation based on dominant lipid masses. Their subsequent study [10] further refined MTBC identification but excluded NTMs altogether. Although their work established rapid diagnostic differentiation, it was based on a few dominant mass features rather than the entire lipidome and lacked both quantitative assessment and mechanistic interpretation of lipid variation.

More recently, Wang et al. [12] conducted a serum‐based lipidomic and proteomic comparison of patients infected with MTB and NTM, demonstrating differential host lipid responses. Although the lipid composition of MTB has been well studied, the lipid biology of NTM species, particularly *M. kansasii*, remains underexplored [5]. Furthermore, direct comparative analyses integrating untargeted and targeted LC‐MS–based lipidomics between MTB and NTM species such as *M. kansasii* are scarce. To date, systematic integration of untargeted and targeted LC‐MS/MS lipidomics encompassing both total and cell wall lipid (CWL) fractions with MTB and *M. kansasii* has not been reported.

Given the structural and functional diversity of mycobacterial lipids, major lipid classes viz. fatty acyl (FA), glycerolipid (GL), glycerophospholipid (GPL), polyketide (PK), prenol lipid (PR), and saccharolipid (SL) were analyzed to capture the full spectrum of metabolic and physiological adaptations, distinguishing MTB and *M. kansasii*. Profiling these lipid classes through both untargeted and targeted LC‐MS/MS enabled a comprehensive evaluation of lipid‐based metabolic signatures, ensuring that observed differences reflect true biological variation rather than analytical bias.

Addressing this gap, the present study integrates high‐resolution untargeted and targeted LC–MS/MS lipidomics to achieve comprehensive qualitative and quantitative profiling of clinical isolates of MTB and *M. kansasii*. The objective was to elucidate distinct lipidomic signatures, identify differentially expressed lipid species, and explore their potential as biomarkers for species discrimination and diagnostic development. The findings advance current understanding of mycobacterial lipid biology, offering mechanistic insight into lipid‐mediated pathogenesis and providing a foundation for developing lipid‐based diagnostic and chemotaxonomic tools.

## 2. Methodology

### 2.1. Material and Chemicals

Middlebrook 7H9 broth (BD Biosciences, United States), oleic acid/albumin/dextrose/catalase (OADC) (BD Biosciences, United States), Tween‐80 (Sigma‐Aldrich, United States), glycerol (Fisher Scientific), potassium chloride (KCl) (Fisher Scientific), chloroform (CHCl_3_) (Fisher Scientific and CHROMASOLV, Honeywell), methanol (CH_3_OH) (Fisher Scientific and CHROMASOLV, Honeywell), n‐hexane (C_6_H_14_) (Fisher Scientific), acetonitrile (C_2_H_3_N) (CHROMASOLV, Honeywell), 2‐propanol (C_3_H_8_O) (CHROMASOLV, Honeywell), ammonium acetate (Honeywell, Fluka), Teflon capping glass vials (Borosil), Pasteur pipette, and Whatman No. 1 filter paper (Merck). SPLASH LIPIDOMIX Mass Spec Standard and natural standards viz. 1,2‐dimyristoyl‐sn‐glycerol (DAG), 1,2‐diheptadecanoyl‐sn‐glycero‐3‐phosphocholine (PC), 1‐heptadecanoyl‐2‐hydroxy‐sn‐glycero‐3 phosphocholine (LPC), 1,2‐dimyristoyl‐sn‐glycero‐3 phosphoethanolamine (PE), 1‐heptadecanoyl‐2‐(9Z‐tetradecenoyl)‐sn glycero‐3‐phospho‐(1 ^′^‐myo‐inositol (PI), and 1,2‐dimyristoyl‐sn‐glycero‐3‐phospho‐L serine (PS) are from Avanti Polor, United State; Tripentadecanoin (TAG) natural standard from Sigma‐Aldrich, United States.

### 2.2. Clinical Sample Processing and Culture Conditions

Sputum samples were aseptically collected from patients with confirmed mycobacterial infections who had not yet started antitubercular or antibiotic therapy/treatment‐naive patients with microbiologically confirmed pulmonary mycobacterial infections. Diagnosis was established by ZN staining, AFB culture, and GeneXpert positivity. Mycobacteria were cultured from these samples under BSL‐3 conditions and considered as clinical isolates. Three independent clinical isolates each of MTB and *M. kansasii* were taken for this study.

Cultures were inoculated into a 30‐mL Middlebrook 7H9 medium supplemented with 0.05% Tween‐80, 0.2% glycerol, and 10% OADC enrichment and incubated at 37°C with agitation until the exponential phase (1 OD_600_ cells) was reached. Bacterial cells were carefully harvested and stored at −20°C for further processing.

### 2.3. Extraction of Total Lipids (TL)

The modified Folch method was used for TL extraction of *Mycobacterium* cells [13]. Cells were homogenized by sonication in an aqueous solution for 3 min, with three cycles of 1‐min intervals, and mixed with CHCl_3_ and CH_3_OH in a 1:2 ratio. A total of 10 *μ*L of Splash mix standard was added in each sample, and the solution was centrifuged at 2000 rpm at 4°C for 10–15 min. The supernatant was transferred to another glass vial, and the remaining CHCl_3_ was added to obtain a final volume of CHCl_3_ and CH_3_OH in a ratio of 1:1. After filtration with Whatman No.1 filter paper, nonlipid contamination was removed by saturating the extract with 0.88% KCl. The lower layer was taken using a Pasteur pipette in a 5‐mL glass vial with Teflon capping and dried with liquid nitrogen, then stored at −20°C for further analysis.

### 2.4. Extraction of CWL

CWL were extracted using the modified Minnikin method, as described previously [14]. The harvested cells were mixed with 3 mL of hexane and kept overnight in the dark at room temperature. The next day, the suspension was centrifuged at 2500 rpm for 10 min. The upper layer was collected in a glass vial and 10 *μ*L of Splash mix standard was added in each sample. After that, 3 mL of chloroform and after 30 min, 3 mL of methanol was added and kept at room temperature for 2 h. The upper hexane layer containing CWL was transferred to the glass vial and dried under gentle liquid nitrogen flux and then stored at −20°C for further use.

### 2.5. Electrospray Ionization Mass Spectrometry (ESI‐MS/MS)

The biological replicates were obtained from three TB patients, and each isolate has been analyzed in three independent technical replicates to ensure analytical reproducibility and precision in ESI‐MS/MS measurements.

#### 2.5.1. Untargeted Mass Spectrometry

For information‐dependent acquisition (IDA), TL and CWL samples were reconstituted in CHCl_3_ and CH_3_OH in 1:1 and 9:1 ratios, respectively, and analyzed by UHPLC‐MS/MS (ultrahigh–performance triple quadruple tandem mass spectrometer), that is, AB Sciex 4500 QTRAP MS/MS equipped with a Sciex Turbo Spray ion source, in both positive and negative modes. UHPLC was done on the Agilent C18 column (100 × 2.1 mm, 1.8 *μ*m) through gradient mode of elution for 41 min using Buffer A comprised of two solutions (Solution 1: water/acetonitrile (ACN) 95/5, v/v was prepared by mixing 5 mM of ammonium acetate; Solution 2, Solution 1/methanol, 90/10 v/v); and buffer B ACN/methanol/IPA 85/10/5, v/v/v at a flow rate of 0.3 mL/min. The source temperature was 500°C, the ion‐spray voltage (IV) was set to 5.50 kV, ion‐source Gasses 1 and 2 were set to 50, curtain gas was 30, and collision gas was set on the medium. 2 *μ*L of lipid sample was injected for each run, and the data were recorded in the 200–2000‐m/z mass range. Further, the analysis was done by using the mass spectrometry‐based lipid(ome) analyzer and molecular platform (MS‐LAMP) software [13, 15].

#### 2.5.2. Targeted Mass Spectrometry

##### 2.5.2.1. Optimization of ESI MS/MS Conditions for Absolute Quantification of GL and GPL

For optimization, direct infusion of internal and natural standards has been done by the Harvard syringe pump in conjunction with the UHPLC‐MS/MS. Internal standard SPLASH and natural standards viz. TAG (Tripentadecanoin), DAG (1,2‐dimyristoyl‐sn‐glycerol), PC (1,2‐diheptadecanoyl‐sn‐glycero‐3‐phosphocholine), LPC (1‐heptadecanoyl‐2‐hydroxy‐sn‐glycero‐3 phosphocholine), PE (1,2‐dimyristoyl‐sn‐glycero‐3 phosphoethanolamine), PI (1‐heptadecanoyl‐2‐(9Z‐tetradecenoyl)‐sn glycero‐3‐phospho‐(1 ^′^‐myo‐inositol)), and PS (1,2‐dimyristoyl‐sn‐glycero‐3‐phospho‐L serine) have been optimized. The flow rate for the syringe pump was maintained between 20 and 30 *μ*L/min, and the MS was configured to scan Q1 in the m/z range of 100–900 Da to obtain the mass spectrum of all the standards in positive ion modes.

The UHPLC gradient optimization was done on kinetex C18 column 100Ǻ (50 × 2.1 mm, 1.7 *μ*m) with gradient mode of elution. To separate targeted transition pairs, Buffer A comprises water with 0.5% formic acid and 10 mM of ammonium acetate, whereas Buffer B has methanol with 0.5% formic acid and 10 mM of ammonium acetate with a 0.3‐mL/min flow rate employed. A 30‐min run was performed for GLs (TG and DG), and 20 min for GPLs (PC, Lyso‐PC (LPC), PE, PI and PS) were applied. A total of 196 lipid species (125 for GL and 71 for GPL) were targeted for quantitative analysis. Oven and autosampler temperatures were adjusted to 60°C and 10°C, respectively. The source temperature was 500°C, the IS was set to 4.50 kV, ion‐source Gasses 1 and 2 were set to 50, curtain gas was 35, and collision gas was put on the medium. A total of 10 *μ*L of each lipid sample was injected by using an autosampler. Data were acquired in positive polarity in the 200–1000‐Da mass range. The UHPLC‐MS/MS was done in the multiple reaction monitoring (MRM) mode, with the detection of the transition pairs of the individual protonated parent ions and their common daughter ions. Software Analyst v1.6.3 was used for data acquisition in wiff format.

##### 2.5.2.2. Quality Control Measurements

Natural standards of DG, TG, PC, LPC, PE, PI, and PS, along with a pooled sample matrix, were prepared and analysed for quality control. The individual standards and mycobacterial test samples exhibited well‐resolved and distinct peaks, enabling absolute quantification of the targeted lipid species, including GL and GPL. Furthermore, the limit of detection (LOD) and limit of quantification (LOQ) were determined to validate the performance of the developed UHPLC‐MS/MS method. To assess the methodological LOD and LOQ, internal and natural standards were serially diluted within the range of 0.98–1000 ng/mL, and regression analysis was performed using Microsoft Excel.

### 2.6. High‐Throughput Data Analysis

#### 2.6.1. Untargeted Data Analysis

The MS‐LAMP software were used for interpreting IDA lipid data. In the current version of the program, two databases are incorporated based on lipid databases: “M. tb Lipidome MS‐LAMP” and “General Lipidome MS‐LAMP” [15]. The LC‐MS data were acquired from the Analyst software in text format. Data sorting was done manually in .xls format where the common masses from all three replicates with a cutoff of 10,000 (*E* ^4^) intensity were put into “Mtb LipidDB” of MS‐LAMP to find mycobacterial specific lipid masses for both positive and negative modes. The mass window range for each search was set to 0.25 and the m/z values in the mass spectra were attributed to singly protonated ions, that is, [M^+^H]^+^ and [M^−^H]^−^, exclusively. The masses identified from both positive and negative ionization modes were combined for further analysis.

#### 2.6.2. Targeted Data Analysis

Matrix‐matched calibration was performed independently for TL and CWL extracts to account for matrix effects and ensure accurate quantification. For this, all TL extracts were pooled to prepare a representative TL‐specific matrix, and all CWL extracts were pooled separately to prepare a CWL‐specific matrix.

A pooled natural standard mixture was prepared using known concentrations of respective TG and DG standards for GL quantification and PC, LPC, PE, PI, and PS standards for GPL quantification. These were serially diluted and subsequently spiked into the respective TL and CWL matrices prior to LC‐MS analysis.

Separate LC‐MS runs were conducted for GL and GPL standard calibration series within each matrix type (TL and CWL), generating independent calibration curves for every lipid subclass. The acquired data were integrated with MultiQuant software (Sciex) v3.0, where each identified peak was smoothened and the background was subtracted. The integrated data were then exported to MS Excel for further statistical analysis. Calibration curves were prepared through internal to natural standard ratios followed by matrix subtraction, ensuring high quantitative accuracy and reproducibility across both TL and CWL datasets. The calibration curves, established for TL and CWL matrices, respectively, for each lipid subclass, each exhibiting excellent linearity (*R* ^2^ = 0.99). These matrix‐specific calibration curves were used to calculate absolute lipid concentrations, ensuring quantitative robustness and cross‐matrix comparability.

### 2.7. Multivariate Statistical Analysis

Untargeted sorted and processed data were uploaded to MetaboAnalyst 6.0 for univariate, multivariate, hierarchical cluster, and biomarker analysis and validation, as explained in Fatima et al. [14]. Student′s *t*‐test (*p* ≤ 0.05) was applied for univariate analysis, whereas principal component analysis (PCA) and partial least squares‐discriminant analysis (PLS‐DA) were performed for multivariate evaluation. Hierarchical clustering was visualized through dendrograms and heat maps, and biomarker screening was conducted using receiver operating characteristic (ROC) curve analysis. Venn diagrams were generated using Venny 2.1, and graphical representations (pie, 3D column, and doughnut charts) were prepared in MS Excel. Statistical robustness and model reliability were supported by high‐multivariate validation metrics (*R* ^2^ > 0.9; *Q* ^2^ > 0.7), significance thresholds (*p* ≤ 0.05, *V* *I* *P* ≥ 1.0), and ROC‐based validation with tenfold cross‐validation to minimize model overfitting, confirming model stability and predictive accuracy.

## 3. Results

### 3.1. Untargeted Lipid Profiling Reveals a Distinct Distribution of Lipid Species in MTB and *M. kansasii* Clinical Isolates

The untargeted IDA lipidome profile analysis, Figure S1 shows typical chromatograms achieved during data acquisition of MTB and *M. kansasii* samples in positive and negative ion modes. The overall identified m/z values ranged from 303.12 to 1820.88 for MTB and 301.2 to 1688.76 for *M. kansasii*. The distribution of these values across the six lipid categories is detailed in Table S1. The analysis of lipid categories was conducted using the lower 0.25 window range of MS‐LAMP to avoid nonspecific identification and to maintain the precision of data and its interpretation. The processed IDA data from the MTB and *M. kansasii* isolates were statistically analyzed using MetaboAnalyst 6.0 for distinction and grouping. The data exhibited distinct MTB and *M. kansasii* lipid profiles and provided a comprehensive overview of the lipid categories at the molecular level, as mentioned in the subsections below.

#### 3.1.1. Covariance of TL and CWL Through Multivariate Statistical Analysis Between MTB and *M. kansasii* Isolates

**Figure 1:**
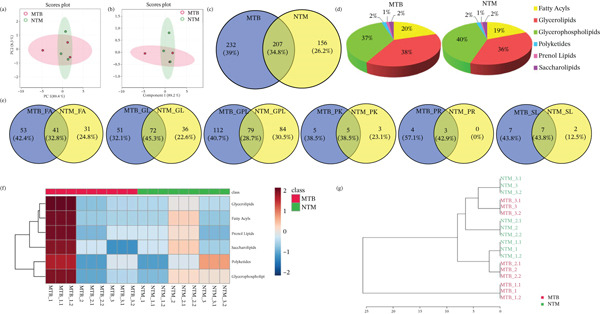
Multivariate and statistical analyses of TL extracts from MTB and NTM (*M. kansasii*) clinical isolates. Score plots of PCA (a) and PLS‐DA (b) showing partial overlap yet distinct clustering trends between MTB (red) and *M. kansasii* (green), indicating compositional divergence in TL lipid profiles. (c) Venn diagram showing exclusive and shared lipids identified across both isolates. (d) Pie charts represent the relative percentage distribution of identified major lipid categories in MTB and NTM (*M. kansasii*) isolates. (e) Venn diagrams showing unique and common lipids among lipid categories, that is, fatty acyls (FA), glycerolipids (GL), glycerophospholipids (GLP), polyketides (PK), prenol lipids (PR), and saccharolipids (SL). (f) The clustering heat map generated from MetaboAnalyst 6.0 indicating relative abundance patterns of lipid categories in MTB and NTM (*M. kansasii*) isolates, where brown color indicates higher and blue color indicates low abundance. (g) Hierarchical clustering dendrogram demonstrating clear species‐level grouping and intraspecies similarity based on TL profiling.

Using unsupervised PCA and PLS‐DA, we evaluated the covariance in the TL extracts of MTB and *M. kansasii* isolates. PCA demonstrated an explained variance of 89.4% for PC1 and 8.3% for PC2, accounting for 97.7% of the total variance (Figure 1a). PLS‐DA showed a covariance of 89.2% for PC1 and 5.1% for PC2, summing to 94.3% of the total covariance (Figure 1b). The Venn diagram revealed 39% unique lipids identified in MTB isolates, whereas 26.2% in *M. kansasii* isolates and 34.8% common to both the isolates (Figure 1c). The list of identified lipids in the Venn diagram with their scientific names is shown in S. Sheet 1. Figure 1d illustrates the percentage distribution of identified lipids in six lipid categories viz. FA, GL, GPL, PK, PR, and SL within MTB and *M. kansasii*, respectively. FA, GL, and GPL constituted the primary lipid categories, accounting for 95% of all identified lipids in both MTB and *M. kansasii*. All lipid categories were abundantly present in MTB isolates than in *M. kansasii* isolates, whereas PR lipids were exclusively present in MTB isolates (Figure 1e). The list of identified lipids in the Venn diagram, with their scientific names, is shown in S. Sheet 1. Further, the lipid distribution pattern was analyzed within the lipid categories through a heat map and cluster dendrogram, as shown in Figure 1f,g, respectively. The dendrogram illustrates the lipid profile based on the hierarchical relationship between MTB and *M. kansasii* clinical isolates. In the TL extracts of bacilli, two replicates of MTB and *M. kansasii* were closely related, making the differentiation less distinct.

Similarly, the TL extract, unsupervised PCA and PLS‐DA were performed to evaluate the covariance of the first five components in the CWL extract of MTB and *M. kansasii* isolates. PCA revealed an explained variance of 69% for PC1 and 21.6% for PC2, accounting for 90.6% of the total variance (Figure 2a). PLS‐DA showed a covariance of 68.6% for PC1 and 8.2% for PC2, summing to 76.8% of the total covariance (Figure 2b). The Venn diagram represented 28.9% unique lipids identified to MTB isolates conversely 34.6% in *M. kansasii* isolates, and 36.5% common to both isolates (Figure 2c). The list of identified lipids in Venn diagram with their scientific names is appended in S. Sheet 2. The percentage distribution of all six lipid categories in MTB and *M. kansasii* is illustrated in Figure 2d, where FA, GL, and GPL constituted 96% of identified lipids in MTB and 94% in *M. kansasii*. MTB isolates contained a higher percentage of FA than the *M. kansasii* isolates, whereas other lipid categories, viz. GL, GPL, PK, PR, and SL were higher in *M. kansasii* than MTB isolates (Figure 2e). The list of identified lipids in the Venn diagram with their scientific names is shown in S. Sheet 2. Additionally, a heat map (Figure 2f) and cluster dendrogram (Figure 2g) were utilized to identify patterns and relationships between MTB and *M. kansasii* isolates based on their lipid compositions. Figure 2f also shows that FAs were elevated in MTB isolates, whereas GL, GPL, PK, PR, and SL lipids were high in *M. kansasii* isolates. Unlike the bacilli TL extract, CWL extract displayed clear differentiation between MTB and *M. kansasii* isolates as depicted in Figure 2g. Interestingly, the hierarchical clustering dendrogram generated by Metaboanalyst 6.0 shows distinct clustering of MTB and *M. kansasii isolates* based on the identified lipid moieties.

Figure 2Multivariate and statistical analyses of CWL extracts from MTB and NTM (*M. kansasii*) clinical isolates. Score plots of PCA (a) and PLS‐DA (b) showing distinct species‐level grouping based on cell wall lipid composition. (c) Venn diagram showing exclusive and shared lipids identified in MTB and NTM (*M. kansasii*) isolates. (d) Pie charts representing the relative percentage distribution of identified major lipid categories in cell wall lipid extracts of both isolates. (e) Venn diagrams showing unique and common lipids among lipid categories, that is, fatty acyls (FA), glycerolipids (GL), glycerophospholipids (GLP), polyketides (PK), prenol lipids (PR), and saccharolipids (SL). (f) The clustering heat map generated from MetaboAnalyst 6.0 indicates the relative abundance pattern of lipid categories, where brown color indicates higher and blue color indicates low abundance. (g) Hierarchical clustering dendrogram demonstrating distinct species‐specific grouping, confirming compositional divergence between MTB and *M. kansasii* based on CWL profiling.

**Figure:**
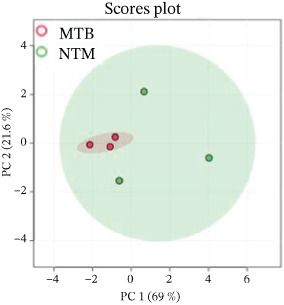
(a)

**Figure:**
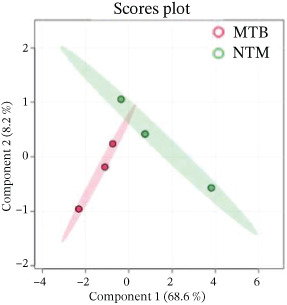
(b)

**Figure:**
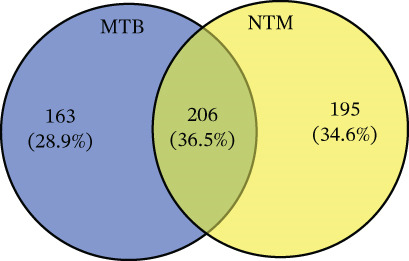
(c)

**Figure:**
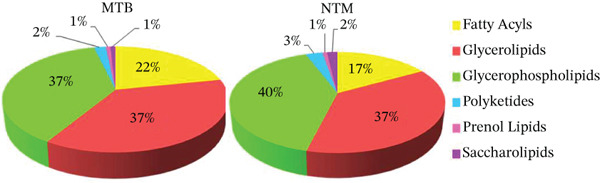
(d)

**Figure:**
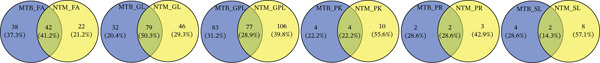
(e)

**Figure:**
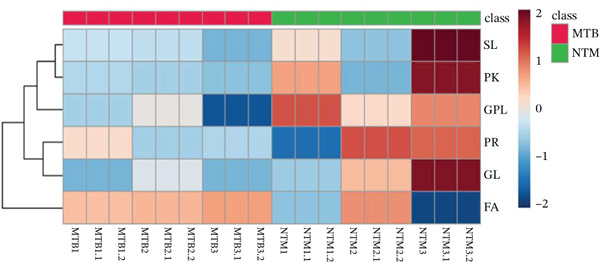
(f)

**Figure:**
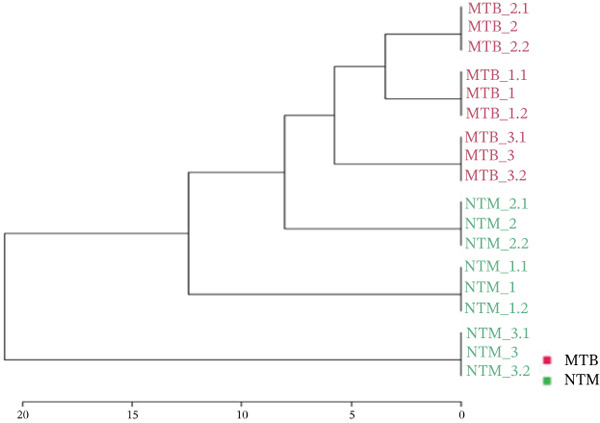
(g)

#### 3.1.2. Abundance of Lipid Subclasses in TL and CWL Extracts of MTB and *M. kansasii* Isolates

Figure 3a illustrates the comparative abundance of various lipid subclasses in the TL extract of MTB and *M. kansasii* isolates, whereas Figure 3b presents various lipid subclasses for the CWL extract. Table S2 provides a list of the identified numbers of these lipid subclasses in TL and CWL extracts of MTB and *M. kansasii* isolates. In the FA lipid category of TL extract, MTB exhibits higher levels of branched fatty acids, keto‐MA, methoxy‐MA, alpha‐MA, DIMA, TMM, and DIMB compared with *M. kansasii*, whereas GMM was equally present in both isolates. Within the GL category, MG, DG, and TG subclasses were higher in MTB compared with *M. kansasii.* For the GPL category, MTB isolates showed higher levels of Lyso‐PG, Lyso‐PI, Lyso‐PIM1, PG, CL, PI, Ac1PIM1, and Ac1PIM3 than *M. kansasii*; Lyso‐PIM3, Lyso‐PIM4, Lyso‐PIM5, Lyso‐PIM6, PIM5, and Ac2PIM2 were exclusive to MTB isolates; conversely, *M. kansasii* isolates had marginally higher levels of Lyso‐PE, PE, PIM1, and PIM2, whereas Lyso‐PIM2, PIM3, PIM4, and Ac1PIM2 were equally distributed in both isolates. In the PK lipid category, mannosyl‐*β*1‐phosphomycoketides were found only in MTB, whereas nonribosomal peptides/PK hybrids were high in *M. kansasii* isolates. Ubiquinones were marginally higher in MTB in the PR category, and bactoprenols were exclusive to *M. kansasii*. In the SL category, Ac2SGL was prevalent in MTB, whereas DAT1 and DAT2 were equally distributed in both isolates.

Figure 3Histograms showing distribution of lipid subclasses among TL and CWL extracts of the MTB and NTM (*M. kansasii*). (a) Histograms illustrate compositional differences in lipid subclasses across six categories, that is, FA, GL, GLP, PK, PR, and SL, in TL (a) and CWL (b) extracts of MTB and NTM (*M. kansasii*) isolates.

**Figure:**
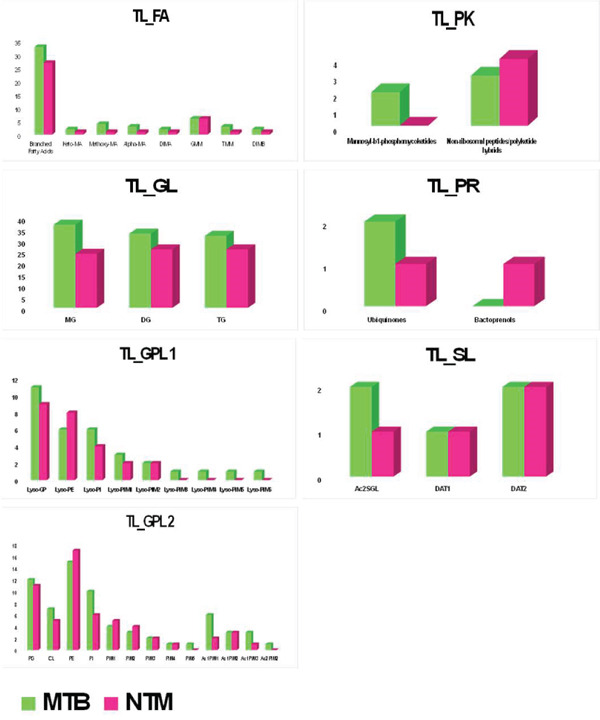
(a)

**Figure:**
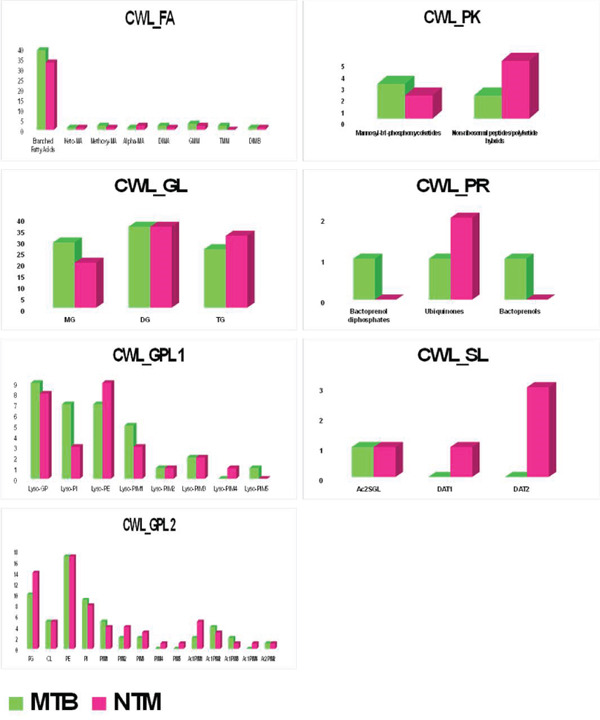
(b)

The CWL extract of MTB showed higher levels of branched FA, methoxy‐MA, DIMA, TMM, and GMM compared with *M. kansasii*; alpha‐MA was significantly abundant in *M. kansasii,* whereas keto‐MA and DIMB were equally present in both isolates. In the GL category, MG was higher in MTB, TG was higher in *M. kansasii*, and DG was equally present in both isolates. For the GPL category, MTB isolates exhibited marginally higher levels of Lyso‐GP, Lyso‐PI, Lyso‐PIM1, Lyso‐PIM5, PI, PIM1, Ac1PIM2, and Ac1PIM3 than *M. kansasii*; *M. kansasii* showed higher levels of Lyso‐PE, PG, PIM2, PIM3, and Ac1PIM1, whereas Lyso‐PIM4, PIM4, PIM5, and Ac1PIM4 were exclusive to *M. kansasii*, whereas Lyso‐PIM2, Lyso‐PIM3, CL, PE, and Ac2PIM2 were equally present in both isolates. In the PK lipid category, mannosyl‐*β*1‐phosphomycoketides were marginally higher in MTB, whereas nonribosomal peptides/PK hybrids were abundant in *M. kansasii* isolates. For the PR category, ubiquinones were slightly higher in *M. kansasii*, whereas bactoprenols and bactoprenol diphosphates were found only in MTB. In the SL category, Ac2SGL was equally present in both isolates, whereas DAT1 and DAT2 were exclusive to *M. kansasii*.

#### 3.1.3. Distribution of Identified Unique and Common Lipid Species in TL and CWL Extracts of MTB and *M. kansasii* Isolates

**Figure 4:**
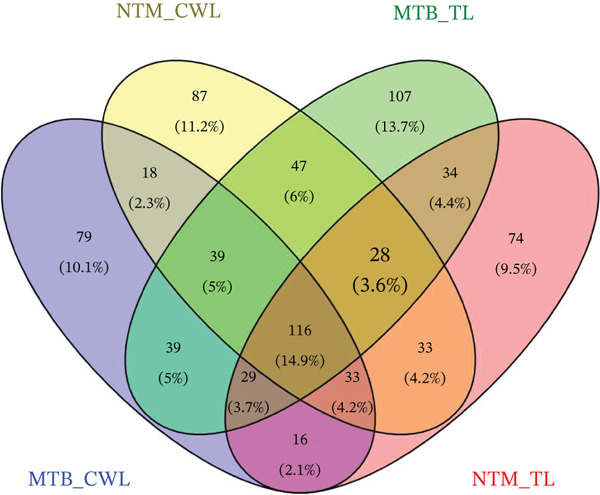
A comparative Venn diagram of TL and CWL extracts from MTB and NTM (*M. kansasii*). The four‐way Venn diagram shows the distribution of unique and shared lipid numbers with percentages across TL and CWL extracts of both isolates.

The distribution of identified lipid molecules in MTB and *M. kansasii* isolates was analyzed through the Venn diagram (Figure 4). In the TL extract, 13.7% of lipids were unique to MTB, whereas 9.5% were unique to *M. kansasii*, and 4.4% were shared between the clinical isolates. In the CWL extract, 10.1% of lipids were exclusive to MTB, whereas 11.2% were exclusive to *M. kansasii*, and 2.3% were common to both the clinical isolates. Additionally, 5% of lipids were shared between MTB TL and CWL extracts, whereas 4.2% were shared between *M. kansasii* TL and CWL extracts. A total of 14.9% of the lipids were common across all TL and CWL extracts. Further, 6% of lipids were shared between *M. kansasii* CWL and MTB TL extracts, 2.1% between MTB′s CWL and *M. kansasii′s* TL extracts, 3.7% among MTB and *M. kansasii′s* TL extracts and MTB′s CWL extracts, 3.6% among MTB and *M. kansasii′s* TL extracts and *M. kansasii′s* CWL extracts, 5% among MTB and *M. kansasii′s* CWL extracts and MTB′s TL extract, and 4.2% among MTB and *M. kansasii′s* CWL extracts and *M. kansasii′s* TL extract. The list of identified lipids in the Venn diagram with their scientific names is enclosed in S. Sheet 3.

### 3.2. MRM‐Based Targeted Lipidomics Exhibits Absolute Quantification of GL and GPL Lipid Species Among MTB and *M. kansasii* Isolates

The data achieved from untargeted lipid profiling indicated significant variations in GL and GPL lipid classes. Therefore, the quantification of TG and DG molecular species from the GL lipid category and PC, LPC, PE, PI, and PS from the GPL lipid category was done through MRM‐based targeted lipid profiling. The optimized LC‐MS parameters for internal standard Splash and their respective natural standards, viz. TG, DG, PC, LPC, PE, PI, and PS are mentioned in Table S3. The Q1 was scanned in the m/z range of 100–900 Da to obtain the mass spectrum of all the standards in positive ion modes (Figure S2a). The MS2 scan of each parent ion was performed to obtain the optimum fragmentation pair, as shown in Figure S2b. The XICs for all standards are illustrated in Figure S2c. The LOD and LOQ ranged from 45.76 to 109.86 ng/mL and 138.66 to 332.99 ng/mL, respectively. LOD and LOQ for individual natural standards are provided in Table S4. For the absolute quantification of various GL and GPL lipid species from TL and CWL extracts, we incorporated 125 transition pairs for GL, including 42 for TG and 83 for DG, as well as 71 transition pairs for GPL, consisting of 18 for PC, 10 for LPC, seven for PE, 24 for PI, and 12 for PS molecular species. These transition pairs are detailed, along with their respective lipid IDs, in Table S5. However, in the TL extract, a total of 99 GL lipid molecules were successfully quantified, comprising 41 TG and 58 DG, along with 65 GPL lipid species, including 18 PC, eight LPC, seven PE, 23 PI, and nine PS. In contrast, the CWL extract yielded 71 GL lipid species, consisting of 27 TG and 44 DG, and 29 GPL lipid species, including 11 PC, nine LPC, seven PE, and two PI. The absolute concentrations of these lipid species, expressed in ng/mL of 1 OD cells, are provided in Table S6. The percentage distribution of differentially expressed GL and GPL lipid species in TL and CWL extracts from both clinical isolates was analyzed and illustrated through pie charts (Figure S3). Figure S4a,b illustrates the relative abundance in the percentage of all quantified GL and GPL lipid species in MTB and *M. kansasii* clinical isolates among TL and CWL extracts, respectively.

#### 3.2.1. Distribution of Quantified GL and GPL Lipid Species in TL and CWL Extracts of MTB and *M. kansasii* Isolates, Along With Differential Expression

**Figure 5:**
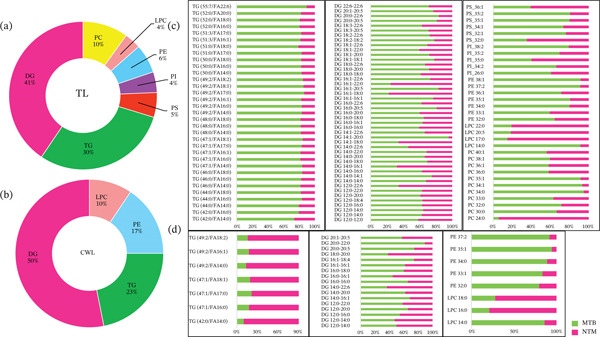
Distribution of significant quantified lipids of GL (TG and DG) and GPL (PC, LPC, PE, PI, and PS) subclasses in TL and CWL extracts of both isolates. (a) and (b) Doughnut charts show relative distribution percentage of statistically significant (*p* ≤ 0.05) quantified GL and GPL lipid species in TL and CWL extracts, respectively. Bar graphs (c) and (d) exhibit the comparative abundance of individual TG, DG, PC, LPC, PE, PI, and PS lipid species between MTB and NTM (*M. kansasii*) isolates in TL and CWL extracts, respectively.

Further, the *p* values of all quantified lipids were calculated and those lipids having *p* values of ≤ 0.05 were only considered as statistically significant. Table 1 provides the absolute concentration of significant lipid molecular species from GL and GPL categories. The percentage distribution of significantly differentially expressed GL and GPL lipid molecules in TL and CWL extracts from both MTB and *M. kansasii* clinical isolates is illustrated in doughnut charts (Figure 5a,b). TL extracts exhibited a high percentage of DGs followed by TG, PC, PE, PS, PI, and LPC, whereas CWL extracts also showed the highest percentage of DG than TG, PE, and LPC. The PC and PI lipid molecules were not significantly identified. Figure 5c,d illustrates the percentage distribution of significantly quantified GL and GPL lipid species in both MTB and *M. kansasii* clinical isolates. In TL extract, a total of 78, including 33 TG and 45 DG lipid species in the GL lipid category and 33 GPL lipid species comprising 11 PC, four LPC, seven PE, five PI, and six PS were found significant. In contrast, a sum of 22 GL having seven TG and 15 DG lipid species and eight GPL including three LPC, and five PE, having *p* values of ≤ 0.05 in CWL extract. The data in Figure 5 revealed that TL extracts contained a higher number of significant GL and GPL lipid moieties compared with the CWL extracts.

**Table 1:** Absolute concentrations of quantified GL and GPL lipid species based on ≤ 0.05 *p* values.

**(A) TL extract**								
**Analyte (lipid species)**	**MTB (ng/mL)**	**NTM (*M. kansasii*) (ng/mL)**	**Analyte (lipid species)**	**MTB (ng/mL)**	**NTM (*M. kansasii*) (ng/mL)**	**Analyte (lipid species)**	**MTB (ng/mL)**	**NTM (*M. kansasii*) (ng/mL)**
TG (42:0/FA14:0)	3.55	1.26	DG 12:0–12:0	0.63	0.45	PC 24:0	0.02	0.28
TG (42:0/FA16:0)	6.35	1.63	DG 12:0–14:0	0.55	0.32	PC 30:0	1.26	0.64
TG (44:0/FA14:0)	3.85	0.99	DG 12:0–14:0	0.49	0.27	PC 32:0	4.01	1.61
TG (44:0/FA16:0)	5.96	1.18	DG 12:0–16:0	0.24	0.14	PC 33:0	0.43	0.25
TG (44:0/FA18:0)	2.91	0.64	DG 12:0–18:4	0.02	0.01	PC 34:0	7.07	0.38
TG (46:0/FA14:0)	3.32	0.75	DG 12:0–20:0	0.37	0.24	PC 34:1	69.88	5.05
TG (46:0/FA16:0)	5.76	1.21	DG 12:0–22:0	0.20	0.12	PC 35:1	16.25	1.51
TG (46:0/FA18:0)	2.27	0.44	DG 12:0–22:6	0.87	1.66	PC 36:0	1.24	0.90
TG (47:1/FA14:0)	0.63	0.12	DG 14:0–14:0	0.21	0.10	PC 36:1	13.63	9.87
TG (47:1/FA16:0)	1.03	0.23	DG 14:0–14:1	0.05	0.02	PC 38:1	0.81	0.55
TG (47:1/FA16:1)	1.15	0.26	DG 14:0–16:0	0.38	0.23	PC 40:1	0.06	0.05
TG (47:1/FA17:0)	0.29	0.06	DG 14:0–16:1	0.16	0.34	LPC 14:0	0.30	0.03
TG (47:1/FA18:1)	0.69	0.13	DG 14:0–18:0	0.45	0.20	LPC 17:0	0.86	4.95
TG (48:0/FA14:0)	1.76	0.38	DG 14:0–20:0	0.16	0.08	LPC 20:5	1.45	6.40
TG (48:0/FA16:0)	6.23	1.36	DG 14:0–22:0	0.08	0.04	LPC 22:0	0.04	0.15
TG (48:0/FA18:0)	2.73	0.61	DG 14:0–22:6	1.04	1.21	PE 32:0	25.13	13.94
TG (49:2/FA14:0)	0.10	0.02	DG 14:1–18:0	0.11	0.21	PE 33:1	24.57	16.94
TG (49:2/FA16:0)	0.35	0.07	DG 14:1–20:0	0.96	0.04	PE 34:0	21.31	5.38
TG (49:2/FA16:1)	0.86	0.18	DG 14:1–22:6	0.03	0.02	PE 35:1	55.14	13.35
TG (49:2/FA17:0)	0.18	0.03	DG 16:0–16:0	7.72	5.25	PE 36:1	7.05	2.86
TG (49:2/FA18:1)	0.62	0.11	DG 16:0–16:1	0.73	0.41	PE 37:2	16.73	1.62
TG (49:2/FA18:2)	0.31	0.06	DG 16:0–18:0	4.85	2.60	PE 38:1	0.44	0.04
TG (50:0/FA14:0)	0.58	0.12	DG 16:0–20:0	0.34	0.22	PI_26:0	0.68	0.44
TG (50:0/FA16:0)	4.01	0.85	DG 16:0–20:5	0.50	0.12	PI_34:2	0.28	0.14
TG (50:0/FA18:0)	3.63	0.76	DG 16:0–22:6	1.68	1.01	PI_35:0	0.83	1.23
TG (51:0/FA17:0)	0.08	0.02	DG 16:1–16:1	0.46	0.14	PI_35:2	0.10	0.04
TG (51:0/FA18:0)	0.23	0.06	DG 16:1–18:0	1.59	5.60	PI_38:2	0.02	0.01
TG (51:3/FA16:1)	0.14	0.03	DG 16:1–20:5	0.14	0.03	PS_32:0	0.01	0.02
TG (51:3/FA17:0)	0.02	0.00	DG 16:1–22:0	0.01	0.03	PS_32:1	0.01	0.00
TG (52:0/FA16:0)	1.15	0.27	DG 16:1–22:6	0.09	0.03	PS_34:1	0.02	0.01
TG (52:0/FA18:0)	2.42	0.58	DG 18:0–18:0	3.17	1.99	PS_35:1	0.00	0.00
TG (52:0/FA20:0)	0.09	0.02	DG 18:0–20:0	0.11	0.04	PS_35:2	0.01	0.00
TG (55:7/FA22:6)	0.01	0.00	DG 18:0–22:6	0.91	0.46	PS_36:1	0.00	0.01
			DG 18:1–18:1	5.38	1.53			
			DG 18:1–20:0	0.03	0.01			
			DG 18:1–22:0	0.03	0.02			
			DG 18:1–22:6	1.13	0.50			
			DG 18:2–18:2	0.31	0.09			
			DG 18:2–22:6	0.12	0.04			
			DG 18:3–20:5	0.01	0.01			
			DG 18:3–22:6	0.23	0.14			
			DG 20:0–20:5	0.06	0.01			
			DG 20:0–22:6	0.13	0.03			
			DG 20:1–20:5	0.03	0.01			
			DG 22:6–22:6	0.41	0.17			

**(B) CWL extract**								
**Analyte (lipid species)**	**MTB (ng/mL)**	**NTM (*M. kansasii*) (ng/mL)**	**Analyte (lipid species)**	**MTB (ng/mL)**	**NTM (*M. kansasii*) (ng/mL)**	**Analyte (lipid species)**	**MTB (ng/mL)**	**NTM (*M. kansasii*) (ng/mL)**

TG (42:0/FA14:0)	37.67	304.30	DG 12:0–14:0	3.20	3.23	LPC 14:0	8.47	1.36
TG (47:1/FA16:0)	5.28	21.61	DG 12:0–14:0	2.02	2.26	LPC 16:0	47.20	177.25
TG (47:1/FA17:0)	1.95	6.18	DG 12:0–16:0	2.98	2.49	LPC 18:0	20.20	51.76
TG (47:1/FA18:1)	3.27	12.04	DG 12:0–20:0	12.51	6.54	PE 32:0	0.73	0.18
TG (49:2/FA14:0)	0.25	1.40	DG 12:0–22:0	5.15	3.68	PE 33:1	0.90	0.18
TG (49:2/FA16:1)	2.72	11.33	DG 14:0–16:1	6.66	3.08	PE 34:0	0.61	0.07
TG (49:2/FA18:2)	0.80	3.80	DG 14:0–20:0	5.88	3.58	PE 35:1	1.75	0.10
			DG 14:0–22:6	4.17	7.25	PE 37:2	0.30	0.03
			DG 16:0–16:0	310.36	162.73			
			DG 16:0–16:1	11.35	14.14			
			DG 16:0–18:0	324.84	217.13			
			DG 16:1–16:1	8.37	5.20			

### 3.3. Screening and Establishment of Lipid Biomarkers of MTB and M. kansasii

Firstly, the data were sorted and processed for both the extracts of MTB and *M. kansasii* isolates and analyzed by using classical univariate ROC curve analysis to identify biosignatures. This process involved calculating the area under the curve (AUC) and identifying the sensitivity and specificity at the optimal cutoff point based on the Youden index. To strengthen the reliability of our results, we utilized the bootstrap method to determine the 95% confidence interval (CI) for the AUC. An AUC value of 1.0 was considered perfect for both lipid extracts. All statistical analyses were conducted using MetaboAnalyst 6.0 web‐based software, with statistical significance defined as a *p* value of ≤ 0.05. In TL extracts, a total of 36 lipid molecules were identified comprising six FAs, 13 GLs, and 17 GPLs, whereas 38 lipids screened out from the CWL extracts including 14 FAs, 16 GLs, six GPLs, and two PKs with 1.0 AUC. Among these five lipid species, namely MG (RCO2H = 18 : 1), TG (R1CO2H + R2CO2H + R3CO2H = 50 : 1), TG (R1CO2H + R2CO2H + R3CO2H = 51 : 0), hydroxyphthioceranic acid (C33), and Lyso‐PG (RCO2H = 18 : 1) were found to be common between both TL and CWL extracts with same trend (Figure 6). MG (RCO2H = 18 : 1) was found exclusively in both extracts of MTB isolates, whereas TG (R1CO2H + R2CO2H + R3CO2H = 50 : 1), TG (R1CO2H + R2CO2H + R3CO2H = 51 : 0), hydroxyphthioceranic acid (C33), and Lyso‐PG (RCO2H = 18 : 1) were present only in *M. kansasii* isolates.

Figure 6Box plots and ROC curves displaying discriminative lipid biomarkers identified in TL and CWL extracts of MTB and NTM (*M. kansasii*). (a) (Panels A, B, and C) TL‐derived lipid biomarkers from the fatty acyl (FA), glycerolipid (GL), and glycerophospholipid (GPL) categories, respectively, each showing perfect discrimination between MTB and *M. kansasii* (AUC = 1.0; 95% CI). (b) (Panels D, E, F, and G) corresponding CWL biomarkers for FA, GL, GPL, and polyketide (PK) classes, also achieving AUC = 1.0 with 95% confidence intervals, confirming their strong diagnostic potential.

**Figure:**
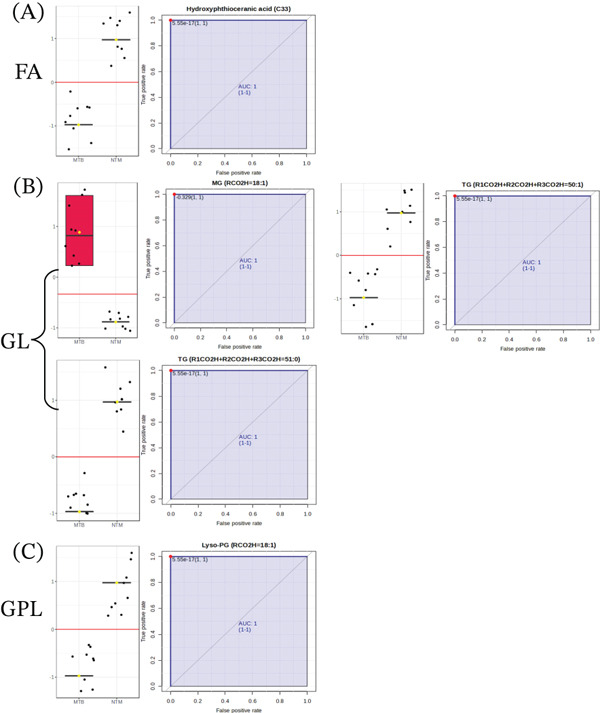
(a)

**Figure:**
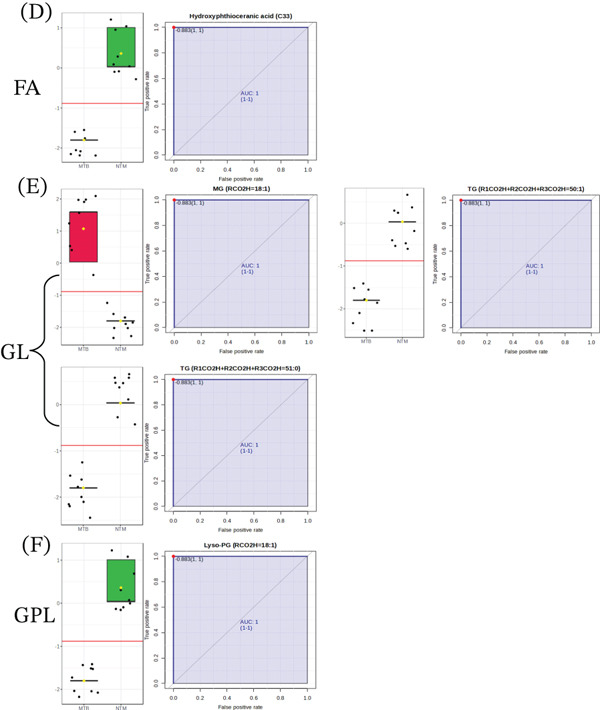
(b)

**Figure 7:**
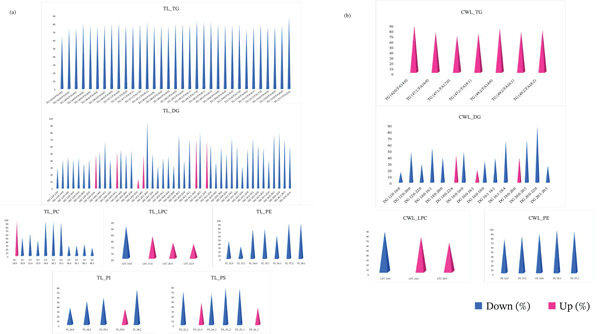
Histograms depicting differential regulation of significant quantified lipid species from GL and GPL categories in TL and CWL extracts of MTB and NTM (*M. kansasii*) isolates. Histograms represent the relative percentage of upregulation (pink color) and downregulation (blue color) of significant quantified lipid species from GL and GPL categories in TL (a) and CWL (b) extracts, respectively.

Subsequently, targeted lipid profiling was evaluated to assess the differential regulation of quantified lipid species, with statistical significance defined at *p* ≤ 0.05. In the TL extract, 33 TG and 39 DG lipid species were downregulated, whereas six DG lipid species were upregulated in *M. kansasii*. Furthermore, 10 PC, one LPC, seven PE, four PI, and four PS lipid species were downregulated, whereas one PC, three LPC, one PI, and two PS lipid species were upregulated in *M. kansasii* as depicted in Figure 7a. Figure 7b illustrates the lipid changes with *p* values of ≤ 0.05 in the CWL extract, where seven TG, three DG, and and LPC lipid species were significantly upregulated, whereas 12 DG, one LPC, and five PE lipid species were downregulated in *M. kansasii*. Nine DGs, one LPC, and five PEs lipid molecular species were observed in both TL and CWL extracts; however, PC, PI, and PS lipid species were exclusively found in the TL extract. A comprehensive list of the differentially expressed lipids in TL and CWL extracts is provided in Table 2. Lipid biomarkers identified with names from untargeted and targeted biomarker analysis are provided in Table 3.

**Table 2:** Up and down percentages of quantified lipid species from GL and GPL lipid categories.

**(A) TL extract**											
**Analyte (lipid species)**	**p**	**Down (%)**	**Up (%)**	**Analyte (lipid species)**	**p**	**Down (%)**	**Up (%)**	**Analyte (lipid species)**	**p**	**Down (%)**	**Up (%)**
TG (42:0/FA14:0)	0.00023	64.35		DG 12:0–12:0	0.029352	28.22		PC 24:0	0.000621		93.97
TG (42:0/FA16:0)	0.000586	74.32		DG 12:0–14:0	0.001311	41.05		PC 30:0	0.001168	49.47	
TG (44:0/FA14:0)	2.03E‐05	74.32		DG 12:0–14:0	0.003985	44.41		PC 32:0	0.000229	59.94	
TG (44:0/FA16:0)	2.95E‐05	80.28		DG 12:0–16:0	0.000361	41.24		PC 33:0	0.005001	41.32	
TG (44:0/FA18:0)	5.89E‐05	78.15		DG 12:0–18:4	0.016275	43.44		PC 34:0	0.000435	94.58	
TG (46:0/FA14:0)	1.78E‐05	77.43		DG 12:0–20:0	0.035837	35.91		PC 34:1	2.07E‐05	92.77	
TG (46:0/FA16:0)	3.85E‐07	78.95		DG 12:0–22:0	0.006121	41.43		PC 35:1	0.000113	90.69	
TG (46:0/FA18:0)	9.98E‐06	80.66		DG 12:0–22:6	0.000854		47.74	PC 36:0	0.025692	27.84	
TG (47:1/FA14:0)	5.64E‐07	80.74		DG 14:0–14:0	0.00148	52.57		PC 36:1	0.003165	27.60	
TG (47:1/FA16:0)	0.000156	77.37		DG 14:0–14:1	0.000356	66.86		PC 38:1	0.005026	31.98	
TG (47:1/FA16:1)	3.43E‐05	77.38		DG 14:0–16:0	0.005746	40.62		PC 40:1	0.013603	21.65	
TG (47:1/FA17:0)	4.37E‐07	79.93		DG 14:0–16:1	0.000258		52.50	LPC 14:0	0.00375	90.46	
TG (47:1/FA18:1)	2.75E‐06	81.61		DG 14:0–18:0	0.000455	55.88		LPC 17:0	0.004508		82.58
TG (48:0/FA14:0)	1.86E‐05	78.35		DG 14:0–20:0	0.010097	49.06		LPC 20:5	0.014907		77.35
TG (48:0/FA16:0)	0.000205	78.20		DG 14:0–22:0	0.001954	54.22		LPC 22:0	0.030272		76.38
TG (48:0/FA18:0)	1.84E‐05	77.65		DG 14:0–22:6	0.037084		13.70	PE 32:0	6.62E‐05	44.53	
TG (49:2/FA14:0)	2.39E‐05	79.99		DG 14:1–18:0	0.023294		47.91	PE 33:1	0.000385	31.02	
TG (49:2/FA16:0)	7.99E‐06	79.89		DG 14:1–20:0	0.000115	95.45		PE 34:0	5E‐05	74.77	
TG (49:2/FA16:1)	7.62E‐06	78.95		DG 14:1–22:6	0.000856	49.34		PE 35:1	4.45E‐06	75.78	
TG (49:2/FA17:0)	2.35E‐05	84.26		DG 16:0–16:0	0.021656	31.97		PE 36:1	0.001004	59.50	
TG (49:2/FA18:1)	1.93E‐06	82.95		DG 16:0–16:1	0.000384	43.31		PE 37:2	1.11E‐05	90.32	
TG (49:2/FA18:2)	1.57E‐05	82.08		DG 16:0–18:0	0.005168	46.41		PE 38:1	3.51E‐05	90.65	
TG (50:0/FA14:0)	6.25E‐07	79.67		DG 16:0–20:0	0.002361	33.51		PI_26:0	0.004772	34.85	
TG (50:0/FA16:0)	6.68E‐06	78.84		DG 16:0–20:5	0.000192	76.61		PI_34:2	0.033126	48.54	
TG (50:0/FA18:0)	2.49E‐06	78.96		DG 16:0–22:6	0.000556	39.97		PI_35:0	0.020753		33.05
TG (51:0/FA17:0)	9.39E‐05	79.74		DG 16:1–16:1	3.95E‐05	69.58		PI_35:2	0.029939	55.93	
TG (51:0/FA18:0)	9.88E‐05	73.33		DG 16:1–18:0	3.36E‐05		71.65	PI_38:2	0.002626	73.60	
TG (51:3/FA16:1)	1.47E‐05	77.43		DG 16:1–20:5	1.2E‐05	79.91		PS_32:0	0.024165		45.71
TG (51:3/FA17:0)	5.98E‐06	79.11		DG 16:1–22:0	0.029591		67.68	PS_32:1	0.028797	68.55	
TG (52:0/FA16:0)	1.46E‐05	76.15		DG 16:1–22:6	0.00079	63.11		PS_34:1	0.037013	63.97	
TG (52:0/FA18:0)	1.51E‐05	76.04		DG 18:0–18:0	0.009176	37.13		PS_35:1	0.042851	74.80	
TG (52:0/FA20:0)	1.78E‐05	78.54		DG 18:0–20:0	0.002667	60.38		PS_35:2	0.038263	75.60	
TG (55:7/FA22:6)	1.33E‐05	88.30		DG 18:0–22:6	0.000155	50.15		PS_36:1	0.009635		35.64
				DG 18:1–18:1	1.34E‐05	71.53					
				DG 18:1–20:0	0.003263	60.95					
				DG 18:1–22:0	0.045712	31.72					
				DG 18:1–22:6	0.000636	56.18					
				DG 18:2–18:2	2.88E‐05	71.81					
				DG 18:2–22:6	0.000245	61.87					
				DG 18:3–20:5	0.046063	58.91					
				DG 18:3–22:6	0.003382	40.46					
				DG 20:0–20:5	0.001287	76.80					
				DG 20:0–22:6	7.23E‐05	80.28					
				DG 20:1–20:5	0.017775	69.93					
				DG 22:6–22:6	3.76E‐05	59.89					
**(B) CWL extract**											
**Analyte (lipid species)**	**p**	**Down (%)**	**Up (%)**	**Analyte (lipid species)**	**p**	**Down (%)**	**Up (%)**	**Analyte (lipid species)**	**p**	**Down (%)**	**Up (%)**
TG (42:0/FA14:0)	0.036155		87.62	DG 12:0–16:0	0.030818	16.27		LPC 14:0	0.00061	83.92	
TG (47:1/FA16:0)	0.003706		75.55	DG 12:0–20:0	0.005633	47.74		LPC 16:0	0.038686		73.37
TG (47:1/FA17:0)	0.006948		68.52	DG 12:0–22:0	0.002278	28.47		LPC 18:0	0.041112		60.97
TG (47:1/FA18:1)	0.015121		72.89	DG 14:0–16:1	0.002179	53.80		PE 32:0	8.42E‐07	74.62	
TG (49:2/FA14:0)	0.024774		82.40	DG 14:0–20:0	0.022196	39.05		PE 33:1	3.5E‐07	80.41	
TG (49:2/FA16:1)	0.013818		75.99	DG 14:0–22:6	0.021517		42.43	PE 34:0	1.67E‐05	87.77	
TG (49:2/FA18:2)	0.026838		78.93	DG 16:0–16:0	0.00364	47.57		PE 35:1	3.58E‐06	94.19	
				DG 16:0–16:1	0.042504		19.73	PE 37:2	0.00041	91.12	
				DG 16:0–18:0	0.005507	33.16					
				DG 16:1–16:1	0.044248	37.85					
				DG 16:1–18:4	0.029947	65.11					
				DG 18:0–20:0	0.025454		38.91				
				DG 20:0–20:5	0.007493	66.02					
				DG 20:0–22:0	6.55E‐05	88.05					
				DG 20:1–20:5	0.003728	26.11					

**Table 3:** Biomarker panel depicting category‐wise scientific names constructed from lipids differentially identified between MTB and NTM (*M. kansasii*) isolates.

**(A) Untargeted IDA based**					
**Common lipid signatures in TL and CWL extracts**					
Hydroxyphthioceranic acid (C33)	MG (RCO2H = 18:1)		Lyso‐PG (RCO2H = 18:1)		
	TG (R1CO2H + R2CO2H + R3CO2H = 50:1)				
	TG (R1CO2H + R2CO2H + R3CO2H = 51:0)				
Exclusive lipid signatures in TL extract					
FA	GL		GPL		
Hydroxyphthioceranic acid (C34)	MG (RCO2H = 17:0)		Lyso‐PE (R1CO2H = 16:0)		
Phthioceranic acid (C36)	MG (RCO2H = 15:0)		Lyso‐PE (R1CO2H = 16:1)		
Phthioceranic acid (C45)	TG (R1CO2H + R2CO2H + R3CO2H = 50:2)		Lyso‐PE (R1CO2H = 18:0)		
Mycocerosic acid (C29)	DG (R1CO2H + R2CO2H = 28:0)		Lyso‐PE (R1CO2H = 17:0)		
Mycolipenic acid (C29)	DG (R1CO2H + R2CO2H = 29:0)		Lyso‐PI (RCO2H = 19:2)		
	DG (R1CO2H + R2CO2H = 29:1)		Lyso‐PG (RCO2H = 18:0)		
	DG (R1CO2H + R2CO2H = 33:0)		Lyso‐PG (RCO2H = 19:0)		
	DG (R1CO2H + R2CO2H = 39:2)		Lyso‐PG (RCO2H = 19:1)		
	DG (R1CO2H + R2CO2H = 52:0)		Lyso‐PG (RCO2H = 20:0)		
	DG (R1CO2H + R2CO2H = 30:1)		PG (R1CO2H + R2CO2H = 31:2)		
			PE (R1CO2H + R2CO2H = 31:0)		
			PE (R1CO2H + R2CO2H = 37:0)		
			Lyso‐PIM1 (RCO2H = 19:2)		
			PI (R1CO2H + R2CO2H = 34:4)		
			Ac1PIM1 (R1CO2H + R2CO2H + R3CO2H = 48:3)		
			PIM2 (R1CO2H + R2CO2H = 37:0)		
Exclusive lipid signatures in CWL extract					
FA	GL		GPL		PK
Mycocerosic acid (C30)	MG (RCO2H = 17:1)		Lyso‐PG (RCO2H = 17:0)		MPM (C31)
Mycocerosic acid (C33)	MG (RCO2H = 27:0)		Lyso‐PE (R1CO2H = 19:0)		MPM (C31)/PE (R1CO2H + R2CO2H = 33:2)
Phthioceranic acid (C33)	MG (RCO2H = 27:0)/PE (R1CO2H + R2CO2H = 33:2)		PE (R1CO2H + R2CO2H = 33:2)		
Mycocerosic acid (C34)	DG (R1CO2H + R2CO2H = 30:0)		Lyso‐PG (RCO2H = 17:0)/PE (R1CO2H + R2CO2H = 33:2)		
Phthioceranic acid (C34)	DG (R1CO2H + R2CO2H = 39:1)		Lyso‐PE (R1CO2H = 18:0)		
Mycolipanolic acid (C28)	DG (R1CO2H + R2CO2H = 33:1)				
Hydroxyphthioceranic acid (C48)	DG (R1CO2H + R2CO2H = 43:1)				
Mycocerosic acid (C27)	DG (R1CO2H + R2CO2H = 34:1)				
Mycolipenic acid (C28)	DG (R1CO2H + R2CO2H = 32:1)				
Mycocerosic acid (C34)/PE (R1CO2H + R2CO2H = 33:2)	DG (R1CO2H + R2CO2H = 35:1)				
Phthioceranic acid (C34)/PE (R1CO2H + R2CO2H = 33:2)	DG (R1CO2H + R2CO2H = 43:1)/PE (R1CO2H + R2CO2H = 33:2)				
Mycolipanolic acid (C28)/PE (R1CO2H + R2CO2H = 33:2)	DG (R1CO2H + R2CO2H = 32:1)/PE (R1CO2H + R2CO2H = 33:2)				
Mycolipenic acid (C28)/PE (R1CO2H + R2CO2H = 33:2)	DG (R1CO2H + R2CO2H = 30:1)				
**(B) Targeted MRM based**					
**Common lipid signatures in TL and CWL extracts**		**Exclusive lipid signatures in TL extract**		**Exclusive lipid signatures in CWL extract**	
GL	GPL	GL	GPL	GL	GPL
DG 12:0–16:0	LPC 14:0	TG (42:0/FA16:0)	PC 24:0	DG 16:1–18:4	LPC 16:0
DG 12:0–20:0	PE 32:0	TG (44:0/FA14:0)	PC 30:0	DG 20:0–22:0	LPC 18:0
DG 12:0–22:0	PE 33:1	TG (44:0/FA16:0)	PC 32:0		
DG 14:0–20:0	PE 34:0	TG (44:0/FA18:0)	PC 33:0		
DG 14:0–22:6	PE 35:1	TG (46:0/FA14:0)	PC 34:0		
DG 16:0–16:0	PE 37:2	TG (46:0/FA16:0)	PC 34:1		
DG 16:0–18:0		TG (46:0/FA18:0)	PC 35:1		
DG 16:1–16:1		TG (47:1/FA14:0)	PC 36:0		
DG 20:0–20:5		TG (47:1/FA16:1)	PC 36:1		
		TG (48:0/FA14:0)	PC 38:1		
		TG (48:0/FA16:0)	PC 40:1		
		TG (48:0/FA18:0)	LPC 17:0		
		TG(49:2/FA16:0)	LPC 20:5		
		TG(49:2/FA17:0)	LPC 22:0		
		TG(49:2/FA18:1)	PE 36:1		
		TG(50:0/FA14:0)	PE 38:1		
		TG(50:0/FA16:0)	PI_26:0		
		TG(50:0/FA18:0)	PI_34:2		
		TG(51:0/FA17:0)	PI_35:0		
		TG(51:0/FA18:0)	PI_35:2		
		TG(51:3/FA16:1)	PI_38:2		
		TG(51:3/FA17:0)	PS_32:0		
		TG(52:0/FA16:0)	PS_32:1		
		TG(52:0/FA18:0)	PS_34:1		
		TG(52:0/FA20:0)	PS_35:1		
		TG(55:7/FA22:6)	PS_35:2		
		DG 12:0‐12:0	PS_36:1		
		DG 12:0‐14:0			
		DG 12:0‐14:1			
		DG 12:0‐18:4			
		DG 12:0‐22:6			
		DG 14:0‐14:0			
		DG 14:0‐14:1			
		DG 14:0‐16:0			
		DG 14:0‐18:0			
		DG 14:0‐22:0			
		DG 14:1‐18:0			
		DG 14:1‐20:0			
		DG 14:1‐22:6			
		DG 16:0‐20:0			
		DG 16:0‐20:5			
		DG 16:0‐22:6			
		DG 16:1‐18:0			
		DG 16:1‐20:5			
		DG 16:1‐22:0			
		DG 16:1‐22:6			
		DG 18:0‐18:0			
		DG 18:0‐22:6			
		DG 18:1‐18:1			
		DG 18:1‐20:0			
		DG 18:1‐22:0			
		DG 18:1‐22:6			
		DG 18:2‐18:2			
		DG 18:2‐22:6			
		DG 18:3‐20:5			
		DG 18:3‐22:6			
		DG 20:0‐22:6			
		DG 22:6‐22:6			

## 4. Discussion

Differentiating MTB from NTM remains a critical clinical challenge due to overlapping clinical manifestations and limitations in current diagnostic tools [5, 16]. In this study, *M. kansasii* isolates, a clinically significant NTM species due to their close phenotypic and genomic resemblance to MTB and its emerging prevalence in TB‐endemic regions [4, 6] were studied. The comparative lipidomic profiling with MTB isolates using two distinct extraction methods: TL and CWL. TL extraction captures structural and storage lipids, whereas CWL extraction targets cell envelope‐associated lipids, including virulence factors essential for mycobacterial survival and pathogenicity [8, 17]. Molecular‐level characterization through dual extraction coupled with UHP‐LC MS/MS revealed distinct lipid compositions, leading to the identification of lipid‐metabolite signature panels that highlight key alterations in MTB and *M. kansasii*, offering valuable insights into their lipid biology [7].

Untargeted lipidomics using IDA in both positive and negative ion modes has proven effective for lipid profiling of MTB and *M. kansasii* [18]. Here, the data from both ionization modes were combined for comprehensive lipidomics analysis of the mycobacterial lipidome (Figure S1). Previous studies have reported several shared features between these two species, including the virulence locus (ESX‐1) and antigens (CFP‐10, ESAT‐6), as well as essential metabolic genes [6, 19, 20]. Consistently, our biostatistical analysis of TL extracts revealed moderate similarity in lipid classes distribution among MTB and *M. kansasii* (Figures 1 and 3a; Tables S1a and S2a; S. Sheet 1), suggesting comparable membrane architectures that may influence host–pathogen interactions and antimicrobial susceptibility.

Both MTB and *M. kansasii* comprise some common alpha and keto mycolates with varying chain length and abundance, reflecting a fundamental evolutionary divergence in pathogenic potential. MTB has specific long‐chain mycolates that enhance cell wall hydrophobicity, promoting immune evasion and intracellular persistence [6, 21, 22]. Whereas *M. kansasii* produces unique, shorter, branched, cis, and nonketo mycolates, indicative of its environmental adaptation and relatively reduced virulence [6, 22]. These compositional differences extend to associated lipid families: MTB mycolates are coupled with sulfoglycolipids and phthioceranate‐derived lipids that promote aerosol stability and transmission, whereas *M. kansasii* retains more hydrophilic lipooligosaccharides and phenolic glycolipids, maintaining a cell envelope better suited for environmental survival than for host infection [21, 23].

However, the detailed CWL data further demonstrated clear species‐specific distinctions (Figures 2 and 3b; Tables S1b and S2b; S. Sheet 2), consistent with earlier lipidomic and genomic reports [6, 9, 21]. MTB isolates showed enrichment in GLs, GPLs, and FAs, supporting intracellular survival and pathogenicity [13, 17], 24]. Conversely, *M. kansasii* displayed a broader lipid diversity dominated by PKs, PRs, and SLs, consistent with its aquatic origin and opportunistic ecological strategy. The predominance of PK‐derived stress‐response lipids reflects retention of diverse *pks* gene clusters that promote environmental resilience and metabolic flexibility rather than specialized virulence [6, 25–27]. Importantly, the evolutionary preservation of these PKS pathways enables *M. kansasii* to synthesize lipid‐derived secondary metabolites that enhance stress tolerance and ecological persistence, distinguishing it from obligate pathogenic mycobacteria such as MTB, which has streamlined its lipid metabolism toward host‐dependent pathogenicity and virulence optimization [6, 27]. Since lipidomic data are complex and extensive, a Venn diagram (Figure 4 S. Sheet 3) was used to compare and illustrate the shared and unique lipids with each type of lipid extract of MTB and *M. kansasii* isolates. This visualization simplified the untargeted datasets, providing a clear overview of relationships between lipid sets and facilitating data interpretation [28]. A total of 116 lipids (14.9%) are common across all four datasets, indicating a conserved lipidome core essential for mycobacterial viability regardless of extraction type [6]. However, distinct lipid subsets defined the ecological and pathogenic divergence between the two species. These compositional distinctions in lipid profiles may influence pathogenic potential, immune evasion, and antibiotic response, providing targets for species‐specific therapeutic interventions and foundation for species‐level differentiation [8, 17].

The MRM‐based targeted UHPLC–MS/MS approach enabled precise and high‐throughput quantification of mycobacterial lipid species within complex biological matrices by utilizing stable isotope‐labeled and natural standards [29, 30]. Optimization of LC‐MS parameters using the SPLASH lipidomics mix and natural standards for TG, DG, PC, LPC, PE, PI, and PS (Table S3; Figure S2) ensured accurate quantification across lipid subclasses. The strong linearity of calibration curves (*R* ^2^ = 0.99) and validated LOD/LOQ values (Table S4) confirmed high analytical precision and reproducibility, strengthening the reliability of observed lipid variations between MTB and *M. kansasii*.

Targeted quantification of GL and GPL subclasses validated the differential lipidomic patterns identified through untargeted profiling, reinforcing the species‐specific lipid distinctions. Integrating untargeted discovery with targeted validation enhanced the molecular coverage, reliability, and biological relevance of our results, confirming that observed lipid variations represent true compositional differences rather than analytical artifacts (Sa et al. 2024; [12, 31, 32]). Quantification based on consistently detected mycobacteria‐specific transition pairs provided a robust foundation for defining lipid signatures associated with virulence and metabolic adaptation, offering deeper mechanistic insights into the functional lipid architecture of MTB and *M. kansasii* (Table S5).

The targeted lipidomic analysis of GLs revealed higher concentrations of mostly TG and DG species in MTB compared with *M. kansasii* (Tables 1 and 2; Figures 5, S3, and S4). In MTB, TG enrichment and predominance of long‐chain species such as TG (51:3/FA 18:2) and TG (55:7/FA 22:6) indicate metabolic adaptation for intracellular survival and dormancy. The *Tgs1*‐mediated biosynthetic pathway promotes TG accumulation in intracellular lipid inclusions (ILIs) under hypoxia or nutrient deprivation, supporting dormancy, antibiotic tolerance, and long‐term survival in the host, consistent with previous studies [5, 33–35].

Conversely, *M. kansasii* showed a broader but lower abundance TG profile, with short‐chain species such as TG (42:0/FA 14:0) and TG (42:0/FA 16:0) being relatively enriched. This compositional diversity likely reflects its metabolic flexibility and adaptation to fluctuating environmental conditions rather than host‐dependent persistence, consistent with its opportunistic lifestyle and reduced dependence on lipid‐based energy storage [6].

Likewise, elevated levels of DGs in MTB may link to active lipid flux through the diacylglycerol acyltransferase (DGAT/Tgs) pathway, which acylates DG to form TG [36]. Under stress, Tgs1 and Tgs2 enzymes drive TG synthesis, whereas LipY lipase mediates TG hydrolysis back to DG, maintaining energy balance and supporting cell wall maintenance during reactivation [17, 33, 36]. This bidirectional TG–DG turnover reflects a dynamic lipid recycling mechanism that underpins metabolic plasticity and virulence adaptation in MTB.

In contrast, *M. kansasii* exhibited a more balanced DG profile, with species such as DG (18:1–18:4) enriched, suggesting less reliance on the DGAT‐mediated storage pathway and greater lipid compositional flexibility for environmental adaptation. Collectively, these findings suggest that MTB′s DG–TG metabolic axis is tightly regulated through DGAT and LipY activity to sustain intracellular survival and virulence, whereas *M. kansasii* maintains a metabolically adaptable but less virulence‐oriented lipid network [13, 33, 36].

GPLs, including PC, LPC, PE, PI, and PS, are crucial for mycobacterial membrane integrity, fluidity, and immune modulation [13, 35]. Here, GPL profiling revealed distinct species‐specific patterns between MTB and *M. kansasii* (Figures 5, S3b, and S4b; Tables 1 and 2), reflecting their divergent physiological and pathogenic adaptations. In TL extracts, MTB exhibited higher concentrations of PCs (notably PC 34:0), LPCs (specifically, LPC 14:0), PEs (PEs 32:7 and 38:1), and PI species that collectively reinforce membrane stability and intracellular persistence [37, 38]. PI serves as precursors for PIMs, LM, and LAM, which regulate phagosome maturation and attenuate proinflammatory immune responses through pattern recognition receptor signaling [5]. PE‐containing lipids contribute to the structural integrity of the mycobacterial envelope and facilitate nutrient assimilation within foamy macrophages [39], whereas PS enrichment suggests apoptotic mimicry contributing to immune evasion [40]. LPC, derived from PC hydrolysis, exhibits paradoxical activity: exogenous LPC can promote bactericidal macrophage signaling through the G2A–PKA–PI3K–p38 MAPK axis, yet MTB suppresses endogenous LPC accumulation to avoid this host defense mechanism [41]. Thus, the elevated levels of these phospholipids in MTB represent a strategically regulated lipid composition that balances membrane functionality with host immune manipulation to sustain chronic infection.

Conversely, *M. kansasii* showed relatively elevated levels of PC 24:0, LPC 17:0, and PI 31:2, particularly in CWL extracts, indicating adaptive membrane organization optimized for environmental stress endurance rather than intracellular residence [6]. PS was absent in CWL extracts of both MTB and *M. kansasii*, consistent with its localization to the cytoplasmic membrane rather than the cell wall, as reported previously [8, 21, 40].

Furthermore, the ROC curve was utilized to identify and validate discriminant potential lipid biomarkers derived from UHPLC–MS/MS datasets of MTB and *M. kansasii* isolates. The ROC framework enabled quantitative evaluation of biomarker performance based on sensitivity, specificity, and a high AUC, providing a robust measure of diagnostic accuracy [42–44]. The ROC framework enabled quantitative evaluation of biomarker performance based on sensitivity, specificity, and the AUC, providing a robust measure of diagnostic accuracy [42–44]. Using MetaboAnalyst 6.0, ROC modeling achieved an AUC of 1.0 (95% *C* *I* = 1.0, 1 sensitivity, 1 specificity) across both TL and CWL datasets, reflecting complete discrimination between species. Although a perfect AUC may suggest potential model inflation, rigorous PLS‐DA cross‐validation (*R* ^2^ = 0.90, *Q* ^2^ = 0.78 for TL; *R* ^2^ = 0.83, *Q* ^2^ = 0.76 for CWL) confirmed the robustness and predictive reliability of the models, excluding overfitting. Thus, the perfect AUC values reflect genuine lipidomic separation rather than overfitting, further supported by tenfold cross‐validation.

The preliminary biomarker panels derived from untargeted datasets, comprised 36 and 38 discriminant lipid species found in the bacilli TL and CWL extracts, respectively (Table 3a). These represent statistically significant differentiators between MTB and *M. kansasii*, serving as a discovery‐level dataset rather than a finalized diagnostic panel. TL profiles were enriched in FA, GL, and GPL classes, whereas CWL additionally featured PK lipids, reflecting the distinct metabolic architectures and cell envelope specializations in the two species. After exhaustive multivariate and univariate analyses, five lipid species comprising one FA, three GLs, and one GPL from these panels consistently showed strong discrimination between MTB and *M. kansasii* and were identified in both TL and CWL extracts (Figure 6; Table 3a). Additionally, differential expression analysis (*p* ≤ 0.05) of targeted datasets revealed up‐ and down‐regulated GL and GPL species (Figure 7; Table 3b), which emerged as biologically meaningful discriminators further supporting their diagnostic potential. These lipids are mechanistically linked to membrane signaling, host immune modulation, and intracellular persistence [5, 13, 38]. In contrast, the predominance of MG and PK lipids in *M. kansasii* reflects metabolic versatility and environmental adaptation rather than virulence specialization [6]. Thus, rather than proposing all 36–38 lipids as biomarkers, this study identifies and highlights a mechanistically defined subset of functionally relevant lipid species that differentiate MTB and *M. kansasii* at the species level and hold potential for diagnostic refinement.

However, this study was limited by the small number of clinical isolates analyzed, owing to BSL‐3 facility constraints and restricted patient sample availability, which may not fully capture intraspecies heterogeneity. Although the lipidomic findings were statistically validated and demonstrated strong discriminatory potential, further evaluation using larger, geographically diverse cohorts and functional assays is required to confirm the diagnostic and mechanistic relevance of the proposed biomarkers. Additionally, integrating host–pathogen lipid interaction studies would further strengthen the translational applicability of these findings.

Collectively, this integrated untargeted and targeted UHPLC–MS/MS analysis establishes that MTB and *M. kansasii* possess distinct lipidomic blueprints reflecting their ecological and pathogenic specialization and strategies. MTB′s enrichment in long‐chain TGs, DGs, and immunomodulatory GPLs underlines its lipid‐dependent adaptation for intracellular persistence and immune evasion, whereas *M. kansasii* retains a metabolically versatile but less virulent lipid repertoire dominated by short‐chain TGs and PKs. These mechanistically defined lipid signatures not only provide new insights into mycobacterial physiology but also form a basis for developing species‐specific lipid biomarkers and diagnostic tools for improved clinical differentiation of MTB and *M. kansasii* infections.

## 5. Conclusion

**Figure 8:**
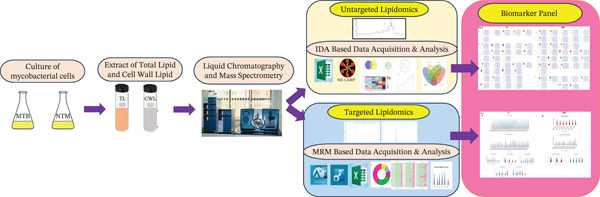
Schematic workflow of integrated lipidomic workflow and potential biomarker panel for MTB and *M. kansasii* differentiation. The schematic summarizes the experimental design integrating untargeted and targeted LC–MS/MS workflows, multivariate analysis, and ROC validation to identify species‐specific lipid signatures distinguishing MTB from *M. kansasii*.

MTB and *M. kansasii* comprise complex and unique lipid profiles. Through advanced lipidomic techniques, we were able to identify specific lipid species that are either unique or common between MTB and *M. kansasii.* Our comparative analysis of the lipidome profiles of MTB and *M. kansasii* using TL and CWL extractions revealed distinct lipid compositions at the molecular level. Untargeted lipidomics via IDA in both positive and negative ion modes allowed a comprehensive analysis of lipid profiles, aiding in identifying new biomarkers and understanding lipid diversity and metabolism in these pathogens (Figure 8). Targeted lipidomics or MRM‐based methods enabled absolute quantification of specific molecular analytes, revealing significant changes in two major lipid categories, that is, GL and GPL. The absolute quantification of GL and GPL molecular species from both TL and CWL extracts of MTB and *M. kansasii* clinical isolates provided further insights into their differential distribution and abundance. Additionally, the biomarker analysis revealed distinct lipid signatures that can aid in distinguishing MTB from *M. kansasii* and are a significant addition for developing novel lipid‐based diagnostic biomarkers and therapeutic targets.

## Author Contributions

Meenakshi Chugh: data curation, formal analysis, investigation, methodology, validation, visualization, and roles/writing—original draft. Saif Hameed: conceptualization, project administration, resources, supervision, validation, and writing—review and editing. Jitendra Singh: resources, supervision, validation, and writing—review and editing. Zeeshan Fatima: conceptualization, formal analysis, investigation, methodology, project administration, resources, software, supervision, validation, visualization, roles/writing—original draft, and writing—review and editing.

## Funding

This study was supported by the Indian Council of Medical Research (5/8/5/13/ITRC/Diag/2022/ECD‐1) and the Department of Biotechnology, Ministry of Science and Technology, India (BT/PR/23016/NER/95/2017).

## Ethics Statement

Clinical sputum samples for MTB and *M. kansasii* were obtained under approval from the Institutional Ethics Committee of AIIMS, Bhopal (Approval No.: IHEC‐LOP/2018/EF0104) which is a part of a multiconsortium grant where the project coordinator had no objections. Written informed consent was obtained from each patient prior to the sample collection, in accordance with the study′s inclusion criteria and institutional ethical guidelines. All subsequent lipid extraction and LC–MS/MS‐based lipidomic analyses were performed at Amity University Haryana, Gurgaon, following approval from its institutional ethics committee (Approval No.: IEC‐AIB/AUH/2018‐3).

## Conflicts of Interest

The authors declare no conflicts of interest.

## Supporting Information

Additional supporting information can be found online in the Supporting Information section.

## Supporting information


**Supporting Information 1** Table S1: Overall and category‐wise minimum and maximum queried and identified m/z values in TL and CWL extracts. Table S2: Number of lipid subclasses for each lipid class among MTB and NTM isolates. Table S3: Optimized MRM transitions and mass spectrometry parameters for each GL and GPL species. Table S4: Details of the calibration curves of internal and natural GL and GPL standards, including LOD and LOQ. Table S5: Optimized transition pairs including precursor ion (Q1), product ion (Q3), and respective IDs. Table S6: Absolute concentrations for all quantified lipid species from GL and GPL categories. Figure S1: IDA chromatograms of MTB and NTM isolates in pos and neg ionization mode. Figure S2: Q1 scan, MS2 scan, and XICs of TG, DG, PC, LPC, PE, PI, and PS, TG natural standards for quantitative analysis. Figure S3: Distribution percentages of quantified lipid species of TG, DG, PC, LPC, PE, PI, and PS. Figure S4: Relative distribution of quantified lipid species of TG, DG, PC, LPC, PE, PI, and PS in percentage among MTB and NTM isolates.

**Supporting Information 2** Excel Sheet: S. Sheet 1: List of exclusive and common lipids in Figure 1c,e. S. Sheet 2: List of exclusive and common lipids in Figure 2c,e. S. Sheet 3: List of exclusive and common lipids in Figure 4.

## Data Availability

The data that support the findings of this study are available on request from the corresponding authors. The data are not publicly available due to privacy or ethical restrictions.

## References

[1] Global Programme on Tuberculosis and Lung Health (GTB) , October 29, Global Tuberculosis Report. (2024) 2024, https://www.who.int/publications/i/item/9789240101531.

[2] Anh N. K. , Phat N. K. , Thu N. Q. , Tien N. T. N. , Eunsu C. , Kim H.-S. , Nguyen D. N. , Kim D. H. , Long N. P. , and Oh J. Y. , Discovery of Urinary Biosignatures for Tuberculosis and Nontuberculous Mycobacteria Classification Using Metabolomics and Machine Learning, Scientific Reports. (2024) 14, no. 1, 10.1038/s41598-024-66113-x, 38961191. PMC11222504 38961191

[3] Sousa S. , Bandeira M. , Carvalho P. A. , Duarte A. , and Jordao L. , Nontuberculous Mycobacteria Pathogenesis and Biofilm Assembly, International Journal of Mycobacteriology. (2015) 4, no. 1, 36–43, 10.1016/j.ijmyco.2014.11.065. 26655196

[4] Guo Y. , Cao Y. , Liu H. , Yang J. , Wang W. , Wang B. , Li M. , and Yu F. , Lamichhane G. , Clinical and Microbiological Characteristics of *Mycobacterium kansasii* Pulmonary Infections in China, Microbiology Spectrum. (2022) 10, no. 1, 10.1128/spectrum.01475-21, 35019778. PMC8754148 35019778

[5] Tran T. , Bonham A. J. , Chan E. D. , and Honda J. R. , A Paucity of Knowledge Regarding Nontuberculous Mycobacterial Lipids Compared to the Tubercle Bacillus, Tuberculosis. (2019) 115, 96–107, 10.1016/j.tube.2019.02.008. 30948183

[6] Wang J. , Rocha E. P. , Mcintosh F. , Veyrier F. J. , Brosch R. , Simeone R. , Behr M. A. , Dewar K. , Radomski N. , and Enninga J. , Insights on the Emergence of *Mycobacterium tuberculosis* From the Analysis of *Mycobacterium kansasii* , Genome Biology and Evolution. (2015) 7, no. 3, 856–870, 10.1093/gbe/evv035, 25716827. 25716827 PMC5322544

[7] Gonzalo X. , Broda A. , Drobniewski F. , and Larrouy-Maumus G. , Performance of Lipid Fingerprint-Based MALDI-ToF for the Diagnosis of Mycobacterial Infections, Clinical Microbiology and Infection. (2021) 27, no. 6, 912.e1–912.e5, 10.1016/j.cmi.2020.08.027, 32861860. PMC8186428 32861860

[8] Sartain M. J. , Dick D. L. , Rithner C. D. , Crick D. C. , and Belisle J. T. , Lipidomic Analyses of *Mycobacterium tuberculosis* Based on Accurate Mass Measurements and the Novel “Mtb LipidDB.”, Journal of Lipid Research. (2011) 52, no. 5, 861–872, 10.1194/jlr.m010363. 21285232 PMC3073466

[9] Layre E. , Sweet L. , Hong S. , Madigan C. A. , Desjardins D. , Young D. C. , Cheng T.-Y. , Annand J. W. , Kim K. , Shamputa I. C. , McConnell M. J. , Debono C. A. , Behar S. M. , Minnaard A. J. , Murray M. , Barry C. E. , Matsunaga I. , and Moody D. B. , A Comparative Lipidomics Platform for Chemotaxonomic Analysis of Mycobacterium tuberculosis.”, Chemistry & Biology. (2011) 18, no. 12, 10.1016/j.chembiol.2011.10.013, 22195556. PMC3407843 22195556

[10] Layre E. , Lee H. J. , Young D. C. , Jezek Martinot A. , Buter J. , Minnaard A. J. , Annand J. W. , Fortune S. M. , Snider B. B. , Matsunaga I. , Rubin E. J. , Alber T. , and Moody D. B. , Molecular Profiling of *Mycobacterium tuberculosis* Identifies Tuberculosinyl Nucleoside Products of the Virulence-Associated Enzyme Rv3378c, Proceedings of the National Academy of Sciences. (2014) 111, no. 8, 2978–2983, 10.1073/pnas.1315883111, 24516143. PMC3939896 24516143

[11] Gonzalo X. , Yrah S. , Broda A. , Laurenson I. , Claxton P. , Kostrzewa M. , Drobniewski F. , and Larrouy-Maumus G. , Performance of Lipid Fingerprint by Routine Matrix-Assisted Laser Desorption/Ionization Time of Flight for the Diagnosis of *Mycobacterium tuberculosis* Complex Species, Clinical Microbiology and Infection. (2023) 29, no. 3, 387.e1–387.e6, 10.1016/j.cmi.2022.10.017. 36270589

[12] Wang L. , Yang G. , Guo L. , Yao L. , Liu Y. , and Sha W. , Olink Proteomics and Lipidomics Analysis of Serum From Patients Infected With Non-Tuberculous Mycobacteria and *Mycobacterium tuberculosis* , Inflammation Research. (2024) 73, no. 11, 1945–1960, 10.1007/s00011-024-01943-z, 39340659. 39340659 PMC11541342

[13] Pal R. , Hameed S. , Kumar P. , Singh S. , and Fatima Z. , Comparative Lipidomics of Drug-Sensitive and Resistant *Mycobacterium tuberculosis* Reveals Altered Lipid Imprints, In 3 Biotech. (2017) 7, no. 5, 10.1007/s13205-017-0972-6, 28955622. PMC5602786 28955622

[14] Fatima Z. , Chugh M. , Nigam G. , and Hameed S. , Quantification of Mycolic Acids in Different Mycobacterial Species by Standard Addition Method Through Liquid Chromatography Mass Spectrometry, Journal of Chromatography B. (2024) 1247, 124297, 10.1016/j.jchromb.2024.124297, 39299149. 39299149

[15] Pal R. , Hameed S. , Sabareesh V. , Kumar P. , Singh S. , and Fatima Z. , Investigations Into Isoniazid Treated *Mycobacterium tuberculosis* by Electrospray Mass Spectrometry Reveals New Insights Into Its Lipid Composition, Journal of Pathogens. (2018) 2018, 10.1155/2018/1454316, 1454316. 30018826 PMC6029481

[16] Sinha P. , Gupta A. , Prakash P. , Anupurba S. , Tripathi R. , and Srivastava G. N. , Differentiation of *Mycobacterium tuberculosis* Complex From Non-Tubercular Mycobacteria by Nested Multiplex PCR Targeting IS6110, MTP40 and 32kD Alpha Antigen Encoding Gene Fragments, BMC Infectious Diseases. (2016) 16, 123, 10.1186/s12879-016-1450-1, 26968508. 26968508 PMC4788904

[17] Ghazaei C. , *Mycobacterium tuberculosis* and Lipids: Insights Into Molecular Mechanisms From Persistence to Virulence, Journal of Research in Medical Sciences. (2018) 23, no. 1, 10.4103/jrms.jrms_904_17. PMC6091133 30181745

[18] Breitkopf S. B. , Ricoult S. J. H. , Yuan M. , Xu Y. , Peake D. A. , Manning B. D. , and Asara J. M. , A Relative Quantitative Positive/Negative Ion Switching Method for Untargeted Lipidomics via High-Resolution LC-MS/MS From any Biological Source, Metabolomics. (2017) 13, no. 3, 10.1007/s11306-016-1157-8, 28496395. PMC5421409 28496395

[19] Johnston J. C. , Elwood K. , and Chiang L. , Mycobacterium kansasii , Spectrum. (2017) 5, no. 1, 10.1128/microbiolspec.tnmi7-0011-2016, 28185617. PMC11687434 28185617

[20] Levendosky K. , Janisch N. , and Quadri L. E. N. , Comprehensive Essentiality Analysis of the *Mycobacterium kansasii* Genome by Saturation Transposon Mutagenesis and Deep Sequencing, MBio. (2023) 14, no. 4, 10.1128/mbio.00573-23, 37350613. PMC10470612 37350613

[21] Jankute M. , Nataraj V. , Lee O. Y.-C. , Wu H. H. T. , Ridell M. , Garton N. J. , Barer M. R. , Minnikin D. E. , Bhatt A. , and Besra G. S. , The Role of Hydrophobicity in Tuberculosis Evolution and Pathogenicity, Scientific Reports. (2017) 7, no. 1, 10.1038/s41598-017-01501-0, 28465507. PMC5431016 28465507

[22] Kaneda K. , Naito S. , Imaizumi S. , Yano I. , Mizuno S. , Tomiyasu I. , Baba T. , Kusunose E. , and Kusunose M. , Determination of Molecular Species Composition of C80 or Longer-Chain alpha-mycolic Acids in *Mycobacterium* spp. by Gas Chromatography-Mass Spectrometry and Mass Chromatography, Journal of Clinical Microbiology. (1986) 24, no. 6, 1060–1070, 10.1128/JCM.24.6.1060-1070.1986, 3782454. 3782454 PMC269099

[23] Rafał S. , Magdalena D. , Karol M. , Bartłomiej S. , and Konrad K. , Detection and Accurate Identification of *Mycobacterium* species by flow Injection Tandem Mass Spectrometry (FIA-MS/MS) Analysis of Mycolic Acids, Scientific Reports. (2025) 15, no. 1, 10.1038/s41598-025-96867-x, 40240395. PMC12003690 40240395

[24] Holzheimer M. , Buter J. , and Minnaard A. J. , Chemical Synthesis of Cell Wall Constituents of *Mycobacterium tuberculosis* , Chemical Reviews. (2021) 121, no. 15, 9554–9643, 10.1021/acs.chemrev.1c00043, 34190544. 34190544 PMC8361437

[25] Luo T. , Xu P. , Zhang Y. , Porter J. L. , Ghanem M. , Liu Q. , Jiang Y. , Li J. , Miao Q. , Hu B. , Howden B. P. , Fyfe J. A. M. , Globan M. , He W. , He P. , Wang Y. , Liu H. , Takiff H. E. , Zhao Y. , Chen X. , Pan Q. , Behr M. A. , Stinear T. P. , and Gao Q. , Population Genomics Provides Insights Into the Evolution and Adaptation to Humans of the Waterborne Pathogen *Mycobacterium* kansasii, Communications. (2021) 12, no. 1, 10.1038/s41467-021-22760-6, 33941780. PMC8093194 33941780

[26] Pereira A. C. , Ramos B. , Reis A. C. , and Cunha M. V. , Non-Tuberculous Mycobacteria: Molecular and Physiological Bases of Virulence and Adaptation to Ecological Niches, Microorganisms. (2020) 8, no. 9, 10.3390/microorganisms8091380, 32916931. PMC7563442 32916931

[27] Sarkar B. , Srivastava S. , and Gokhale R. S. , Polyketide Synthases in Mycobacterial Lipid Metabolism, Biology of Mycobacterial Lipids, 2022, Elsevier, 207–220, 10.1016/b978-0-323-91948-7.00006-3.

[28] Hewelt-Belka W. and Kot-Wasik A. , Analytical Strategies and Applications in Lipidomics, Handbook of Bioanalytics, 2022, Springer International Publishing, 1–26, 10.1007/978-3-030-63957-0_7-1.

[29] Cox D. and Lee H. , Scout Triggered MRM Algorithm: The Evolution of the MRM Workflow, 2022, SCIEX, https://sciex.com/content/dam/SCIEX/tech-notes/technology/ruo-mkt-01-14731-a/Scout-triggered-MRM_Technology__RUO-MKT-02-14731-A.pdf.

[30] Gutierrez Reyes C. D. , Sanni A. , Adeniyi M. , Mogut D. , Najera Gonzalez H. R. , Ahmadi P. , Atashi M. , Onigbinde S. , and Mechref Y. , Targeted Glycoproteomics Analysis Using MRM/PRM Approaches, Methods in Molecular Biology, 2024, Springer US, 231–250, 10.1007/978-1-0716-3666-4_14. PMC11773419 38315369

[31] Sa Y. , Ding S. , Zhang Y. , Wang W. , Wilson G. , Ma F. , Zhang W. , and Ma X. , Integrating untargeted and targeted LC–MS-based metabolomics to identify the serum metabolite biomarkers for tuberculosis, Biomedical Chromatography. (2024) 38, no. 11, e5998, 10.1002/bmc.5998, 39193838. 39193838

[32] Sun B. , Liu F. , Yin Q. , Jiang T. , Fang M. , Duan L. , Quan S. , Tian X. , Shen A. , Mi K. , and Sun L. , Exploration of Lipid Metabolism Alterations in Children With Active Tuberculosis Using UHPLC-MS/MS, Journal of Immunology Research. (2023) 2023, 10.1155/2023/8111355, 8111355. 36815950 PMC9936505

[33] Daniel J. , Maamar H. , Deb C. , Sirakova T. D. , and Kolattukudy P. E. , *Mycobacterium tuberculosis* Uses Host Triacylglycerol to Accumulate Lipid Droplets and Acquires a Dormancy-Like Phenotype in Lipid-Loaded Macrophages, PLoS Pathogens. (2011) 7, no. 6, 10.1371/journal.ppat.1002093, 21731490. PMC3121879 21731490

[34] Kim H. and Shin S. J. , Revolutionizing Control Strategies Against *Mycobacterium tuberculosis* Infection Through Selected Targeting of Lipid Metabolism, Cellular and Molecular Life Sciences. (2023) 80, no. 10, 10.1007/s00018-023-04914-5, 37704889. PMC11072447 37704889

[35] Mallick I. , Santucci P. , Poncin I. , Point V. , Kremer L. , Cavalier J.-F. , and Canaan S. , Intrabacterial Lipid Inclusions in Mycobacteria: Unexpected Key Players in Survival and Pathogenesis?, FEMS Microbiology Reviews. (2021) 45, no. 6, 10.1093/femsre/fuab029, 34036305. 34036305

[36] Crotta Asis A. , Savoretti F. , Cabruja M. , Gramajo H. , and Gago G. , Characterization of Key Enzymes Involved in Triacylglycerol Biosynthesis in Mycobacteria, Scientific Reports. (2021) 11, no. 1, 10.1038/s41598-021-92721-y, 34168231. PMC8225852 34168231

[37] Pearson M. , Kapil S. K. , Norris P. , and Hunter C. , Achieve Broad Lipid Quantitation Using a high-Throughput Targeted Lipidomics Method, 2020, SCIEX, https://sciex.jp/content/dam/SCIEX/pdf/tech-notes/all/Achieve-Broad-Lipid-Quantitation-using-a-High-Throughput-Targeted-Lipidomics-Method.pdf.

[38] Wallner S. , Orsó E. , Grandl M. , Konovalova T. , Liebisch G. , and Schmitz G. , Phosphatidylcholine and Phosphatidylethanolamine Plasmalogens in lipid Loaded Human Macrophages, PLOS ONE. (2018) 13, no. 10, e0205706, 10.1371/journal.pone.020570. 30308051 PMC6181407

[39] Dargham T. , Mallick I. , Kremer L. , Santucci P. , and Canaan S. , Intrabacterial Lipid Inclusion-Associated Proteins: A Core Machinery Conserved From Saprophyte Actinobacteria to the Human Pathogen *Mycobacterium tuberculosis* , FEBS Open Bio. (2023) 13, no. 12, 2306–2323, 10.1002/2211-5463.13721, 37872001. PMC10699116 37872001

[40] Messias M. C. F. , Mecatti G. C. , Priolli D. G. , and de Oliveira Carvalho P. , Plasmalogen Lipids: Functional Mechanism and Their Involvement in Gastrointestinal Cancer, Lipids in Health and Disease. (2018) 17, no. 1, 10.1186/s12944-018-0685-9, 29514688. PMC5842581 29514688

[41] Lee H.-J. , Ko H.-J. , Song D.-K. , and Jung Y.-J. , Lysophosphatidylcholine Promotes Phagosome Maturation and Regulates Inflammatory Mediator Production Through the Protein Kinase A–Phosphatidylinositol 3 Kinase–p38 Mitogen-Activated Protein Kinase Signaling Pathway During *Mycobacterium tuberculosis* Infection in Mouse Macrophages, Frontiers in Immunology. (2018) 9, 10.3389/fimmu.2018.00920, 29755479. PMC5934435 29755479

[42] Franco-Pereira A. M. , Nakas C. T. , and Pardo M. C. , Biomarker Assessment in ROC Curve Analysis Using the Length of the Curve as an Index of Diagnostic Accuracy: The Binormal Model Framework, AStA Advances in Statistical Analysis. (2020) 104, no. 4, 625–647, 10.1007/s10182-020-00371-8.

[43] Han Y.-S. , Chen J.-X. , Li Z.-B. , Chen J. , Yi W.-J. , Huang H. , Wei L.-L. , Jiang T.-T. , and Li J.-C. , Identification of Potential Lipid Biomarkers for Active Pulmonary Tuberculosis Using Ultra-High-Performance Liquid Chromatography-Tandem Mass Spectrometry, Experimental Biology and Medicine. (2021) 246, no. 4, 387–399, 10.1177/1535370220968058, 33175608. 33175608 PMC7885049

[44] Hassanzad M. and Hajian-Tilaki K. , Methods of Determining Optimal Cut-Point of Diagnostic Biomarkers With the Application of Clinical Data in ROC Analysis: An Update Review, BMC Medical Research Methodology. (2024) 24, no. 1, 10.1186/s12874-024-02198-2, 38589814. PMC11000303 38589814

