# Supplementary material for: Untargeted and Targeted Liquid Chromatography‐Mass Spectrometry–Based Lipidomic Profiling Revealed a Potential Biomarker Panel to Distinguish Clinical Isolates of M. tuberculosis From Nontuberculous M. kansasii
Source: Pulm Med. 2026 May 31;2026:5535650. doi: 10.1155/pm/5535650 (PMC13239167; doi:10.1155/pm/5535650)
Supplement: Supplementary file 1 — Supporting Information 1 Table S1: Overall and category‐wise minimum and maximum queried and identified m/z values in TL and CWL extracts. Table S2: Number of lipid subclasses for each lipid class among MTB and NTM isolates. Table S3: Optimized MRM transitions and mass spectrometry parameters for each GL and GPL species. Table S4: Details of the calibration curves of internal and natural GL and GPL standards, including LOD and LOQ. Table S5: Optimized transition pairs including precursor ion (Q1), product ion (Q3), and respective IDs. Table S6: Absolute concentrations for all quantified lipid species from GL and GPL categories. Figure S1: IDA chromatograms of MTB and NTM isolates in pos and neg ionization mode. Figure S2: Q1 scan, MS2 scan, and XICs of TG, DG, PC, LPC, PE, PI, and PS, TG natural standards for quantitative analysis. Figure S3: Distribution percentages of quantified lipid species of TG, DG, PC, LPC, PE, PI, and PS. Figure S4: Relative distribution of quantified lipid species of TG, DG, PC, LPC, PE, PI, and PS in percentage among MTB and NTM isolates. [file PM-2026-5535650-s002.doc]

Table S1: Minimum and maximum queried and identified m/z values.

**(A) TL extracts**

|  | **m/z values** | **MTB 1** | **MTB 2** | **MTB 3** | ***M. kansasii* 1** | ***M. kansasii* 2** | ***M. kansasii* 3** |
| --- | --- | --- | --- | --- | --- | --- | --- |
| Queried | Minimum | 200.04 | 200.16 | 200.04 | 200.04 | 200.04 | 200.04 |
|  | Maximum | 1771.44 | 1780.08 | 1872.12 | 1640.88 | 1539.72 | 1545.72 |
| Identified | Minimum | 303.12 | 303.24 | 303.24 | 303.24 | 303.24 | 301.2 |
|  | Maximum | 1771.44 | 1566.84 | 1820.88 | 1638.84 | 1688.76 | 1545.72 |
|  | | | | | | | |
|  | **m/z values (Identified)** |  | | | | | |
|  | | | | | | | |
| **Fatty Acyls** | Minimum | 369.36 | 381.84 | 409.92 | 427.92 | 369.84 | 367.44 |
|  | Maximum | 1619.64 | 1565.4 | 1465.68 | 1617.48 | 1505.16 | 1545.72 |
|  | | | | | | | |
| **Glycerolipids** | Minimum | 303.12 | 303.24 | 303.24 | 303.24 | 303.24 | 301.2 |
|  | Maximum | 1299.36 | 1267.32 | 1228.92 | 1267.32 | 1215 | 1257.24 |
|  | | | | | | | |
| **Glycerophospholipids** | Minimum | 509.76 | 452.4 | 464.52 | 450.36 | 452.76 | 450.48 |
|  | Maximum | 1771.44 | 1566.84 | 1820.88 | 1638.84 | 1688.76 | 1422.96 |
|  | | | | | | | |
| **Polyketides** | Minimum | 737.88 | 859.08 | 681.72 | 873.36 | 826.8 | 695.76 |
|  | Maximum | 911.88 | 895.68 | 880.08 | 895.68 | 881.88 | 909.72 |
|  | | | | | | | |
| **Prenol Lipids** | Minimum | 700.08 | 00.00 | 786.24 | 698.16 | 698.4 | 00.00 |
|  | Maximum | 884.64 | 00.00 | 860.4 | 700.08 | 786.48 | 00.00 |
|  | | | | | | | |
| **Saccharolipids** | Minimum | 974.4 | 930.48 | 972.36 | 988.56 | 988.2 | 972.24 |
|  | Maximum | 1619.64 | 1112.4 | 972.36 | 1617.48 | 1364.88 | 976.44 |

**(B)** **CWL extract**

|  | **m/z values** | **MTB 1** | **MTB 2** | **MTB 3** | ***M. kansasii* 1** | ***M. kansasii* 2** | ***M. kansasii* 3** |
| --- | --- | --- | --- | --- | --- | --- | --- |
| Queried | Minimum | 200.04 | 200.04 | 200.04 | 200.04 | 200.04 | 200.16 |
|  | Maximum | 1647.24 | 1882.08 | 1679.76 | 1856.16 | 1688.76 | 1824.6 |
| Identified | Minimum | 301.68 | 303.24 | 303.24 | 303.24 | 303.24 | 301.2 |
|  | Maximum | 1647.24 | 1882.08 | 1673.16 | 1731.24 | 1688.76 | 1639.08 |
|  | | | | | | | |
|  | **m/z values (Identified)** |  | | | | | |
|  | | | | | | | |
| **Fatty Acyls** | Minimum | 383.88 | 381.48 | 383.52 | 385.8 | 369.84 | 383.52 |
|  | Maximum | 1601.76 | 1882.08 | 1509.48 | 1425.24 | 1375.2 | 1501.56 |
|  | | | | | | | |
| **Glycerolipids** | Minimum | 301.68 | 303.24 | 303.24 | 303.24 | 303.24 | 301.2 |
|  | Maximum | 1297.44 | 1241.04 | 1227.36 | 1100.64 | 1313.16 | 1084.92 |
|  | | | | | | | |
| **Glycerophospholipids** | Minimum | 480.84 | 452.4 | 450.72 | 450.48 | 450.48 | 469.32 |
|  | Maximum | 1647.24 | 1570.92 | 1673.16 | 1731.24 | 1688.76 | 1639.08 |
|  | | | | | | | |
| **Polyketides** | Minimum | 693.72 | 693.84 | 681.72 | 693.84 | 736.08 | 680.04 |
|  | Maximum | 882 | 895.92 | 896.04 | 909.96 | 912.12 | 924.12 |
|  | | | | | | | |
| **Prenol Lipids** | Minimum | 858.24 | 786.24 | 700.2 | 00.00 | 720.24 | 786.12 |
|  | Maximum | 858.36 | 884.4 | 700.32 | 00.00 | 884.52 | 882.36 |
|  | | | | | | | |
| **Saccharolipids** | Minimum | 1084.32 | 946.32 | 1096.44 | 948.12 | 1030.32 | 932.4 |
|  | Maximum | 1166.76 | 1293.12 | 1096.44 | 1236.6 | 1030.32 | 1222.68 |

Table S2: Number of lipid subclasses identified for each lipid category among MTB and *M. kansasii* isolates.

**(A) TL extract**

A1

| **Sub-class of Fatty Acyls** | **MTB** | ***M. kansasii*** |
| --- | --- | --- |
| Branched Fatty Acids | 33 | 27 |
| Keto Mycolic Acids (Keto-MA) | 2 | 1 |
| Methoxy Mycolic Acids (Methoxy-MA) | 4 | 1 |
| Alpha Mycolic Acids (Alpha-MA) | 3 | 1 |
| Phthiocerol Dimycocerosates (DIMA) | 2 | 1 |
| Glucose Monomycolates (GMM) | 6 | 6 |
| Trehalose Monomycolates (TMM) | 3 | 1 |
| Phthiodiolone Dimycocerosates (DIMB) | 2 | 1 |

A2

| **Sub-class of Glycerolipids** | **MTB** | ***M. kansasii*** |
| --- | --- | --- |
| Monoacylglycerols (MG) | 37 | 24 |
| Diacylglycerols (DG) | 33 | 26 |
| Triacylglycerols (TG) | 32 | 26 |

A3

| **Sub-class of Glycerophospholipids** | **MTB 1** | ***M. kansasii*** |
| --- | --- | --- |
| Monoacylglycerophosphoglycerols (Lyso-GP) | 11 | 9 |
| Monoacylglycerolphosphoethanolamines (Lyso-PE) | 6 | 8 |
| Monoacylglycerophosphoinositols (Lyso-PI) | 6 | 4 |
| Monoacylglycerophosphoinositolmonomannosides (Lyso-PIM1) | 3 | 2 |
| Monoacylglycerophosphoinositoldimannosides (Lyso-PIM2) | 2 | 2 |
| Monoacylglycerophosphoinositoltrimannosides (Lyso-PIM3) | 1 | 0 |
| Monoacylglycerophosphoinositoltetramannosides (Lyso-PIM4) | 1 | 0 |
| Monoacylglycerophosphoinositolpentamannosides (Lyso-PIM5) | 1 | 0 |
| Monoacylglycerophosphoinositolhexamannosides (Lyso-PIM6) | 1 | 0 |
|  |  |  |
| **Sub-class of Glycerophospholipids** | **MTB 1** | ***M. kansasii*** |
| Diacylglycerophosphoglycerols (PG) | 12 | 11 |
| Diacylglycerophosphoglycerophosphodiradylglycerols (CL) | 7 | 5 |
| Diacylglycerolphosphoethanolamines (PE) | 15 | 17 |
| Diacylglycerophosphoinositols (PI) | 10 | 6 |
| Diacylglycerophosphoinositolmonomannosides (PIM1) | 4 | 5 |
| Diacylglycerophosphoinositoldimannosides (PIM2) | 3 | 4 |
| Diacylglycerophosphoinositoltrimannosides (PIM3) | 2 | 2 |
| Diacylglycerophosphoinositoltetramannosides (PIM4) | 1 | 1 |
| Diacylglycerophosphoinositolpentamannosides (PIM5) | 1 | 0 |
| Monoacylated diacylglycerophosphoinositolmonomannosides (Ac1PIM1) | 6 | 2 |
| Monoacylated diacylglycerophosphoinositoldimannosides (Ac1PIM2) | 3 | 3 |
| Monoacylated diacylglycerophosphoinositoltrimannosides (Ac1PIM3) | 3 | 1 |
| Diacylated diacylglycerophosphoinositoldimannosides (Ac2PIM2) | 1 | 0 |

A4

| **Sub-class of Polyketides** | **MTB** | ***M. kansasii*** |
| --- | --- | --- |
| Mannosyl-b1-phosphomycoketides (MPM) | 2 | 0 |
| Non-ribosomal peptides/polyketide hybrids | 3 | 4 |

A5

| **Sub-class of Prenol Lipids** | **MTB** | ***M. kansasii*** |
| --- | --- | --- |
| Ubiquinones | 2 | 1 |
| Bactoprenols | 0 | 1 |

A6

| **Sub-class of Saccharolipids** | **MTB** | ***M. kansasii*** |
| --- | --- | --- |
| Diacylated Sulfolipid (Ac2SGL) | 2 | 1 |
| 2,3-di-O-acyltrehaloses (DAT1) | 1 | 1 |
| 2,3-di-O-acyltrehaloses (DAT2) | 2 | 2 |

**(B) CWL extract**

B1

| **Sub-class of Fatty Acyls** | **MTB** | ***M. kansasii*** |
| --- | --- | --- |
| Branched Fatty Acids | 39 | 33 |
| Keto Mycolic Acids (Keto-MA) | 1 | 1 |
| Methoxy Mycolic Acids (Methoxy-MA) | 2 | 1 |
| Alpha Mycolic Acids (Alpha-MA) | 1 | 2 |
| Phthiocerol Dimycocerosates (DIMA) | 2 | 1 |
| Glucose Monomycolates (GMM) | 3 | 2 |
| Trehalose Monomycolates (TMM) | 2 | 0 |
| Phthiodiolone Dimycocerosates (DIMB) | 1 | 1 |

B2

| **Sub-class of Glycerolipids** | **MTB** | ***M. kansasii*** |
| --- | --- | --- |
| Monoacylglycerols (MG) | 29 | 20 |
| Diacylglycerols (DG) | 36 | 36 |
| Triacylglycerols (TG) | 26 | 32 |

B3

| **Sub-class of Glycerophospholipids** | **MTB** | ***M. kansasii*** |
| --- | --- | --- |
| Monoacylglycerophosphoglycerols (Lyso-GP) | 9 | 8 |
| Monoacylglycerophosphoinositols (Lyso-PI) | 7 | 3 |
| Monoacylglycerolphosphoethanolamines (Lyso-PE) | 7 | 9 |
| Monoacylglycerophosphoinositolmonomannosides (Lyso-PIM1) | 5 | 3 |
| Monoacylglycerophosphoinositoldimannosides (Lyso-PIM2) | 1 | 1 |
| Monoacylglycerophosphoinositoltrimannosides (Lyso-PIM3) | 2 | 2 |
| Monoacylglycerophosphoinositoltetramannosides (Lyso-PIM4) | 0 | 1 |
| Monoacylglycerophosphoinositolpentamannosides (Lyso-PIM5) | 1 | 0 |
|  |  |  |
| **Sub-class of Glycerophospholipids** | **MTB** | ***M. kansasii*** |
| Diacylglycerophosphoglycerols (PG) | 10 | 14 |
| Diacylglycerophosphoglycerophosphodiradylglycerols (CL) | 5 | 5 |
| Diacylglycerolphosphoethanolamines (PE) | 17 | 17 |
| Diacylglycerophosphoinositols (PI) | 9 | 8 |
| Diacylglycerophosphoinositolmonomannosides (PIM1) | 5 | 4 |
| Diacylglycerophosphoinositoldimannosides (PIM2) | 2 | 4 |
| Diacylglycerophosphoinositoltrimannosides (PIM3) | 2 | 3 |
| Diacylglycerophosphoinositoltetramannosides (PIM4) | 0 | 1 |
| Diacylglycerophosphoinositolpentamannosides (PIM5) | 0 | 1 |
| Monoacylated diacylglycerophosphoinositolmonomannosides (Ac1PIM1) | 2 | 5 |
| Monoacylated diacylglycerophosphoinositoldimannosides (Ac1PIM2) | 4 | 3 |
| Monoacylated diacylglycerophosphoinositoltrimannosides (Ac1PIM3) | 2 | 1 |
| Monoacylated diacylglycerophosphoinositoltetramannosides (Ac1PIM4) | 0 | 1 |
| Diacylated diacylglycerophosphoinositoldimannosides (Ac2PIM2) | 1 | 1 |

B4

| **Sub-class of Polyketides** | **MTB** | ***M. kansasii*** |
| --- | --- | --- |
| Mannosyl-b1-phosphomycoketides (MPM) | 3 | 2 |
| Non-ribosomal peptides/polyketide hybrids | 2 | 5 |

B5

| **Sub-class of Prenol Lipids** | **MTB** | ***M. kansasii*** |
| --- | --- | --- |
| Bactoprenol diphosphates | 1 | 0 |
| Ubiquinones | 1 | 2 |
| Bactoprenols | 1 | 0 |

B6

| **Sub-class of Saccharolipids** | **MTB** | ***M. kansasii*** |
| --- | --- | --- |
| Diacylated Sulfolipid (Ac2SGL) | 1 | 1 |
| 2,3-di-O-acyltrehaloses (DAT1) | 0 | 1 |
| 2,3-di-O-acyltrehaloses (DAT2) | 0 | 3 |

Table S3: Optimised MRM transitions and mass spectrometry parameters for each GL & GPL species.

| **Q1 (m/z)** | **Q3 (m/z)** | **Time (msec)** | **ID** | **DP (Volts)** | **EP (Volts)** | **CE (Volts)** | **CXP (Volts)** |
| --- | --- | --- | --- | --- | --- | --- | --- |
| 528.3 | 184 | 40 | d7_LPC | 80 | 10 | 42 | 15 |
| 752.6 | 184 | 40 | d7_PC | 80 | 10 | 43 | 15 |
| 711.7 | 570.7 | 40 | d7_PE | 80 | 10 | 33 | 15 |
| 847.8 | 570.8 | 40 | d7_PI | 80 | 10 | 40 | 15 |
| 754.8 | 184.1 | 40 | d7_PS | 80 | 10 | 38 | 15 |
| 510.5 | 184.1 | 40 | LPC_17:0_NS | 80 | 10 | 30 | 15 |
| 762.1 | 184.1 | 40 | PC_17:0_NS | 80 | 10 | 33 | 15 |
| 636 | 495 | 40 | PE_14:0_NS | 80 | 10 | 40 | 15 |
| 812 | 535 | 40 | PI_17:0_NS | 80 | 10 | 40 | 15 |
| 680.4 | 495.4 | 40 | PS_14:0_NS | 85 | 10 | 22 | 15 |
| 829.3 | 570.6 | 40 | d7_TG | 80 | 10 | 37 | 15 |
| 605.5 | 346.4 | 40 | d7_DG | 80 | 10 | 38 | 15 |
| 530.6 | 285.3 | 40 | DG_14:0_NS | 50 | 10 | 20 | 15 |
| 782 | 523 | 40 | TG_15:0_NS | 80 | 10 | 27 | 15 |

Table S4: Details of the calibration curves of internal and natural GL and GPL standards prepared in the medium, chloroform: methanol (1:1), containing 10 mM ammonium acetate and 0.5% formic acid.

| **Standard** | **Internal Standard** | **Range (ng/mL)** | **Slope** | **Intercept** | **Coefficient of Determination (R^2^)** | **LOD (ng/mL)** | **LOQ (ng/mL)** |
| --- | --- | --- | --- | --- | --- | --- | --- |
| PS_14:0_NS | 15:0-18:1(d7) PS | 0.98-1000 | 0.0032 | 6.83E-03 | 0.999 | 45.76 | 138.66 |
| LPC_17:0_NS | 18:1(d7) Lyso PC | 0.98-1000 | 0.0004 | 1.80E-03 | 0.998 | 47.92 | 145.21 |
| PC_17:0_NS | 15:0-18:1(d7) PC | 0.98-1000 | 0.0012 | 1.66E-02 | 0.998 | 59.11 | 179.12 |
| PE_14:0_NS | 15:0-18:1(d7) PE | 0.98-1000 | 0.00001 | -4.81E-05 | 0.994 | 109.89 | 332.99 |
| PI_17:0_NS | 15:0-18:1(d7) PI | 0.98-1000 | 0.0009 | -9.67E-03 | 0.994 | 95.12 | 288.23 |
| DG_14:0_NS | 15:0-18:1(d7) DG | 0.98-1000 | 0.0262 | 1.35E+00 | 0.994 | 99.07 | 300.21 |
| TG_15:0_NS | 15:0-18:1(d7)-15:0 TG | 0.98-1000 | 0.0019 | -5.75E-03 | 0.998 | 56.03 | 169.78 |

Table S5: Optimized transition pairs including precursor ion (Q1), product ion (Q3), and respective IDs.

| **Q1 (m/z)** | **Q3 (m/z)** | **ID** |  | **Q1 (m/z)** | **Q3 (m/z)** | **ID** |  | **Q1 (m/z)** | **Q3 (m/z)** | **ID** |
| --- | --- | --- | --- | --- | --- | --- | --- | --- | --- | --- |
| 740.676 | 495.441 | TG (42:0/FA14:0) |  | 474.4 | 257.2 | DG 12:0-12:0 |  | 622.4 | 184.1 | PC 24:0 |
| 740.676 | 467.409 | TG (42:0/FA16:0) |  | 502.4 | 257.2 | DG 12:0-14:0 |  | 706.5 | 184.1 | PC 30:0 |
| 768.708 | 523.472 | TG (44:0/FA14:0) |  | 502.4 | 285.3 | DG 12:0-14:0 |  | 720.5 | 184.1 | PC 31:0 |
| 768.708 | 495.441 | TG (44:0/FA16:0) |  | 530.5 | 257.2 | DG 12:0-16:0 |  | 734.6 | 184.1 | PC 32:0 |
| 768.708 | 467.409 | TG (44:0/FA18:0) |  | 558.5 | 257.2 | DG 12:0-18:0 |  | 748.6 | 184.1 | PC 33:0 |
| 796.7 | 551.503 | TG (46:0/FA14:0) |  | 550.4 | 333.3 | DG 12:0-18:4 |  | 746.6 | 184.1 | PC 33:1 |
| 796.7 | 523.472 | TG (46:0/FA16:0) |  | 586.5 | 257.2 | DG 12:0-20:0 |  | 762.6 | 184.1 | PC 34:0 |
| 796.7 | 495.441 | TG (46:0/FA18:0) |  | 576.5 | 359.3 | DG 12:0-20:5 |  | 760.6 | 184.1 | PC 34:1 |
| 808.7 | 563.5 | TG (47:1/FA14:0) |  | 614.6 | 257.2 | DG 12:0-22:0 |  | 774.6 | 184.1 | PC 35:1 |
| 808.7 | 535.4 | TG (47:1/FA16:0) |  | 602.5 | 385.3 | DG 12:0-22:6 |  | 790.6 | 184.1 | PC 36:0 |
| 808.7 | 537.4 | TG (47:1/FA16:1) |  | 530.5 | 285.3 | DG 14:0-14:0 |  | 788.6 | 184.1 | PC 36:1 |
| 808.7 | 521.4 | TG (47:1/FA17:0) |  | 528.5 | 285.3 | DG 14:0-14:1 |  | 802.6 | 184.1 | PC 37:1 |
| 808.7 | 509.4 | TG (47:1/FA18:1) |  | 558.5 | 285.3 | DG 14:0-16:0 |  | 816.6 | 184.1 | PC 38:1 |
| 824.7 | 579.5 | TG (48:0/FA14:0) |  | 556.5 | 311.3 | DG 14:0-16:1 |  | 804.6 | 184.1 | PC 38:7 |
| 824.7 | 551.4 | TG (48:0/FA16:0) |  | 586.5 | 285.3 | DG 14:0-18:0 |  | 826.6 | 184.1 | PC 39:3 |
| 824.7 | 523.472 | TG (48:0/FA18:0) |  | 578.5 | 333.3 | DG 14:0-18:4 |  | 844.7 | 184.1 | PC 40:1 |
| 834.8 | 589.6 | TG (49:2/FA14:0) |  | 614.6 | 285.3 | DG 14:0-20:0 |  | 830.6 | 184.1 | PC 40:8 |
| 834.8 | 561.5 | TG (49:2/FA16:0) |  | 604.5 | 359.3 | DG 14:0-20:5 |  | 848.6 | 184.1 | PC 41:6 |
| 834.8 | 563.5 | TG (49:2/FA16:1) |  | 642.6 | 285.3 | DG 14:0-22:0 |  | 468.3 | 184.1 | LPC 14:0 |
| 834.8 | 547.5 | TG (49:2/FA17:0) |  | 630.5 | 385.3 | DG 14:0-22:6 |  | 482.3 | 184.1 | LPC 15:0 |
| 834.8 | 535.5 | TG (49:2/FA18:1) |  | 526.4 | 283.3 | DG 14:1-14:1 |  | 496.3 | 184.1 | LPC 16:0 |
| 834.8 | 537.5 | TG (49:2/FA18:2) |  | 556.5 | 283.3 | DG 14:1-16:0 |  | 494.3 | 184.1 | LPC 16:1 |
| 852.8 | 607.6 | TG (50:0/FA14:0) |  | 584.5 | 283.3 | DG 14:1-18:0 |  | 510.4 | 184.1 | LPC 17:0 |
| 852.8 | 579.5 | TG (50:0/FA16:0) |  | 576.5 | 333.3 | DG 14:1-18:4 |  | 524.4 | 184.1 | LPC 18:0 |
| 852.8 | 551.503 | TG (50:0/FA18:0) |  | 612.6 | 283.3 | DG 14:1-20:0 |  | 538.4 | 184.1 | LPC 19:0 |
| 866.8 | 593.5 | TG (51:0/FA16:0) |  | 602.5 | 359.3 | DG 14:1-20:5 |  | 552.4 | 184.1 | LPC 20:0 |
| 866.8 | 579.5 | TG (51:0/FA17:0) |  | 640.6 | 283.3 | DG 14:1-22:0 |  | 542.3 | 184.1 | LPC 20:5 |
| 866.8 | 565.5 | TG (51:0/FA18:0) |  | 628.5 | 385.3 | DG 14:1-22:6 |  | 580.4 | 184.1 | LPC 22:0 |
| 860.8 | 589.5 | TG (51:3/FA16:1) |  | 586.5 | 313.3 | DG 16:0-16:0 |  | 692.5 | 551.5 | PE 32:0 |
| 860.8 | 573.5 | TG (51:3/FA17:0) |  | 584.5 | 313.3 | DG 16:0-16:1 |  | 704.6 | 563.6 | PE 33:1 |
| 860.8 | 563.5 | TG (51:3/FA18:2) |  | 614.6 | 313.3 | DG 16:0-18:0 |  | 720.6 | 579.6 | PE 34:0 |
| 860.8 | 565.5 | TG (51:3/FA18:3) |  | 642.6 | 313.3 | DG 16:0-20:0 |  | 732.6 | 591.6 | PE 35:1 |
| 880.8 | 607.5 | TG (52:0/FA16:0) |  | 632.5 | 359.3 | DG 16:0-20:5 |  | 746.6 | 605.6 | PE 36:1 |
| 880.8 | 579.5 | TG (52:0/FA18:0) |  | 658.5 | 385.3 | DG 16:0-22:6 |  | 758.6 | 617.6 | PE 37:2 |
| 880.8 | 551.503 | TG (52:0/FA20:0) |  | 582.5 | 311.3 | DG 16:1-16:1 |  | 774.6 | 633.6 | PE 38:1 |
| 884.8 | 563.5 | TG (53:5/FA20:4) |  | 612.6 | 311.3 | DG 16:1-18:0 |  | 744.5 | 467.5 | PI_26:0 |
| 908.8 | 635.5 | TG (54:0/FA16:0) |  | 604.5 | 333.3 | DG 16:1-18:4 |  | 772.6 | 495.6 | PI_28:0 |
| 908.8 | 607.5 | TG (54:0/FA18:0) |  | 640.6 | 311.3 | DG 16:1-20:0 |  | 800.6 | 523.6 | PI_30:0 |
| 908.8 | 563.5 | TG (55:7/FA22:6) |  | 630.5 | 359.3 | DG 16:1-20:5 |  | 798.6 | 521.6 | PI_30:1 |
| 934.9 | 661.6 | TG (56:1/FA16:0) |  | 668.6 | 311.3 | DG 16:1-22:0 |  | 796.6 | 519.6 | PI_30:2 |
| 934.9 | 635.6 | TG (56:1/FA18:1) |  | 656.5 | 385.3 | DG 16:1-22:6 |  | 814.6 | 537.6 | PI_31:0 |
| 960.9 | 661.6 | TG (58:2/FA18:1) |  | 642.6 | 341.3 | DG 18:0-18:0 |  | 812.6 | 535.6 | PI_31:1 |
|  |  |  |  | 634.5 | 341.3 | DG 18:0-18:4 |  | 810.6 | 533.6 | PI_31:2 |
|  |  |  |  | 670.6 | 341.3 | DG 18:0-20:0 |  | 828.6 | 551.6 | PI_32:0 |
|  |  |  |  | 660.6 | 359.3 | DG 18:0-20:5 |  | 826.6 | 549.6 | PI_32:1 |
|  |  |  |  | 698.7 | 341.3 | DG 18:0-22:0 |  | 824.6 | 547.6 | PI_32:2 |
|  |  |  |  | 686.6 | 385.3 | DG 18:0-22:6 |  | 842.6 | 565.6 | PI_33:0 |
|  |  |  |  | 638.6 | 339.3 | DG 18:1-18:1 |  | 840.6 | 563.6 | PI_33:1 |
|  |  |  |  | 632.5 | 339.3 | DG 18:1-18:4 |  | 838.6 | 561.6 | PI_33:2 |
|  |  |  |  | 668.6 | 339.3 | DG 18:1-20:0 |  | 854.6 | 577.6 | PI_34:1 |
|  |  |  |  | 658.5 | 359.3 | DG 18:1-20:5 |  | 852.6 | 575.6 | PI_34:2 |
|  |  |  |  | 696.7 | 339.3 | DG 18:1-22:0 |  | 870.7 | 593.7 | PI_35:0 |
|  |  |  |  | 684.6 | 385.3 | DG 18:1-22:6 |  | 868.7 | 591.7 | PI_35:1 |
|  |  |  |  | 634.5 | 337.3 | DG 18:2-18:2 |  | 866.6 | 589.6 | PI_35:2 |
|  |  |  |  | 666.6 | 337.3 | DG 18:2-20:0 |  | 882.7 | 605.7 | PI_36:1 |
|  |  |  |  | 656.5 | 359.3 | DG 18:2-20:5 |  | 880.7 | 603.7 | PI_36:2 |
|  |  |  |  | 682.5 | 385.3 | DG 18:2-22:6 |  | 912.7 | 635.7 | PI_38:0 |
|  |  |  |  | 630.5 | 335.3 | DG 18:3-18:3 |  | 910.7 | 633.7 | PI_38:1 |
|  |  |  |  | 664.6 | 335.3 | DG 18:3-20:0 |  | 908.7 | 631.7 | PI_38:2 |
|  |  |  |  | 654.5 | 359.3 | DG 18:3-20:5 |  | 708.6 | 523.6 | PS_30:0 |
|  |  |  |  | 692.6 | 335.3 | DG 18:3-22:0 |  | 704.5 | 519.5 | PS_30:2 |
|  |  |  |  | 680.5 | 385.3 | DG 18:3-22:6 |  | 736.6 | 551.6 | PS_32:0 |
|  |  |  |  | 626.5 | 333.3 | DG 18:4-18:4 |  | 734.6 | 549.6 | PS_32:1 |
|  |  |  |  | 662.6 | 333.3 | DG 18:4-20:0 |  | 750.6 | 565.6 | PS_33:0 |
|  |  |  |  | 652.5 | 359.3 | DG 18:4-20:5 |  | 748.6 | 563.6 | PS_33:1 |
|  |  |  |  | 690.6 | 333.3 | DG 18:4-22:0 |  | 764.6 | 579.6 | PS_34:0 |
|  |  |  |  | 688.6 | 333.3 | DG 18:4-22:1 |  | 762.6 | 577.6 | PS_34:1 |
|  |  |  |  | 678.5 | 385.3 | DG 18:4-22:6 |  | 775.6 | 590.6 | PS_35:1 |
|  |  |  |  | 698.7 | 369.4 | DG 20:0-20:0 |  | 774.6 | 589.6 | PS_35:2 |
|  |  |  |  | 688.6 | 369.4 | DG 20:0-20:5 |  | 792.7 | 607.7 | PS_36:0 |
|  |  |  |  | 726.7 | 369.4 | DG 20:0-22:0 |  | 790.7 | 605.7 | PS_36:1 |
|  |  |  |  | 714.6 | 385.3 | DG 20:0-22:6 |  |  |  |  |
|  |  |  |  | 694.6 | 367.3 | DG 20:1-20:1 |  |  |  |  |
|  |  |  |  | 686.6 | 367.3 | DG 20:1-20:5 |  |  |  |  |
|  |  |  |  | 724.7 | 367.3 | DG 20:1-22:0 |  |  |  |  |
|  |  |  |  | 712.6 | 385.3 | DG 20:1-22:6 |  |  |  |  |
|  |  |  |  | 690.6 | 365.3 | DG 20:2-20:2 |  |  |  |  |
|  |  |  |  | 722.7 | 365.3 | DG 20:2-22:0 |  |  |  |  |
|  |  |  |  | 710.6 | 385.3 | DG 20:2-22:6 |  |  |  |  |
|  |  |  |  | 686.6 | 363.3 | DG 20:3-20:3 |  |  |  |  |
|  |  |  |  | 682.5 | 363.3 | DG 20:3-20:5 |  |  |  |  |
|  |  |  |  | 754.7 | 397.4 | DG 22:0-22:0 |  |  |  |  |
|  |  |  |  | 730.5 | 385.3 | DG 22:6-22:6 |  |  |  |  |

Table S6: Absolute concentrations for all quantified lipid species from GL and GPL categories.

1. TL extract

| **Analyte**  **(Lipid species)** | **MTB (ng/ml)** | ***M. kansasii* (ng/ml)** |  | **Analyte**  **(Lipid species)** | **MTB (ng/ml)** | ***M. kansasii* (ng/ml)** |  | **Analyte**  **(Lipid species)** | **MTB (ng/ml)** | ***M. kansasii* (ng/ml)** |
| --- | --- | --- | --- | --- | --- | --- | --- | --- | --- | --- |
| TG (42:0/FA14:0) | 3.55 | 1.26 |  | DG 12:0-12:0 | 0.63 | 0.45 |  | PC 24:0 | 0.02 | 0.28 |
| TG (42:0/FA16:0) | 6.35 | 1.63 |  | DG 12:0-14:0 | 0.55 | 0.32 |  | PC 30:0 | 1.26 | 0.64 |
| TG (44:0/FA14:0) | 3.85 | 0.99 |  | DG 12:0-14:0 | 0.49 | 0.27 |  | PC 31:0 | 0.22 | 0.25 |
| TG (44:0/FA16:0) | 5.96 | 1.18 |  | DG 12:0-16:0 | 0.24 | 0.14 |  | PC 32:0 | 4.01 | 1.61 |
| TG (44:0/FA18:0) | 2.91 | 0.64 |  | DG 12:0-18:0 | 0.14 | 0.10 |  | PC 33:0 | 0.43 | 0.25 |
| TG (46:0/FA14:0) | 3.32 | 0.75 |  | DG 12:0-18:4 | 0.02 | 0.01 |  | PC 33:1 | 0.88 | 0.78 |
| TG (46:0/FA16:0) | 5.76 | 1.21 |  | DG 12:0-20:0 | 0.37 | 0.24 |  | PC 34:0 | 7.07 | 0.38 |
| TG (46:0/FA18:0) | 2.27 | 0.44 |  | DG 12:0-22:0 | 0.20 | 0.12 |  | PC 34:1 | 69.88 | 5.05 |
| TG (47:1/FA14:0) | 0.63 | 0.12 |  | DG 12:0-22:6 | 0.87 | 1.66 |  | PC 35:1 | 16.25 | 1.51 |
| TG (47:1/FA16:0) | 1.03 | 0.23 |  | DG 14:0-14:0 | 0.21 | 0.10 |  | PC 36:0 | 1.24 | 0.90 |
| TG (47:1/FA16:1) | 1.15 | 0.26 |  | DG 14:0-14:1 | 0.05 | 0.02 |  | PC 36:1 | 13.63 | 9.87 |
| TG (47:1/FA17:0) | 0.29 | 0.06 |  | DG 14:0-16:0 | 0.38 | 0.23 |  | PC 37:1 | 1.08 | 0.93 |
| TG (47:1/FA18:1) | 0.69 | 0.13 |  | DG 14:0-16:1 | 0.16 | 0.34 |  | PC 38:1 | 0.81 | 0.55 |
| TG (48:0/FA14:0) | 1.76 | 0.38 |  | DG 14:0-18:0 | 0.45 | 0.20 |  | PC 38:7 | 0.06 | 0.06 |
| TG (48:0/FA16:0) | 6.23 | 1.36 |  | DG 14:0-20:0 | 0.16 | 0.08 |  | PC 39:3 | 0.09 | 0.07 |
| TG (48:0/FA18:0) | 2.73 | 0.61 |  | DG 14:0-22:0 | 0.08 | 0.04 |  | PC 40:1 | 0.06 | 0.05 |
| TG (49:2/FA14:0) | 0.10 | 0.02 |  | DG 14:0-22:6 | 1.04 | 1.21 |  | PC 40:8 | 0.13 | 0.11 |
| TG (49:2/FA16:0) | 0.35 | 0.07 |  | DG 14:1-14:1 | 0.06 | 0.06 |  | PC 41:6 | 0.03 | 0.05 |
| TG (49:2/FA16:1) | 0.86 | 0.18 |  | DG 14:1-16:0 | 0.34 | 0.23 |  | LPC 14:0 | 0.30 | 0.03 |
| TG (49:2/FA17:0) | 0.18 | 0.03 |  | DG 14:1-18:0 | 0.11 | 0.21 |  | LPC 15:0 | 0.19 | 0.20 |
| TG (49:2/FA18:1) | 0.62 | 0.11 |  | DG 14:1-20:0 | 0.96 | 0.04 |  | LPC 16:0 | 1.62 | 1.40 |
| TG (49:2/FA18:2) | 0.31 | 0.06 |  | DG 14:1-22:0 | 0.02 | 0.05 |  | LPC 16:1 | 0.18 | 0.15 |
| TG (50:0/FA14:0) | 0.58 | 0.12 |  | DG 14:1-22:6 | 0.03 | 0.02 |  | LPC 17:0 | 0.86 | 4.95 |
| TG (50:0/FA16:0) | 4.01 | 0.85 |  | DG 16:0-16:0 | 7.72 | 5.25 |  | LPC 18:0 | 1.39 | 1.36 |
| TG (50:0/FA18:0) | 3.63 | 0.76 |  | DG 16:0-16:1 | 0.73 | 0.41 |  | LPC 20:5 | 1.45 | 6.40 |
| TG (51:0/FA16:0) | 0.11 | 0.02 |  | DG 16:0-18:0 | 4.85 | 2.60 |  | LPC 22:0 | 0.04 | 0.15 |
| TG (51:0/FA17:0) | 0.08 | 0.02 |  | DG 16:0-20:0 | 0.34 | 0.22 |  | PE 32:0 | 25.13 | 13.94 |
| TG (51:0/FA18:0) | 0.23 | 0.06 |  | DG 16:0-20:5 | 0.50 | 0.12 |  | PE 33:1 | 24.57 | 16.94 |
| TG (51:3/FA16:1) | 0.14 | 0.03 |  | DG 16:0-22:6 | 1.68 | 1.01 |  | PE 34:0 | 21.31 | 5.38 |
| TG (51:3/FA17:0) | 0.02 | 0.00 |  | DG 16:1-16:1 | 0.46 | 0.14 |  | PE 35:1 | 55.14 | 13.35 |
| TG (51:3/FA18:2) | 0.17 | 0.02 |  | DG 16:1-18:0 | 1.59 | 5.60 |  | PE 36:1 | 7.05 | 2.86 |
| TG (51:3/FA18:3) | 0.00 | 0.00 |  | DG 16:1-20:0 | 0.01 | 0.02 |  | PE 37:2 | 16.73 | 1.62 |
| TG (52:0/FA16:0) | 1.15 | 0.27 |  | DG 16:1-20:5 | 0.14 | 0.03 |  | PE 38:1 | 0.44 | 0.04 |
| TG (52:0/FA18:0) | 2.42 | 0.58 |  | DG 16:1-22:0 | 0.01 | 0.03 |  | PI_26:0 | 0.68 | 0.44 |
| TG (52:0/FA20:0) | 0.09 | 0.02 |  | DG 16:1-22:6 | 0.09 | 0.03 |  | PI_28:0 | 0.26 | 0.25 |
| TG (54:0/FA16:0) | 0.08 | 0.02 |  | DG 18:0-18:0 | 3.17 | 1.99 |  | PI_30:0 | 0.06 | 0.10 |
| TG (54:0/FA18:0) | 0.61 | 0.15 |  | DG 18:0-20:0 | 0.11 | 0.04 |  | PI_30:1 | 0.03 | 0.05 |
| TG (55:7/FA22:6) | 0.01 | 0.00 |  | DG 18:0-22:6 | 0.91 | 0.46 |  | PI_30:2 | 0.14 | 0.15 |
| TG (56:1/FA16:0) | 0.06 | 0.02 |  | DG 18:1-18:1 | 5.38 | 1.53 |  | PI_31:0 | 0.08 | 0.04 |
| TG (56:1/FA18:1) | 0.08 | 0.02 |  | DG 18:1-18:4 | 0.00 | 0.00 |  | PI_31:1 | 0.95 | 1.04 |
| TG (58:2/FA18:1) | 0.14 | 0.04 |  | DG 18:1-20:0 | 0.03 | 0.01 |  | PI_31:2 | 0.00 | 0.02 |
|  |  |  |  | DG 18:1-20:5 | 0.01 | 0.01 |  | PI_32:0 | 0.05 | 0.08 |
|  |  |  |  | DG 18:1-22:0 | 0.03 | 0.02 |  | PI_32:1 | 0.05 | 0.05 |
|  |  |  |  | DG 18:1-22:6 | 1.13 | 0.50 |  | PI_32:2 | 0.05 | 0.06 |
|  |  |  |  | DG 18:2-18:2 | 0.31 | 0.09 |  | PI_33:0 | 0.06 | 0.06 |
|  |  |  |  | DG 18:2-20:0 | 0.03 | 0.00 |  | PI_33:1 | 0.04 | 0.10 |
|  |  |  |  | DG 18:2-22:6 | 0.12 | 0.04 |  | PI_34:1 | 0.51 | 0.39 |
|  |  |  |  | DG 18:3-20:5 | 0.01 | 0.01 |  | PI_34:2 | 0.28 | 0.14 |
|  |  |  |  | DG 18:3-22:6 | 0.23 | 0.14 |  | PI_35:0 | 0.83 | 1.23 |
|  |  |  |  | DG 18:4-18:4 | 0.01 | 0.01 |  | PI_35:1 | 0.25 | 0.28 |
|  |  |  |  | DG 18:4-20:0 | 0.02 | 0.01 |  | PI_35:2 | 0.10 | 0.04 |
|  |  |  |  | DG 20:0-20:0 | 0.02 | 0.03 |  | PI_36:1 | 0.11 | 0.10 |
|  |  |  |  | DG 20:0-20:5 | 0.06 | 0.01 |  | PI_36:2 | 0.14 | 0.08 |
|  |  |  |  | DG 20:0-22:6 | 0.13 | 0.03 |  | PI_38:0 | 0.02 | 0.02 |
|  |  |  |  | DG 20:1-20:1 | 0.01 | 0.01 |  | PI_38:1 | 0.00 | 0.02 |
|  |  |  |  | DG 20:1-20:5 | 0.03 | 0.01 |  | PI_38:2 | 0.02 | 0.01 |
|  |  |  |  | DG 20:1-22:0 | 0.01 | 0.01 |  | PS_30:0 | 0.00 | 0.00 |
|  |  |  |  | DG 22:6-22:6 | 0.41 | 0.17 |  | PS_32:0 | 0.01 | 0.02 |
|  |  |  |  |  |  |  |  | PS_32:1 | 0.01 | 0.00 |
|  |  |  |  |  |  |  |  | PS_34:0 | 0.00 | 0.00 |
|  |  |  |  |  |  |  |  | PS_34:1 | 0.02 | 0.01 |
|  |  |  |  |  |  |  |  | PS_35:1 | 0.00 | 0.00 |
|  |  |  |  |  |  |  |  | PS_35:2 | 0.01 | 0.00 |
|  |  |  |  |  |  |  |  | PS_36:0 | 0.00 | 0.00 |
|  |  |  |  |  |  |  |  | PS_36:1 | 0.00 | 0.01 |

1. CWL extract

| **Analyte**  **(Lipid species)** | **MTB (ng/ml)** | ***M. kansasii* (ng/ml)** |  | **Analyte**  **(Lipid species)** | **MTB (ng/ml)** | ***M. kansasii* (ng/ml)** |  | **Analyte**  **(Lipid species)** | **MTB (ng/ml)** | ***M. kansasii* (ng/ml)** |
| --- | --- | --- | --- | --- | --- | --- | --- | --- | --- | --- |
| TG (42:0/FA14:0) | 37.67 | 304.30 |  | DG 12:0-12:0 | 3.80 | 4.29 |  | PC 30:0 | 19.85 | 16.95 |
| TG (42:0/FA16:0) | 45.59 | 374.38 |  | DG 12:0-14:0 | 3.20 | 3.23 |  | PC 31:0 | 6.51 | 4.08 |
| TG (44:0/FA14:0) | 42.58 | 133.30 |  | DG 12:0-14:0 | 2.02 | 2.26 |  | PC 32:0 | 166.53 | 431.49 |
| TG (44:0/FA16:0) | 60.41 | 180.18 |  | DG 12:0-16:0 | 2.98 | 2.49 |  | PC 33:0 | 3.28 | 7.66 |
| TG (44:0/FA18:0) | 19.37 | 51.62 |  | DG 12:0-18:0 | 0.37 | 0.68 |  | PC 33:1 | 9.82 | 21.63 |
| TG (46:0/FA14:0) | 11.31 | 16.55 |  | DG 12:0-20:0 | 12.51 | 6.54 |  | PC 34:0 | 48.76 | 118.89 |
| TG (46:0/FA16:0) | 16.07 | 20.02 |  | DG 12:0-22:0 | 5.15 | 3.68 |  | PC 34:1 | 206.27 | 398.09 |
| TG (46:0/FA18:0) | 4.46 | 12.38 |  | DG 12:0-22:6 | 5.49 | 6.89 |  | PC 35:1 | 6.94 | 3.77 |
| TG (47:1/FA14:0) | 2.92 | 4.86 |  | DG 14:0-14:0 | 2.45 | 3.04 |  | PC 36:0 | 8.63 | 10.10 |
| TG (47:1/FA16:0) | 5.28 | 21.61 |  | DG 14:0-14:1 | 0.43 | 0.51 |  | PC 36:1 | 43.43 | 128.60 |
| TG (47:1/FA16:1) | 5.25 | 9.52 |  | DG 14:0-16:0 | 4.02 | 4.79 |  | PC 37:1 | 5.65 | 3.01 |
| TG (47:1/FA17:0) | 1.95 | 6.18 |  | DG 14:0-16:1 | 6.66 | 3.08 |  | LPC 14:0 | 8.47 | 1.36 |
| TG (47:1/FA18:1) | 3.27 | 12.04 |  | DG 14:0-18:0 | 5.54 | 4.43 |  | LPC 15:0 | 5.34 | 3.93 |
| TG (48:0/FA14:0) | 3.08 | 9.13 |  | DG 14:0-20:0 | 5.88 | 3.58 |  | LPC 16:0 | 47.20 | 177.25 |
| TG (48:0/FA16:0) | 21.94 | 82.10 |  | DG 14:0-22:0 | 3.87 | 2.32 |  | LPC 16:1 | 3.91 | 9.49 |
| TG (48:0/FA18:0) | 4.74 | 13.01 |  | DG 14:0-22:6 | 4.17 | 7.25 |  | LPC 17:0 | 5.30 | 9.30 |
| TG (49:2/FA14:0) | 0.25 | 1.40 |  | DG 14:1-16:0 | 19.09 | 23.40 |  | LPC 18:0 | 20.20 | 51.76 |
| TG (49:2/FA16:0) | 1.24 | 2.50 |  | DG 14:1-18:0 | 22.21 | 22.05 |  | LPC 19:0 | 6.32 | 11.49 |
| TG (49:2/FA16:1) | 2.72 | 11.33 |  | DG 14:1-20:0 | 11.51 | 3.60 |  | LPC 20:0 | 4.91 | 5.52 |
| TG (49:2/FA17:0) | 0.73 | 2.31 |  | DG 16:0-16:0 | 310.36 | 162.73 |  | LPC 20:5 | 56.74 | 72.80 |
| TG (49:2/FA18:1) | 1.73 | 3.83 |  | DG 16:0-16:1 | 11.35 | 14.14 |  | PE 32:0 | 0.73 | 0.18 |
| TG (49:2/FA18:2) | 0.80 | 3.80 |  | DG 16:0-18:0 | 324.84 | 217.13 |  | PE 33:1 | 0.90 | 0.18 |
| TG (50:0/FA14:0) | 1.42 | 0.67 |  | DG 16:0-20:0 | 3.17 | 2.46 |  | PE 34:0 | 0.61 | 0.07 |
| TG (50:0/FA16:0) | 11.89 | 19.53 |  | DG 16:0-22:6 | 8.44 | 15.49 |  | PE 35:1 | 1.75 | 0.10 |
| TG (50:0/FA18:0) | 9.22 | 10.17 |  | DG 16:1-16:1 | 8.37 | 5.20 |  | PE 36:1 | 0.14 | 0.09 |
| TG (52:0/FA16:0) | 0.32 | 2.19 |  | DG 16:1-18:0 | 28.56 | 15.95 |  | PE 37:2 | 0.30 | 0.03 |
| TG (52:0/FA18:0) | 0.88 | 5.29 |  | DG 16:1-18:4 | 0.24 | 0.08 |  | PE 38:1 | 0.01 | 0.01 |
|  |  |  |  | DG 16:1-20:0 | 1.31 | 1.19 |  | PI_26:0 | 1.25 | 1.24 |
|  |  |  |  | DG 18:0-18:0 | 190.26 | 127.22 |  | PI_28:0 | 0.33 | 0.22 |
|  |  |  |  | DG 18:0-20:0 | 1.04 | 1.70 |  |  |  |  |
|  |  |  |  | DG 18:0-22:0 | 0.22 | 0.23 |  |  |  |  |
|  |  |  |  | DG 18:0-22:6 | 2.36 | 4.01 |  |  |  |  |
|  |  |  |  | DG 18:1-18:1 | 59.58 | 57.83 |  |  |  |  |
|  |  |  |  | DG 18:1-20:0 | 0.76 | 0.62 |  |  |  |  |
|  |  |  |  | DG 18:1-22:0 | 0.25 | 0.18 |  |  |  |  |
|  |  |  |  | DG 18:1-22:6 | 4.18 | 6.41 |  |  |  |  |
|  |  |  |  | DG 18:2-18:2 | 7.14 | 6.79 |  |  |  |  |
|  |  |  |  | DG 18:2-22:6 | 0.35 | 0.31 |  |  |  |  |
|  |  |  |  | DG 18:3-22:0 | 0.22 | 0.41 |  |  |  |  |
|  |  |  |  | DG 18:3-22:6 | 1.04 | 0.98 |  |  |  |  |
|  |  |  |  | DG 20:0-20:0 | 0.22 | 0.33 |  |  |  |  |
|  |  |  |  | DG 20:0-20:5 | 0.74 | 0.25 |  |  |  |  |
|  |  |  |  | DG 20:0-22:0 | 2.39 | 0.29 |  |  |  |  |
|  |  |  |  | DG 20:1-20:5 | 0.55 | 0.41 |  |  |  |  |

Fig. S1: IDA Chromatograms of MTB and *M. kansasii* isolates in Pos & Neg ionization mode. (A) and (B) Pos and Neg chromatograms of MTB isolates, (C) and (D) Pos and Neg chromatograms of *M. kansasii* isolates.

(A) MTB_Pos (B) MTB_Neg


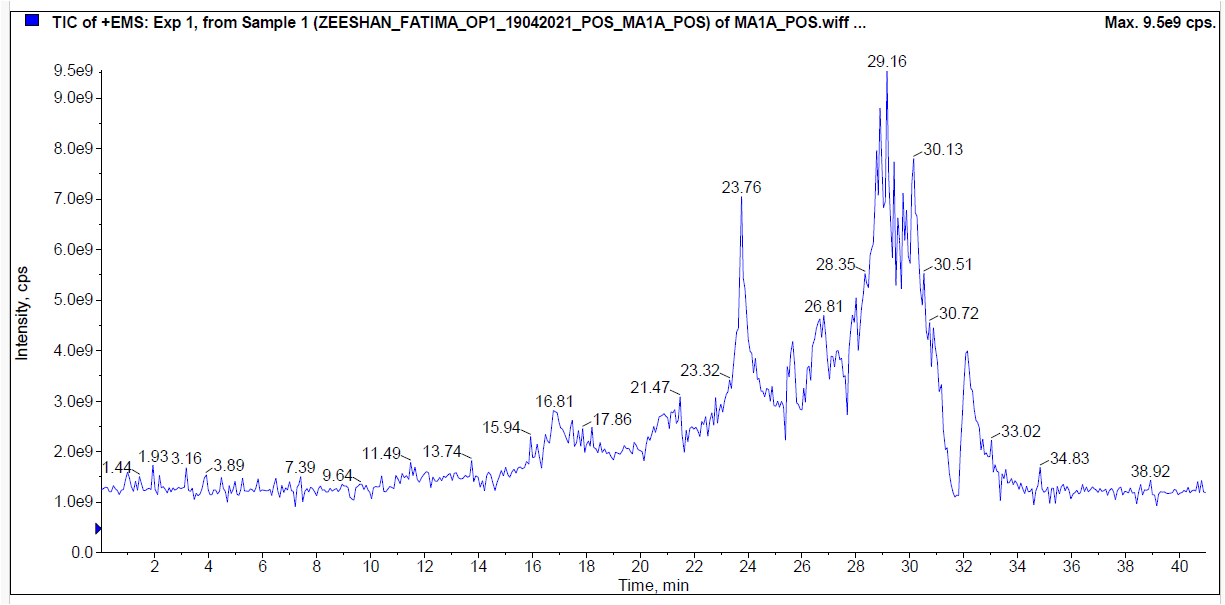

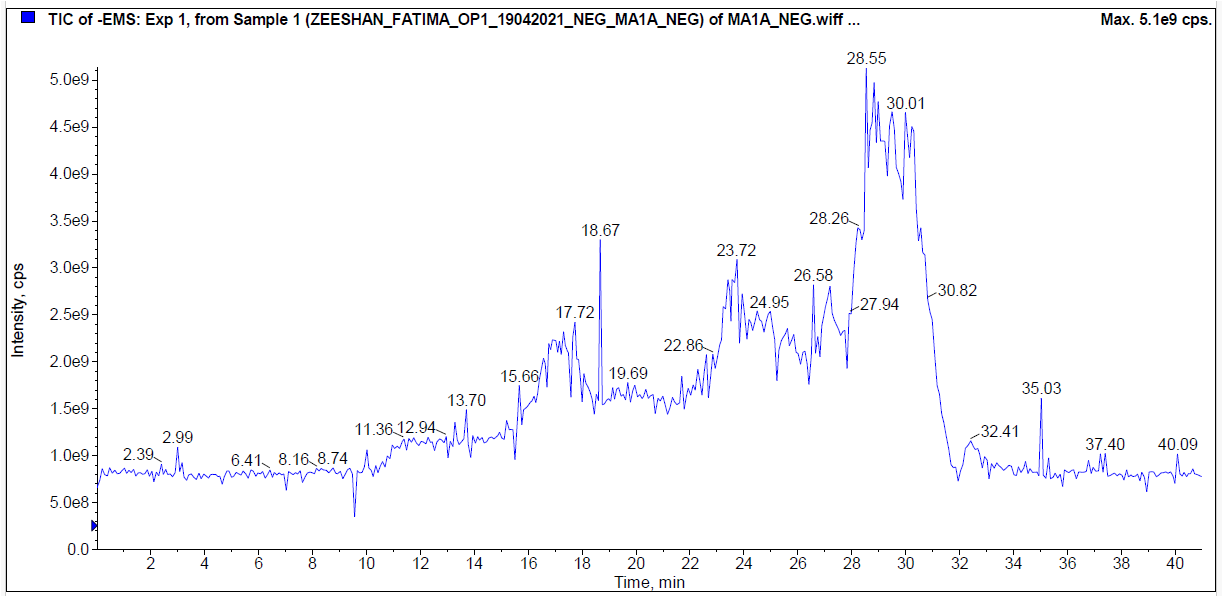


(C) *M. kansasii* _Pos (D) *M. kansasii* _Neg


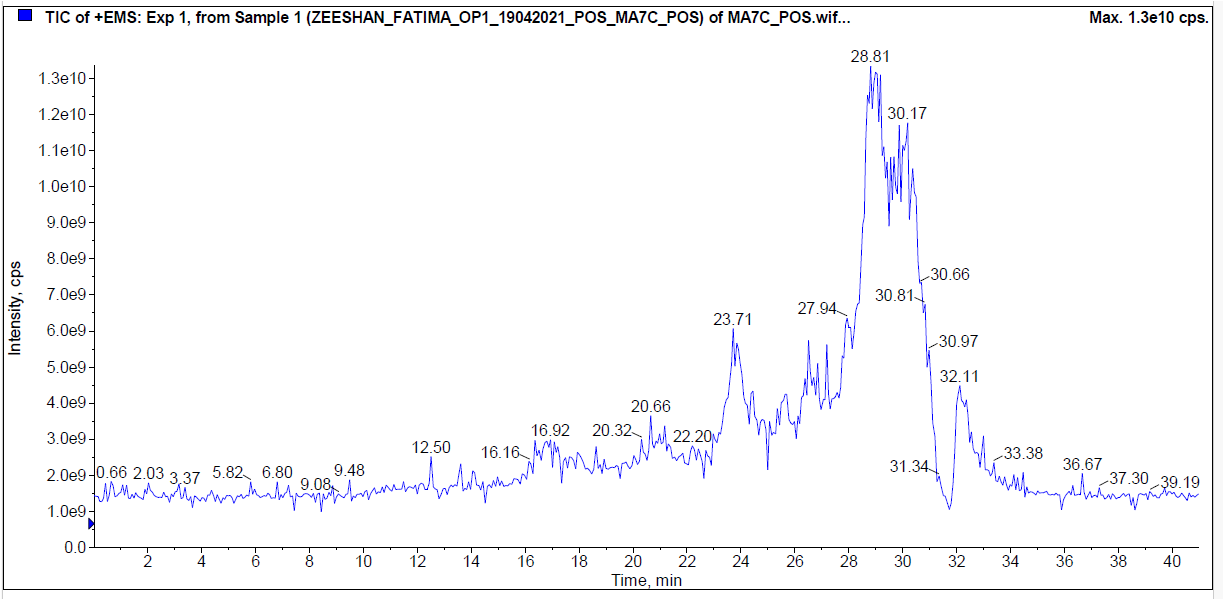

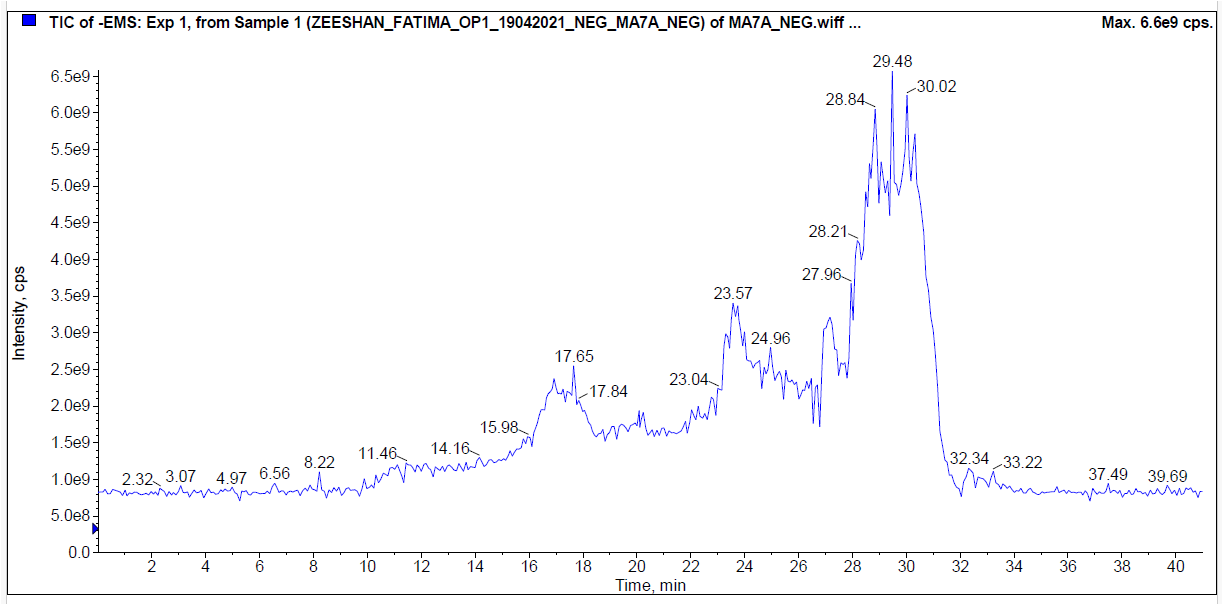


Fig S2: Chromatograms showing Q1 scan, MS2 scan, and XICs of TG, DG, PC, LPC, PE, PI, and PS natural standards from GL and GPL categories for quantitative analysis.

**(A)** **Q1 Scan**

a. TG b. DG


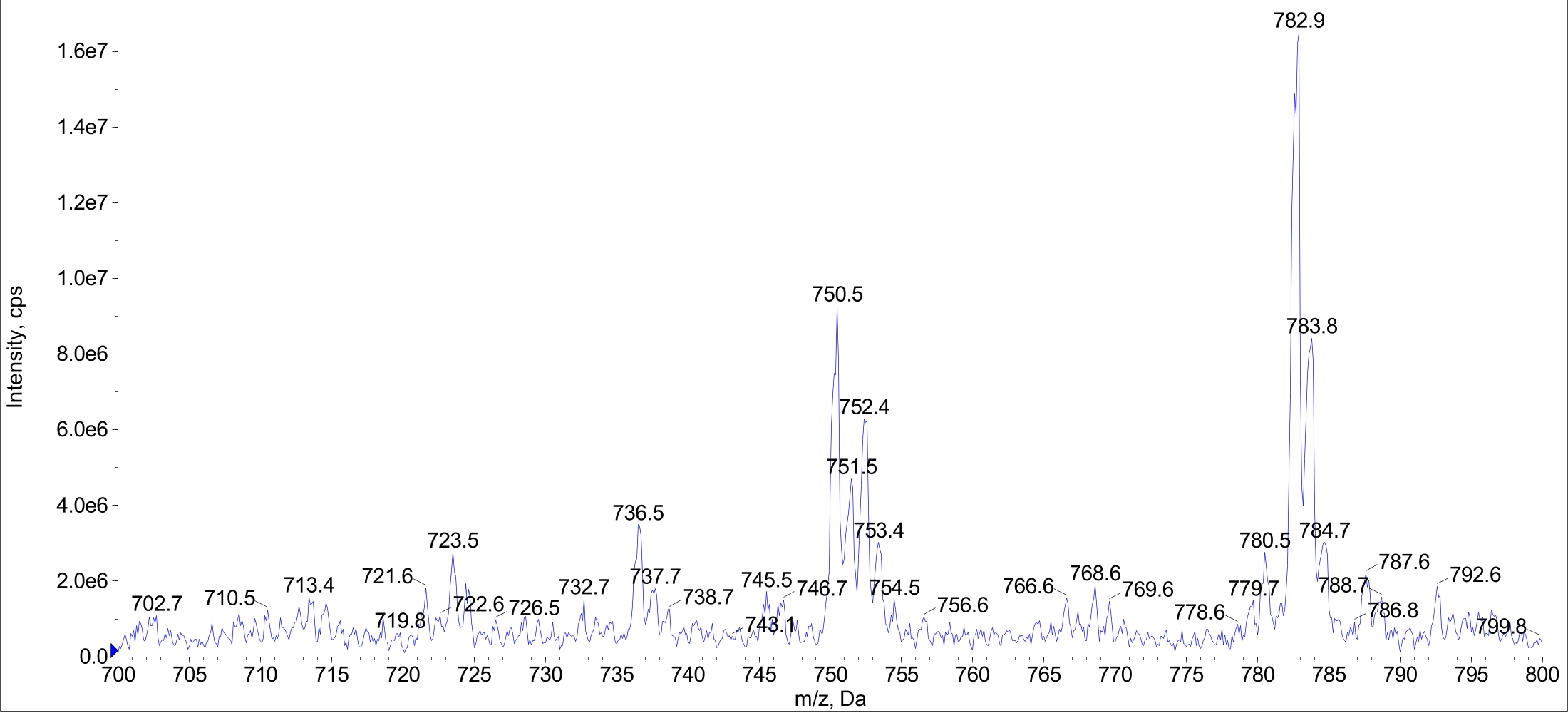

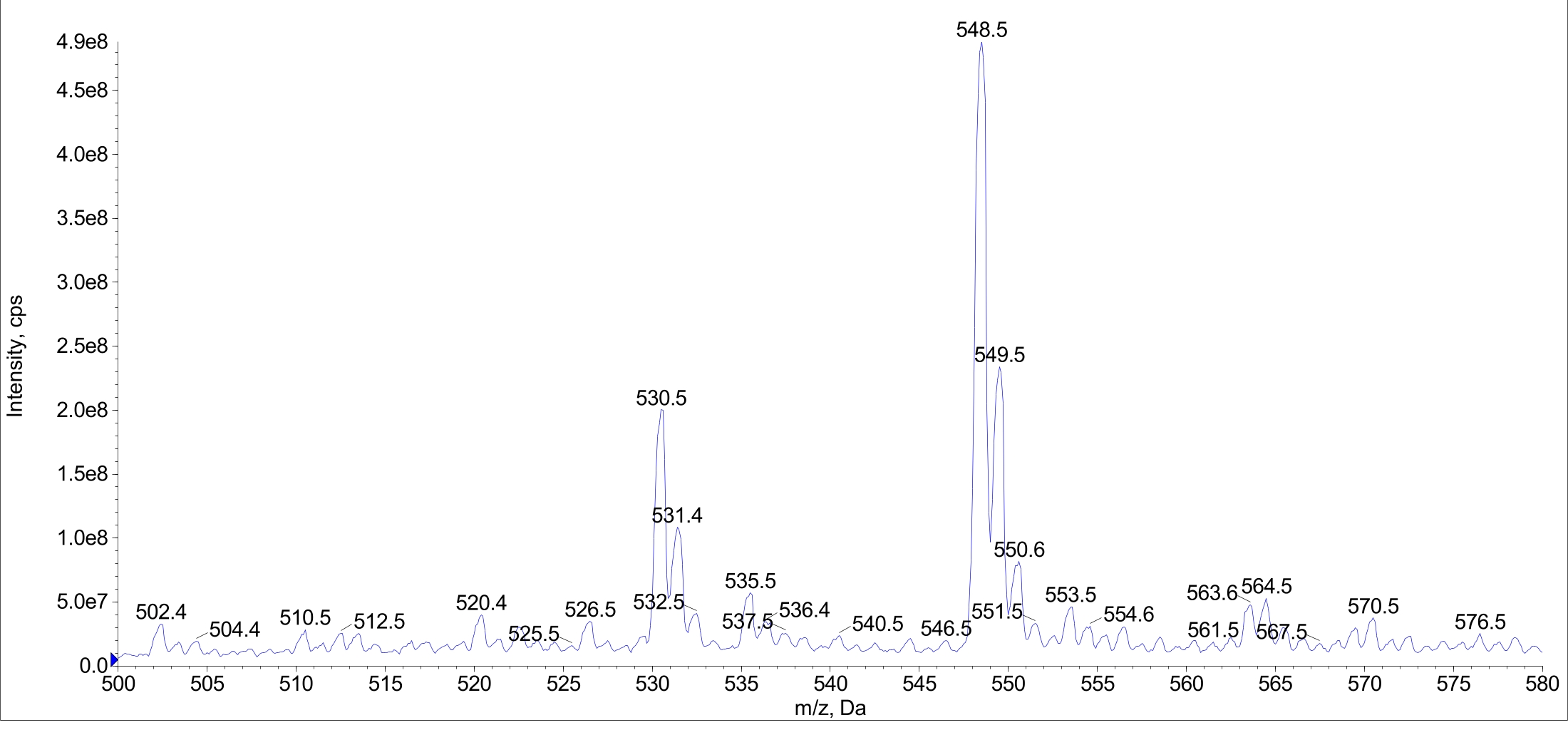


c. PC d. LPC


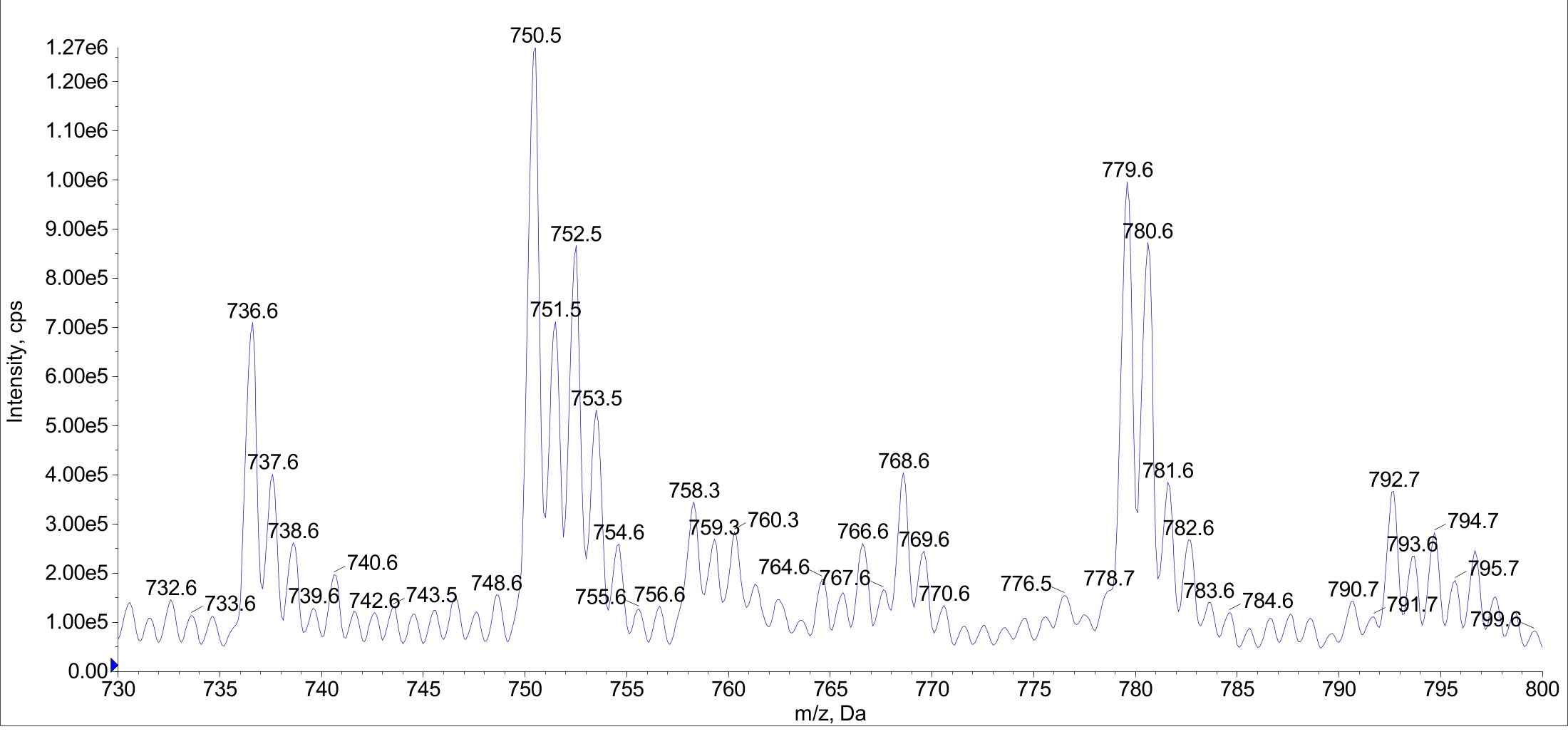

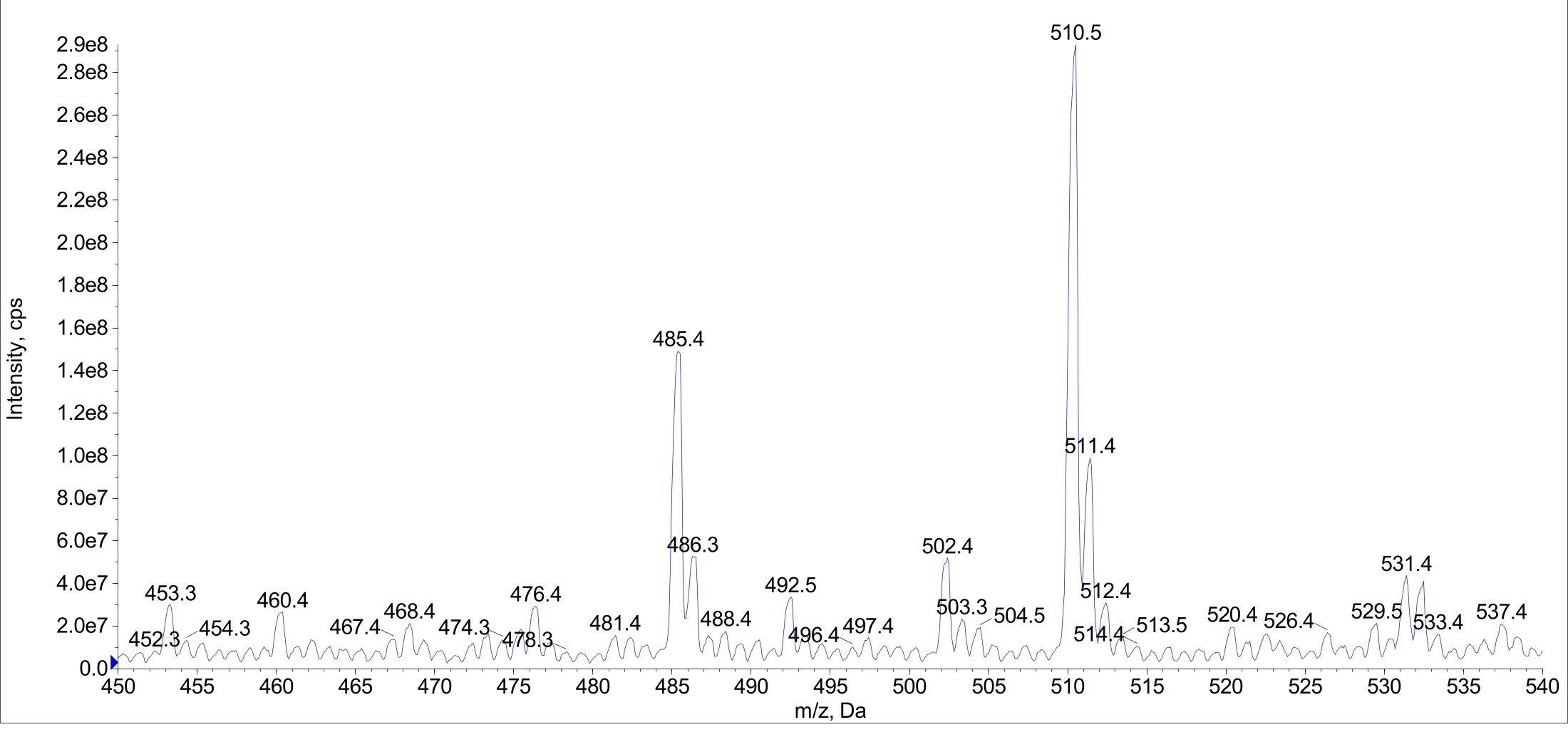


e. PE f. PI


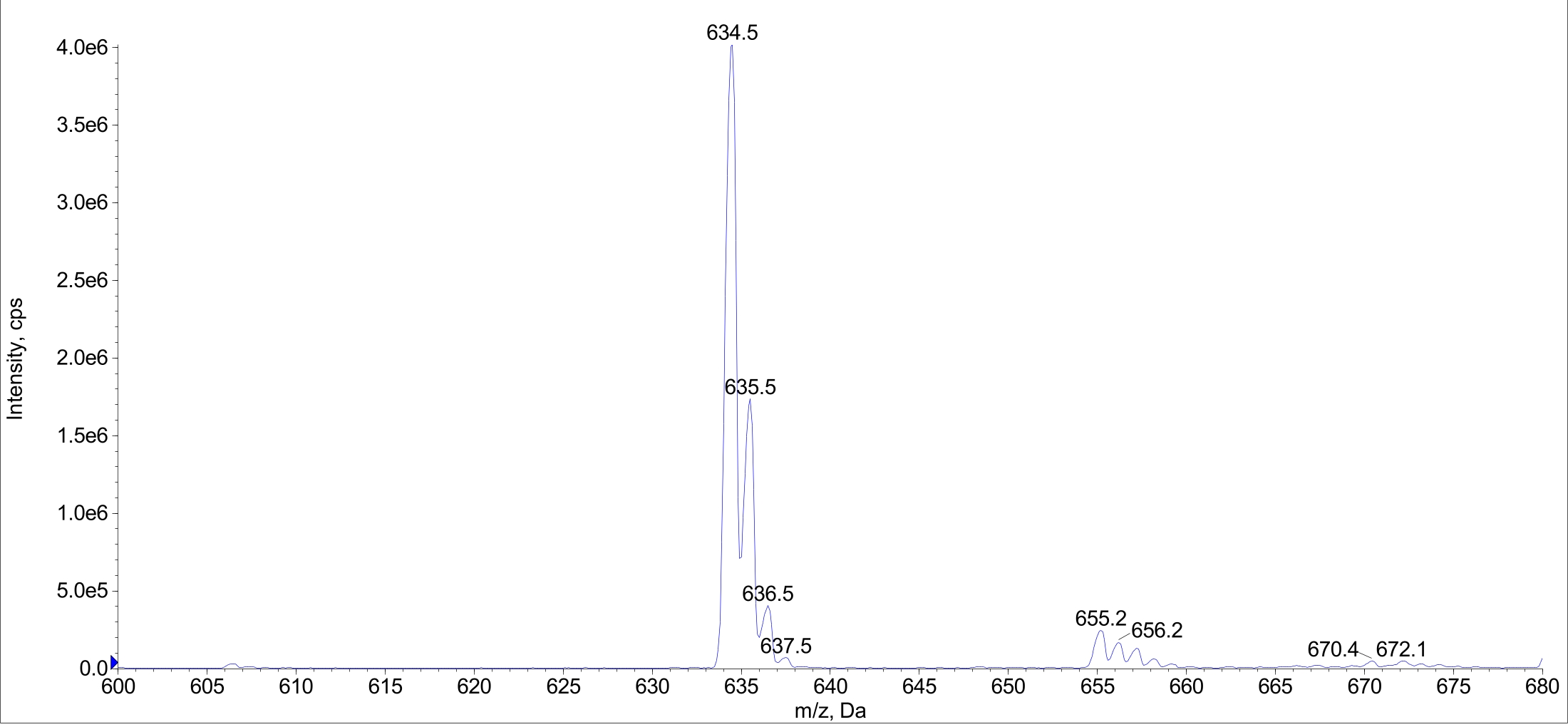

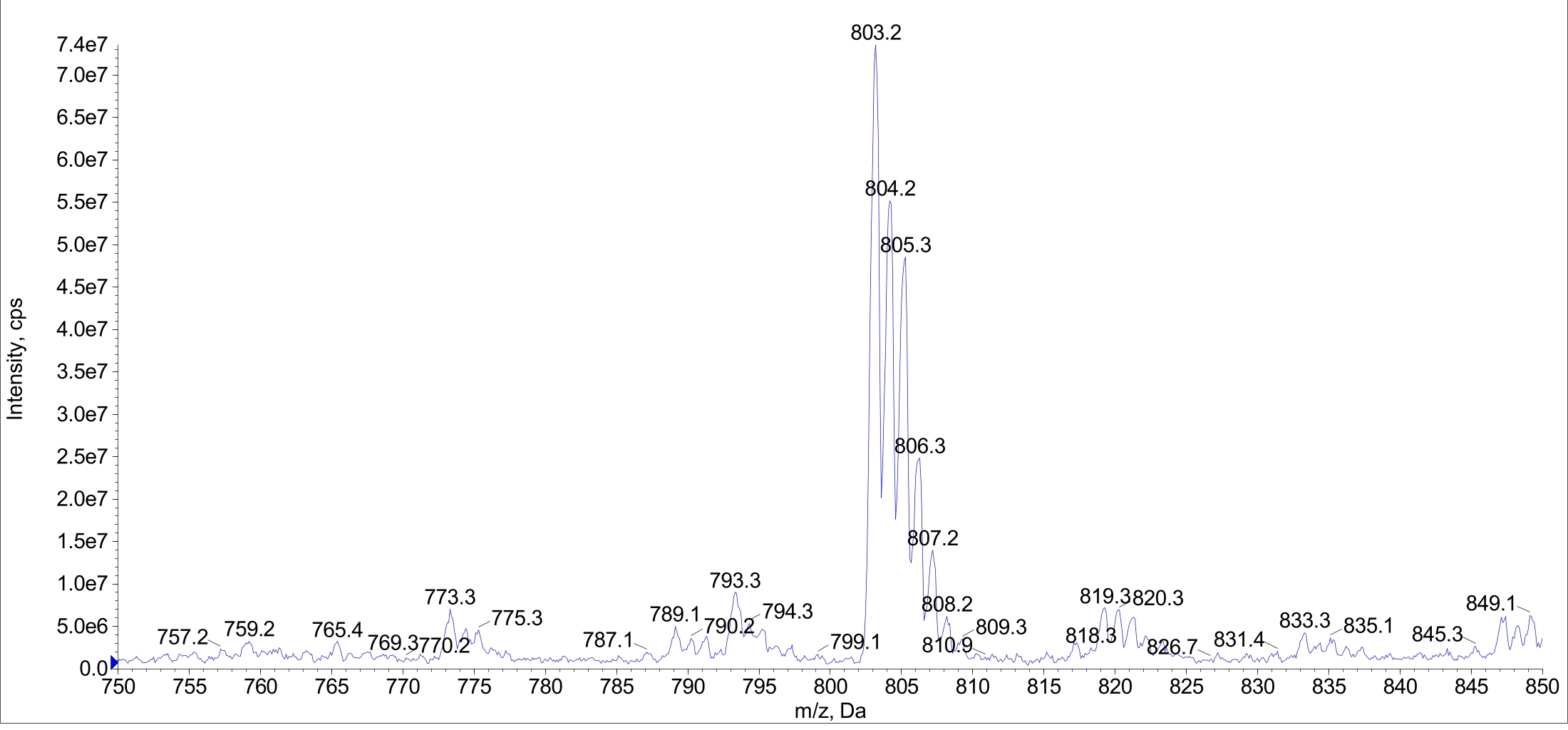


g. PS


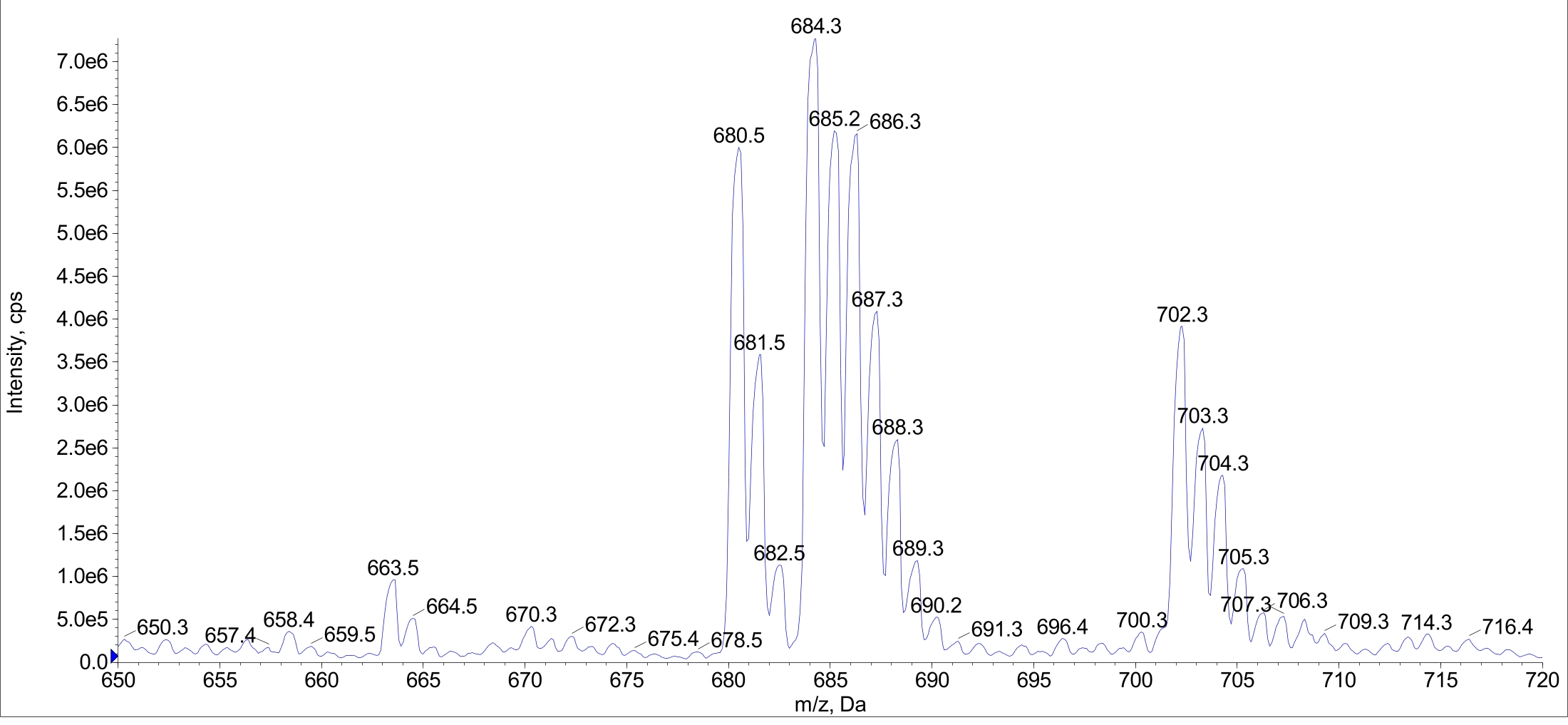


**(B) MS2 Scan**

a. TG b. DG


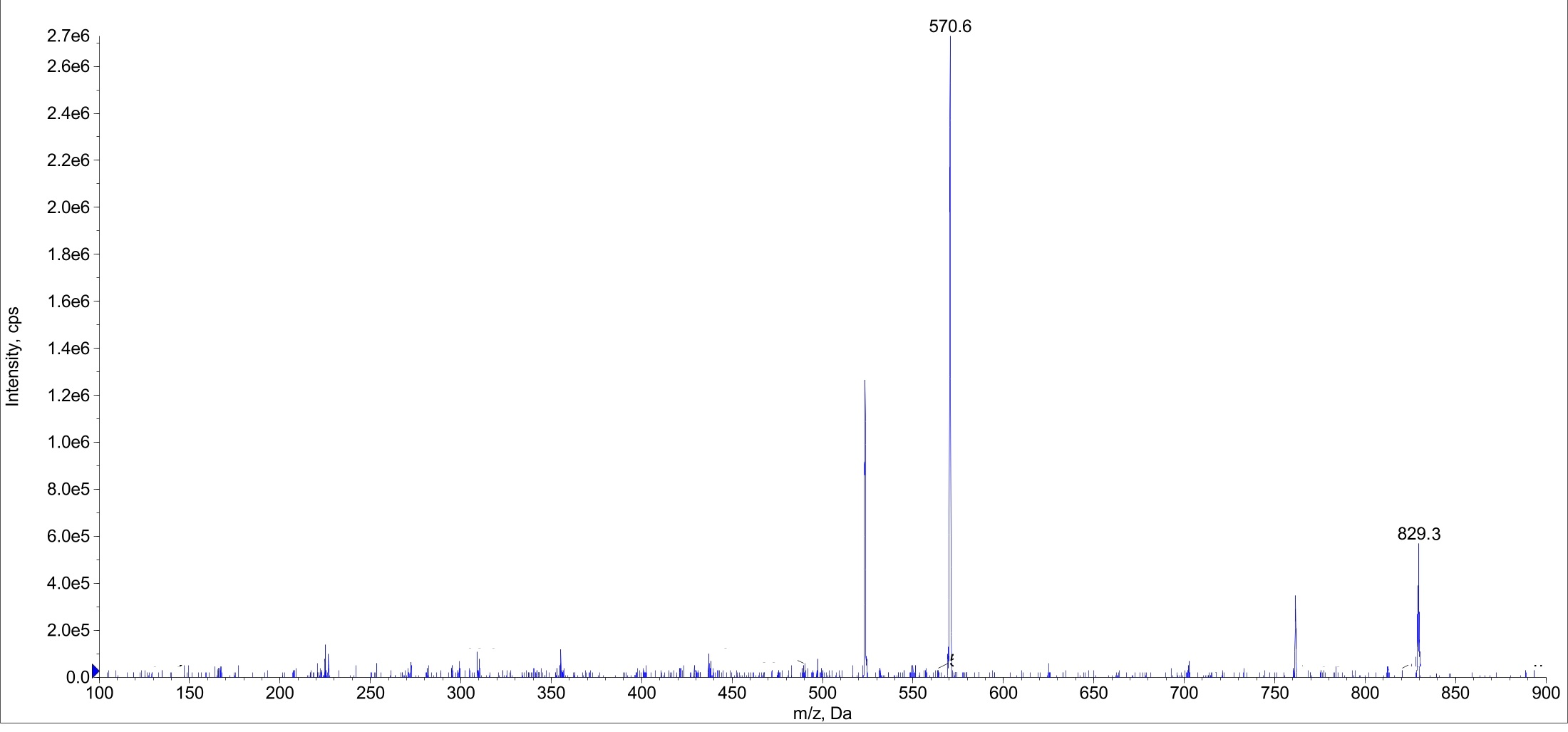

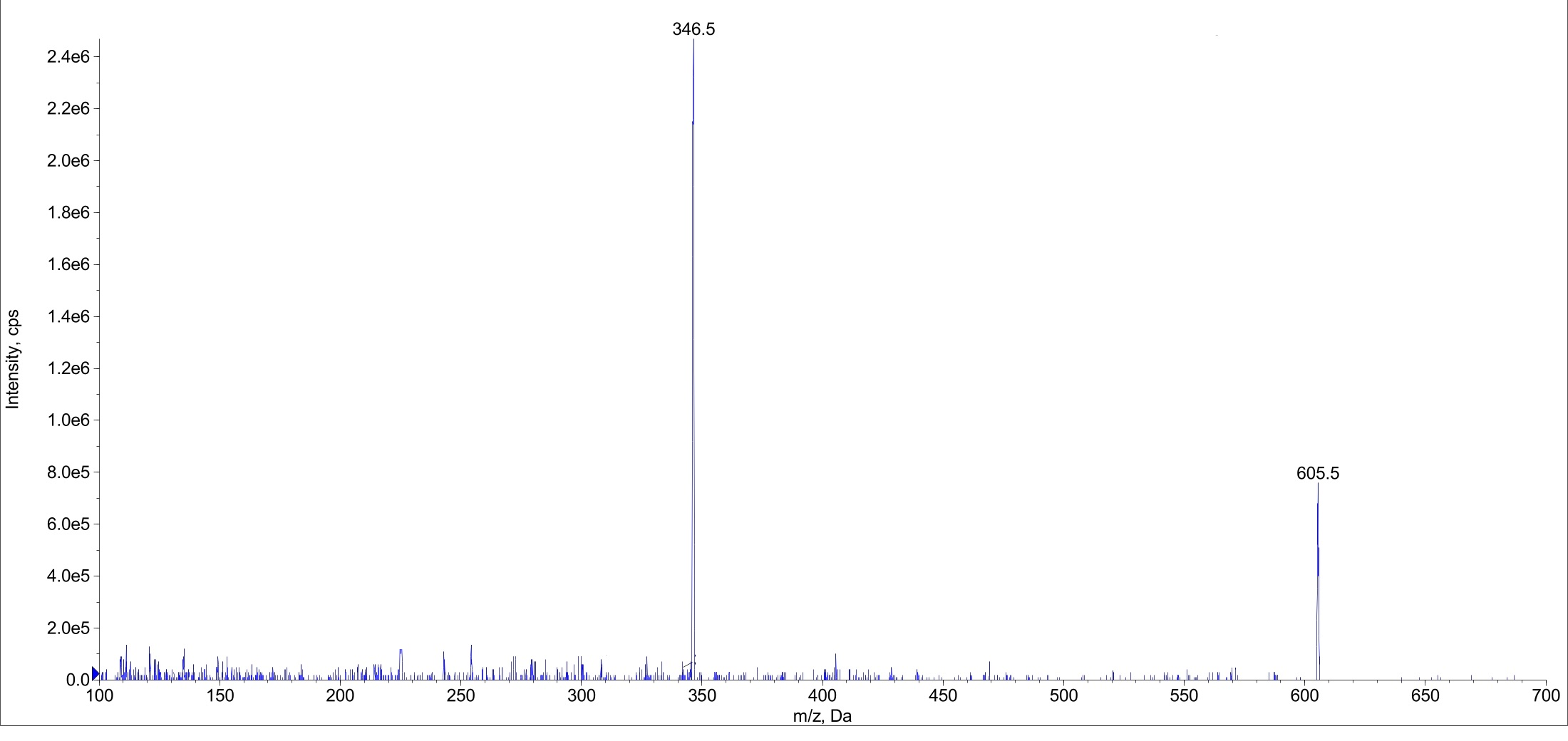


c. PC d. LPC


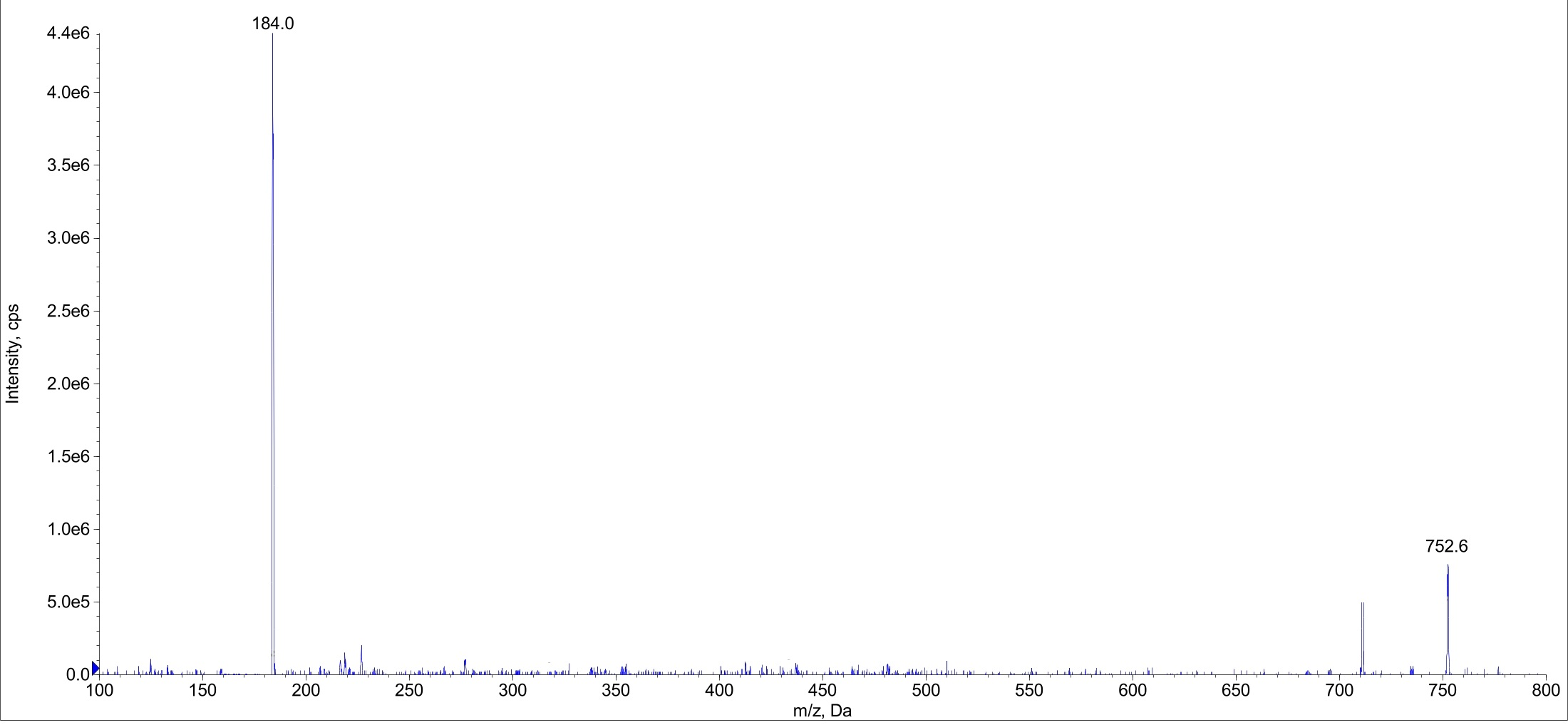

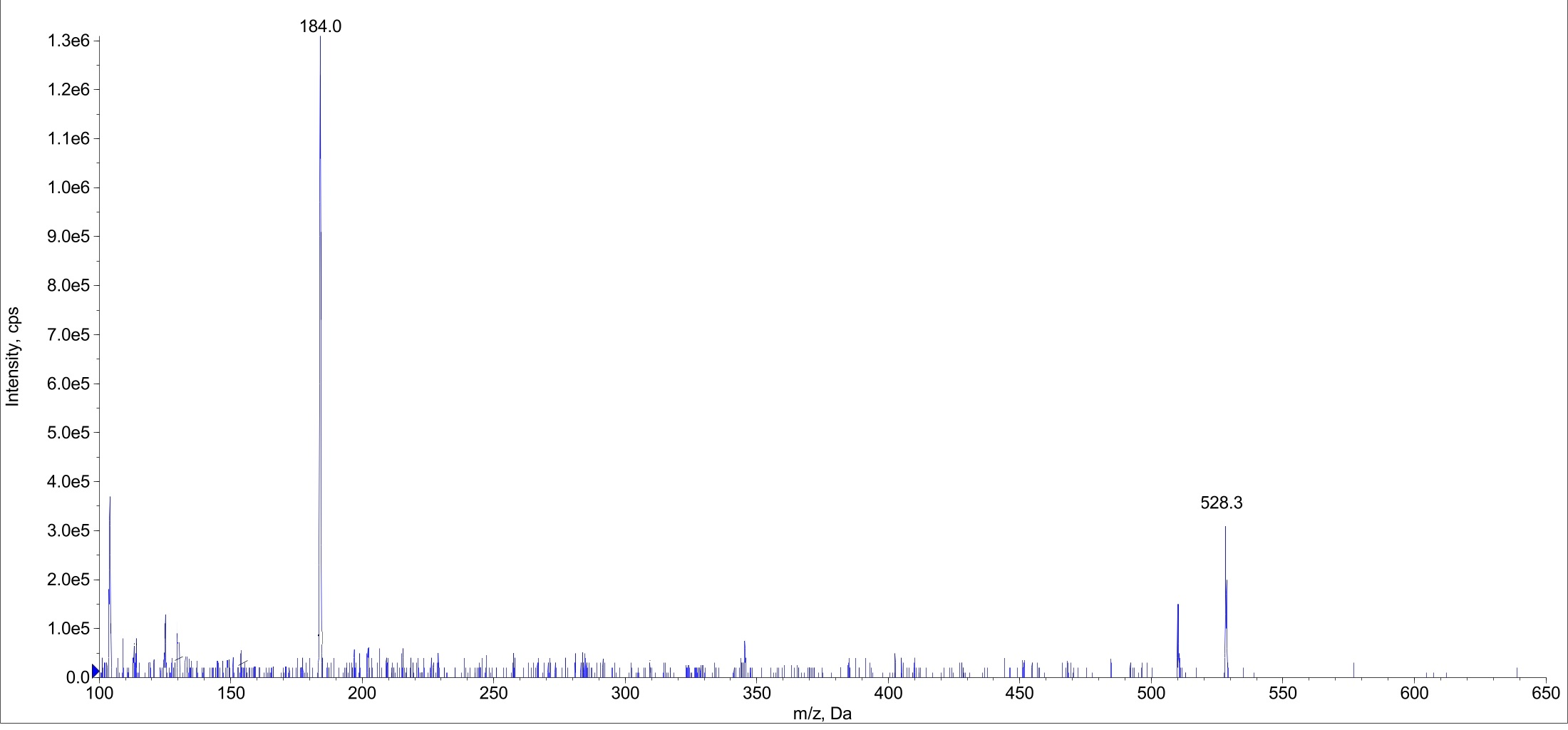


e. PE f. PI


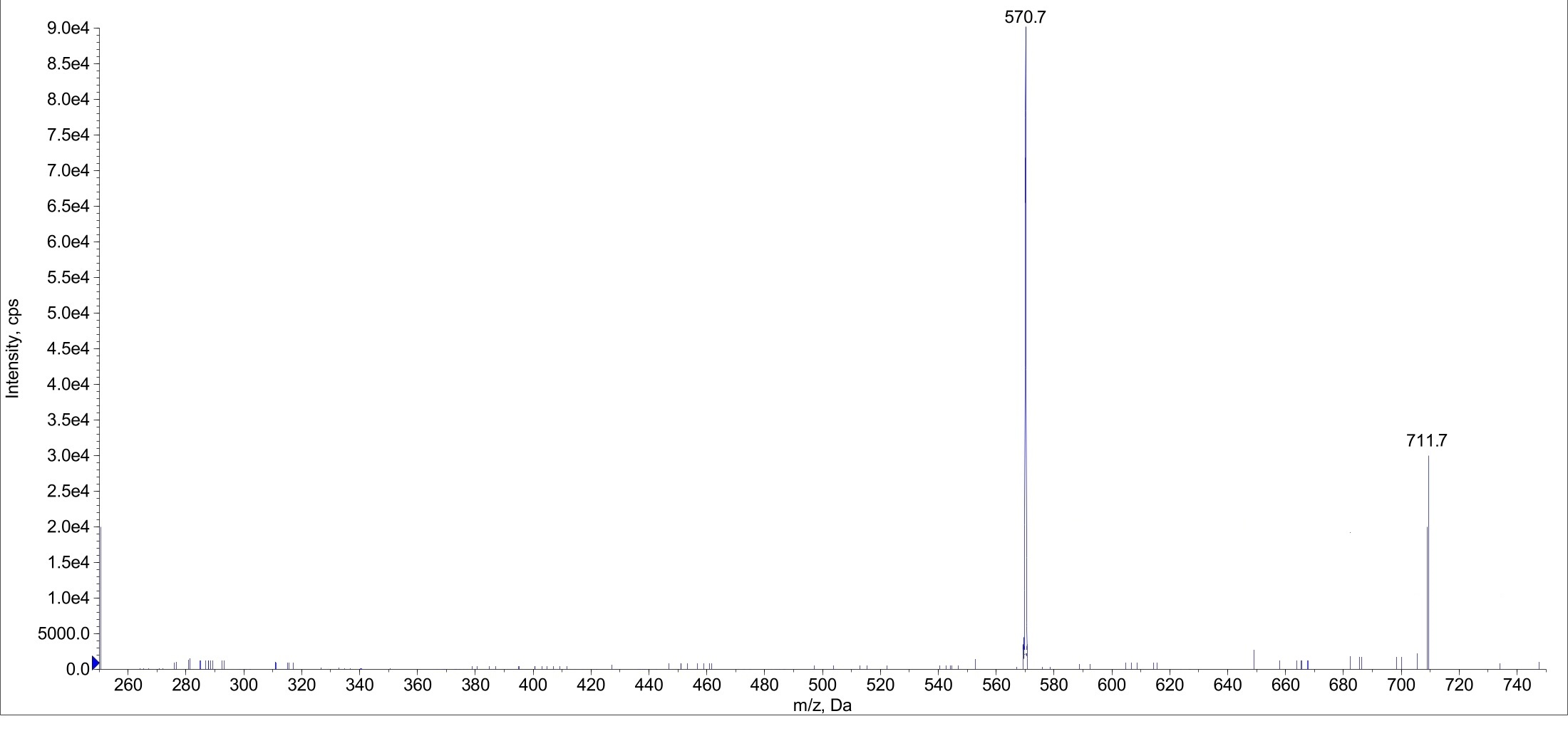

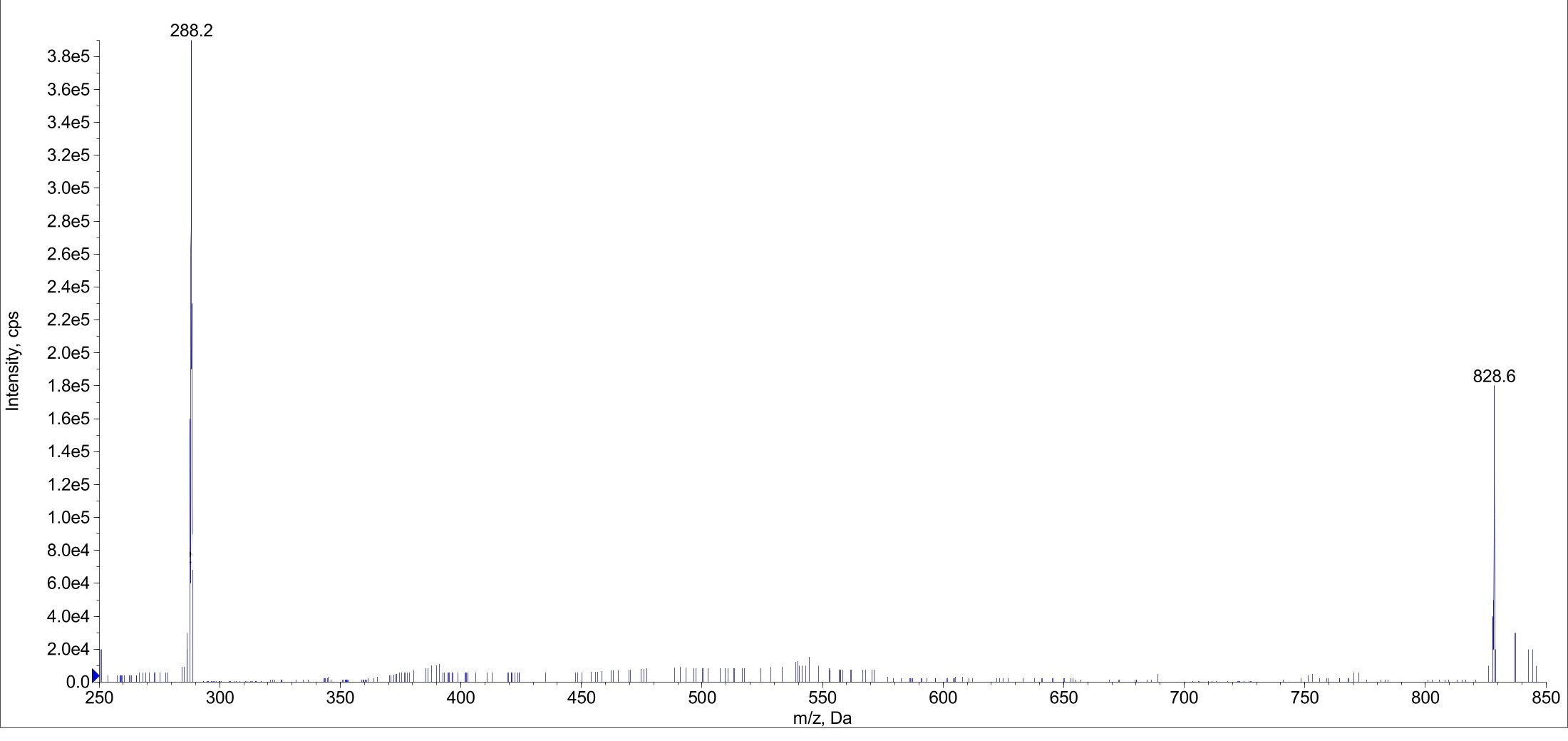


g. PS
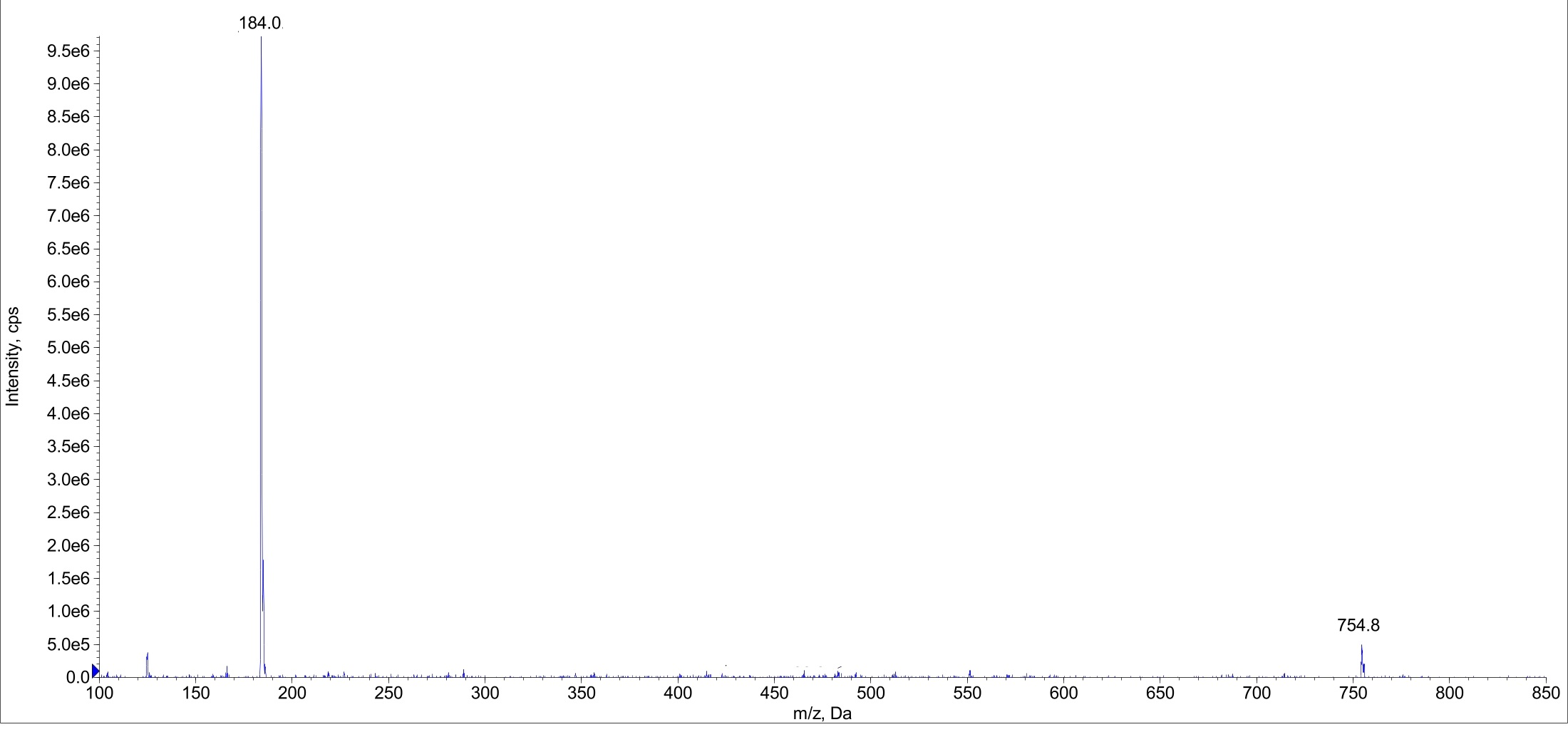


**(C) XIC chromatograms**

a. GL


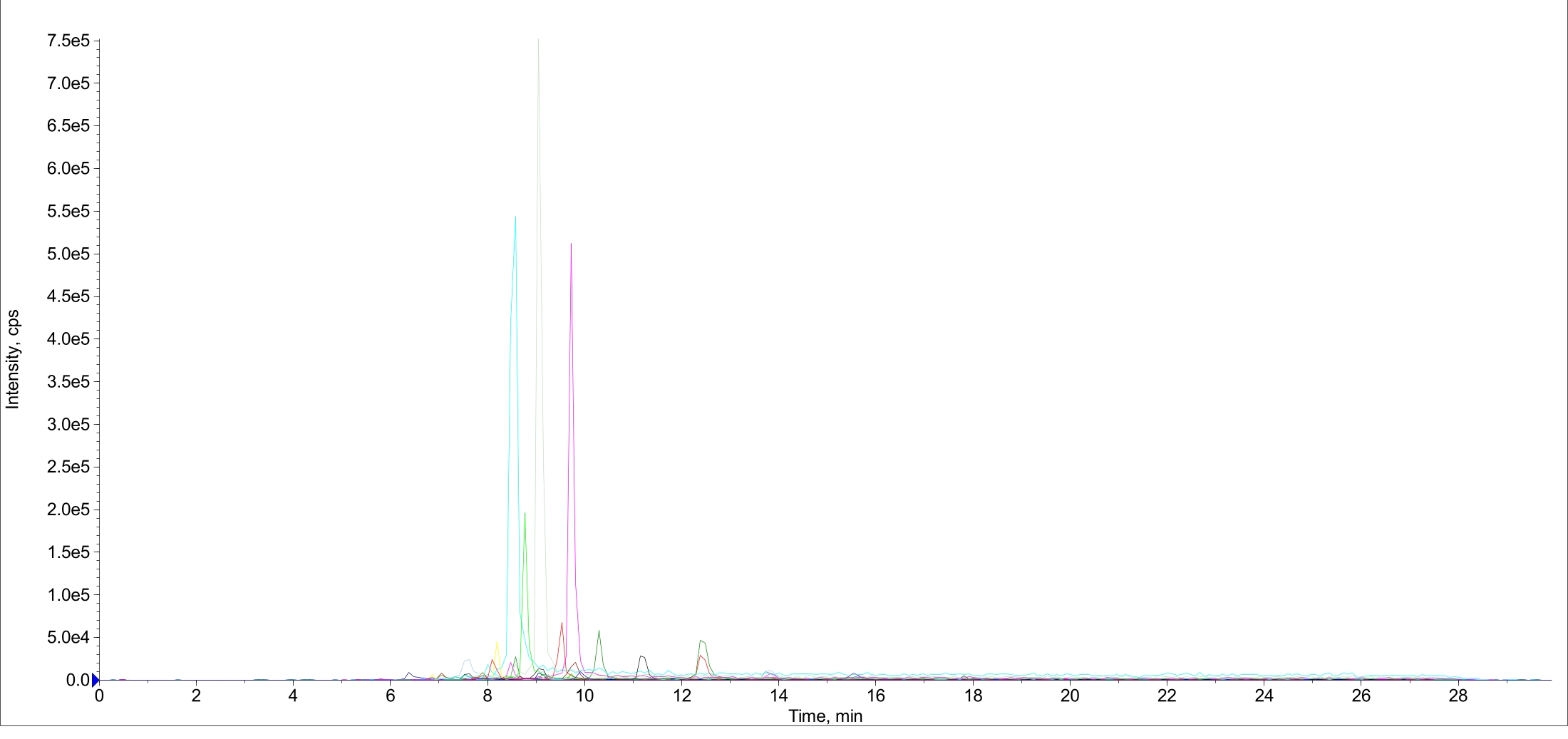


a1. TG a2. DG


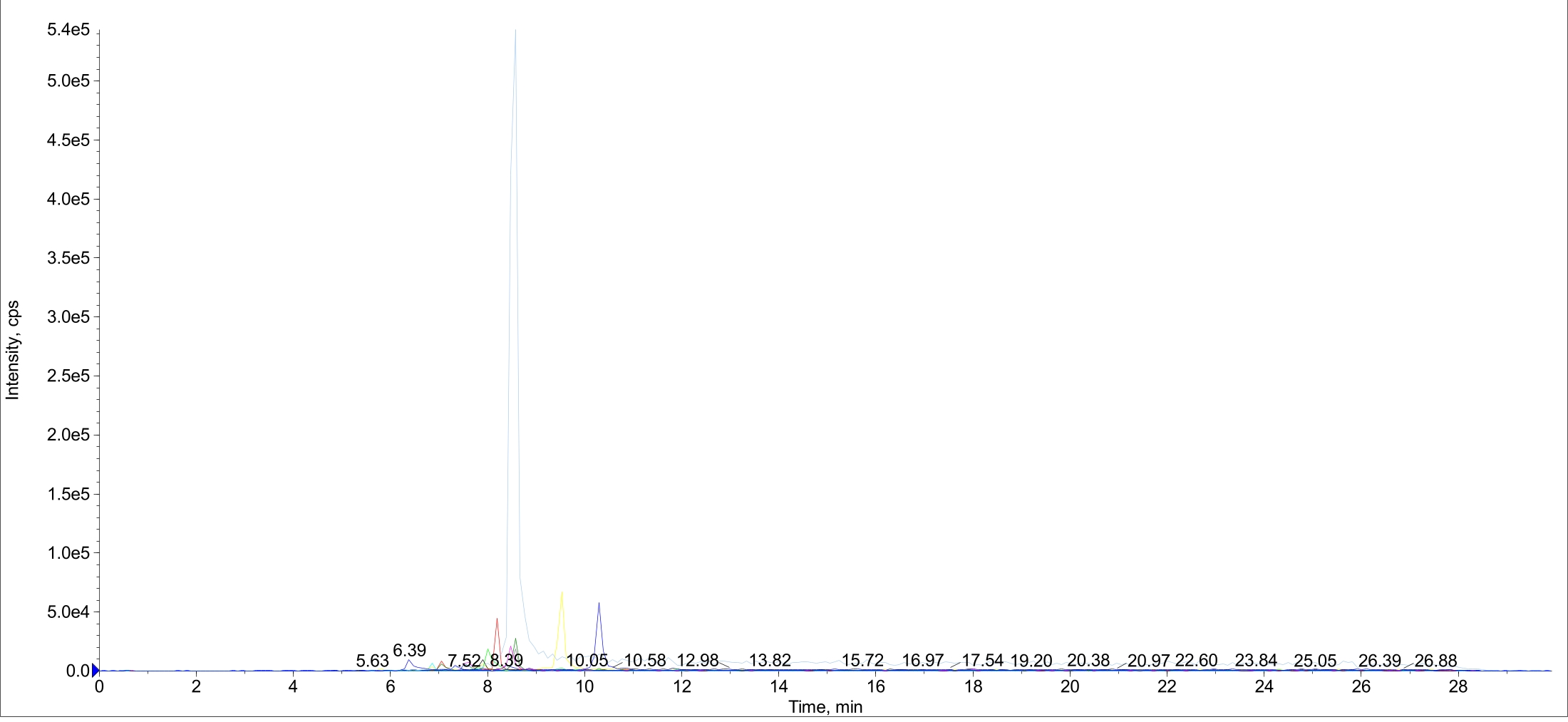

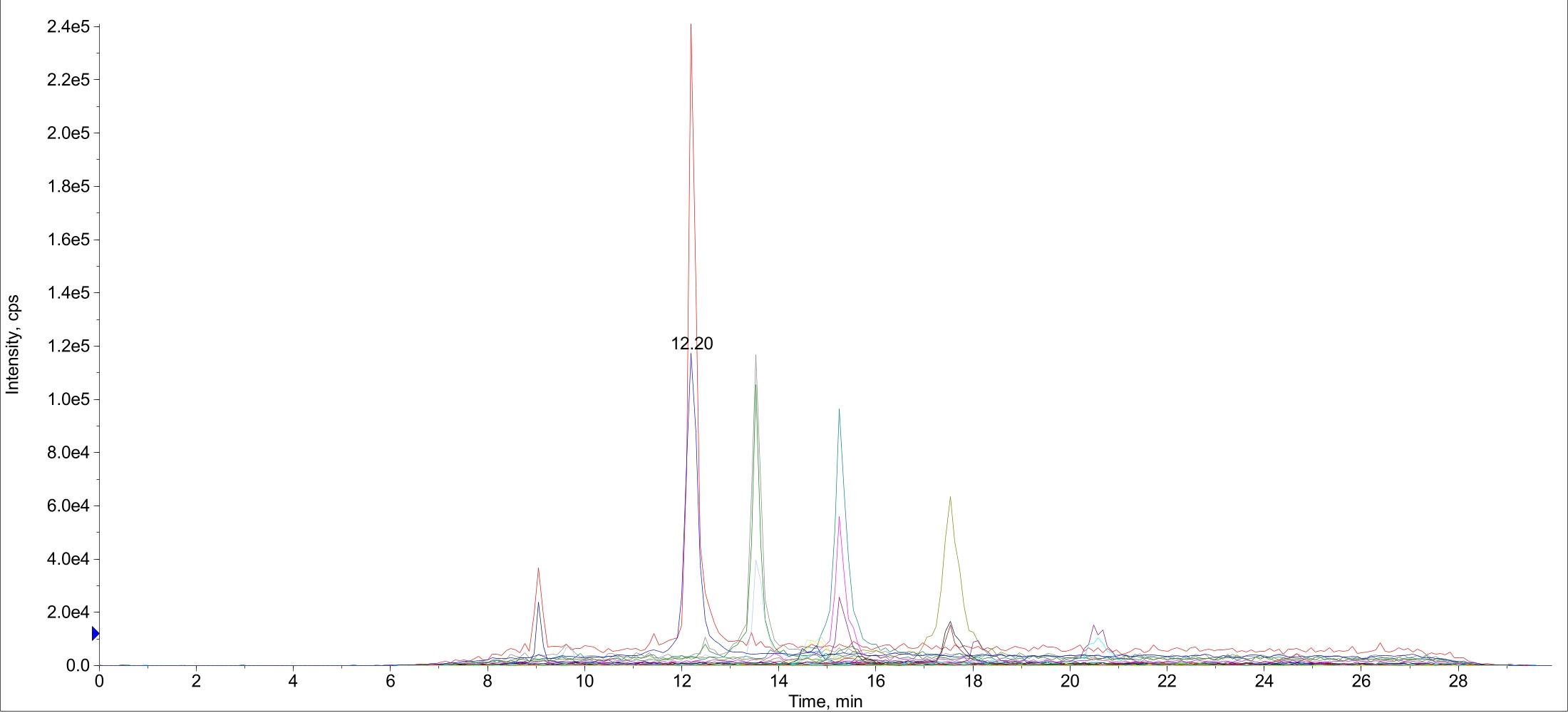


b. GLP


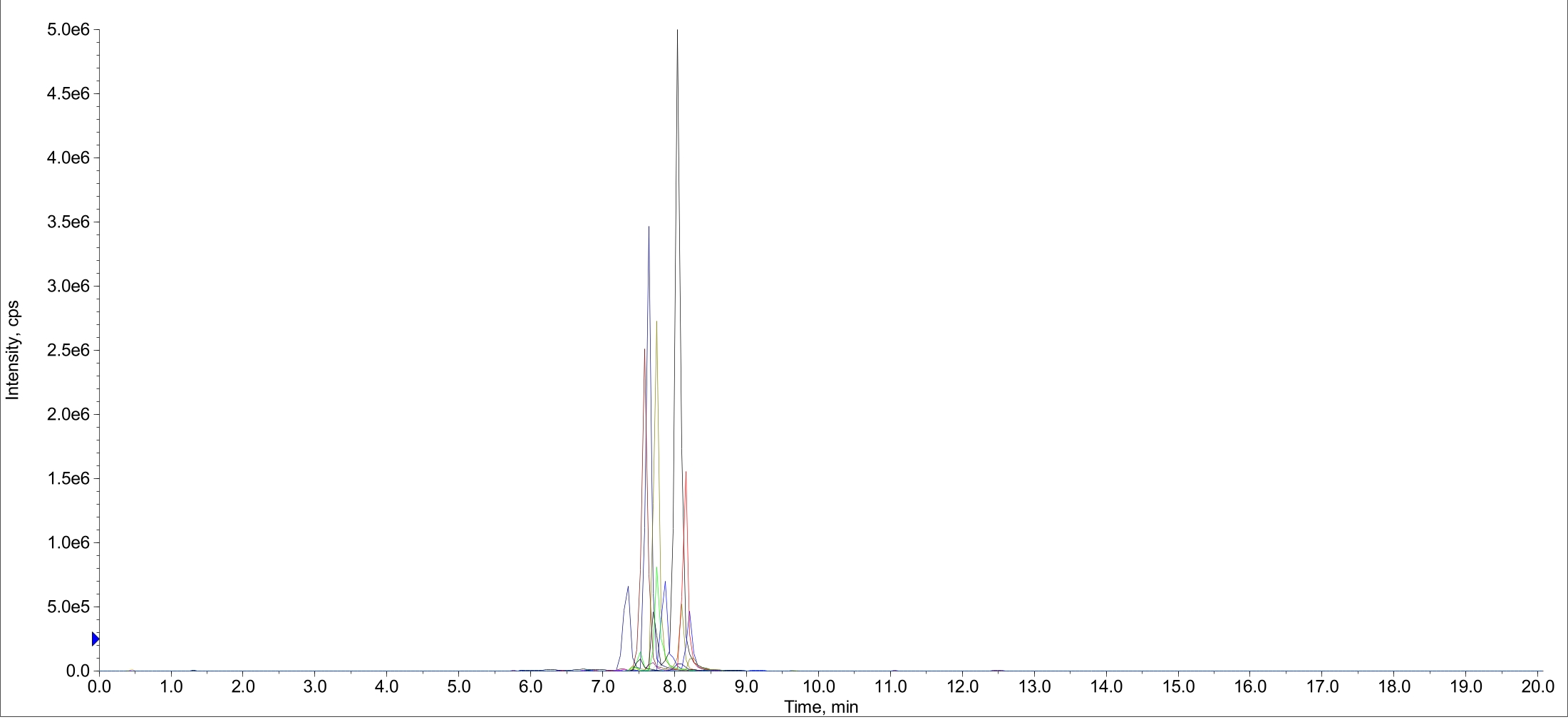


b1. PC b2. LPC b3. PE


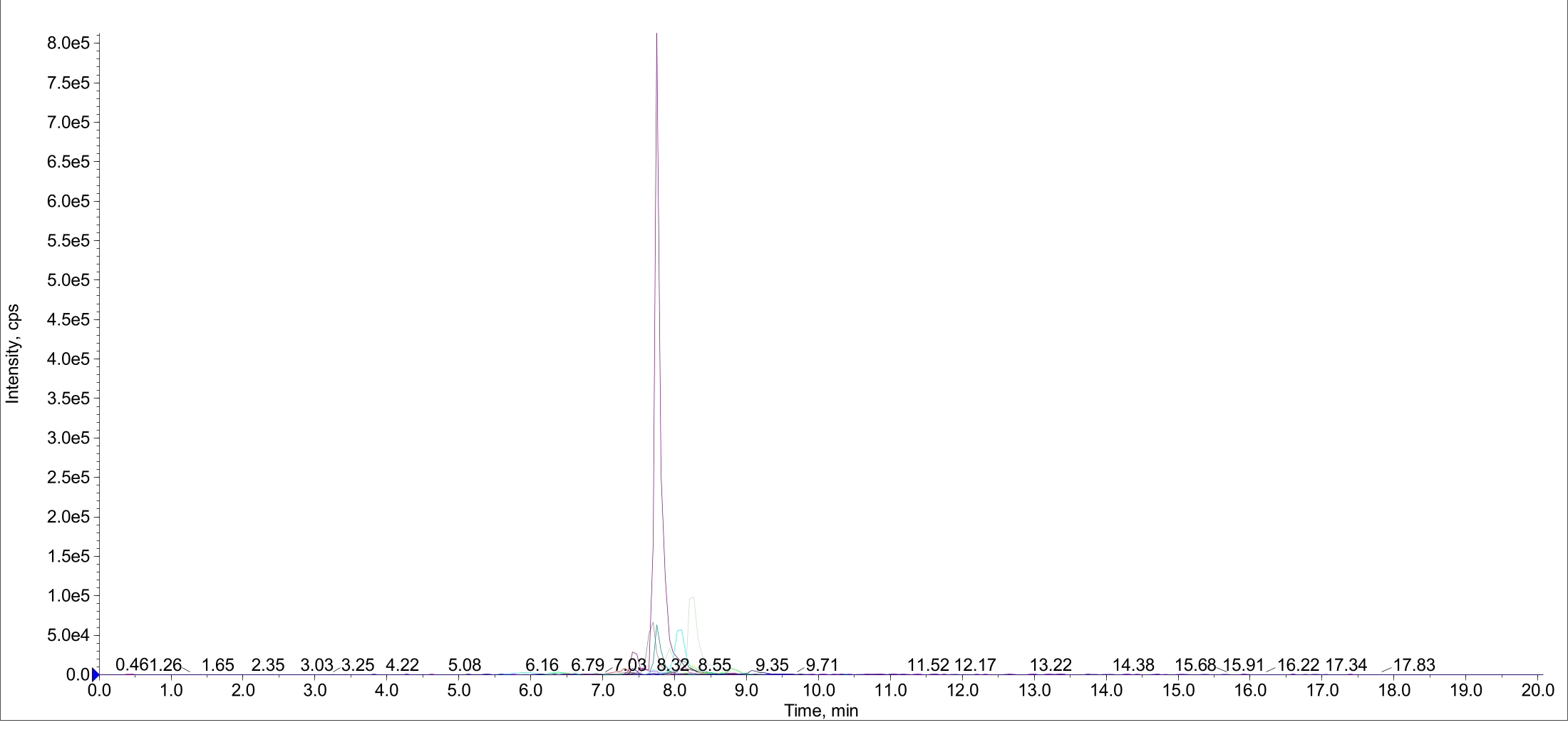

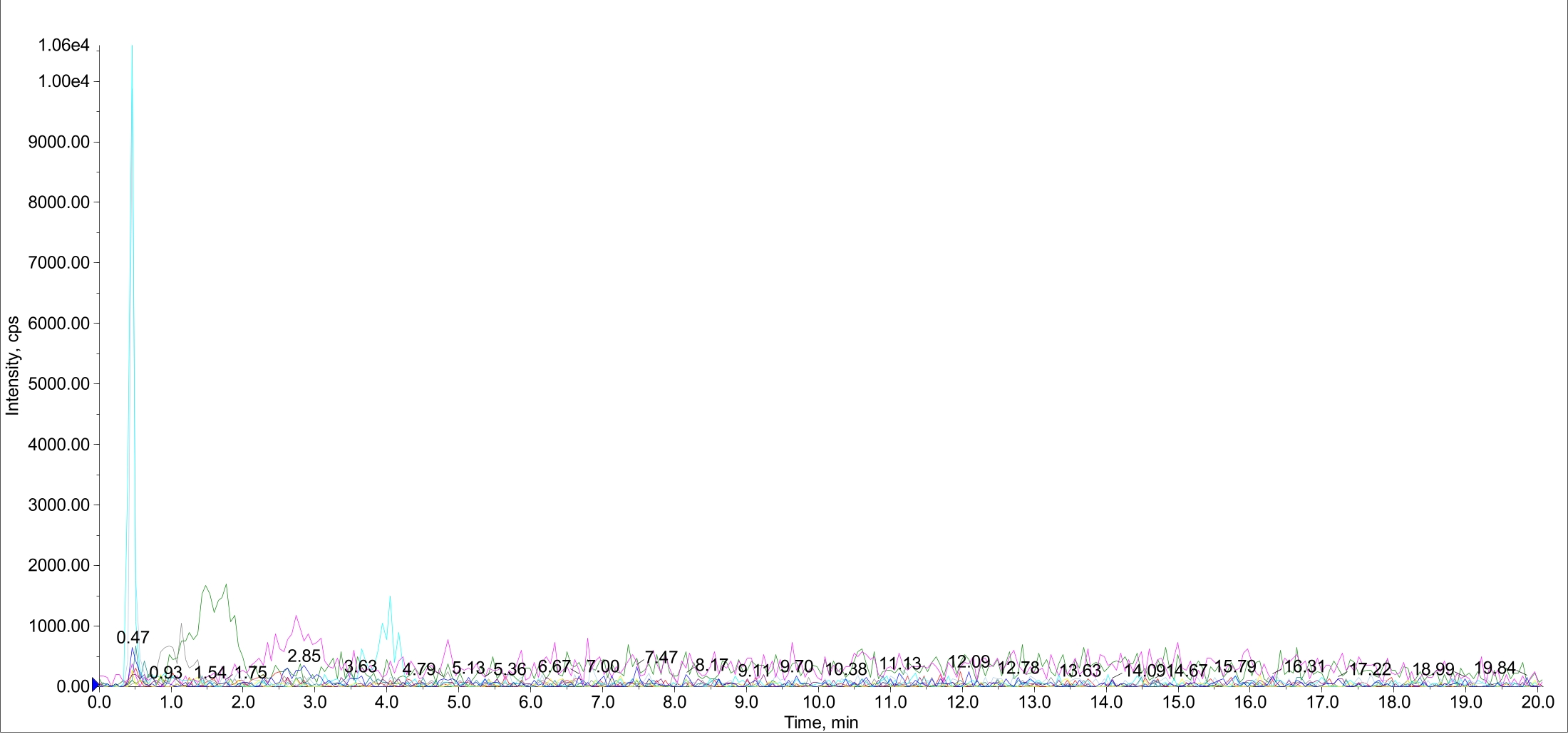

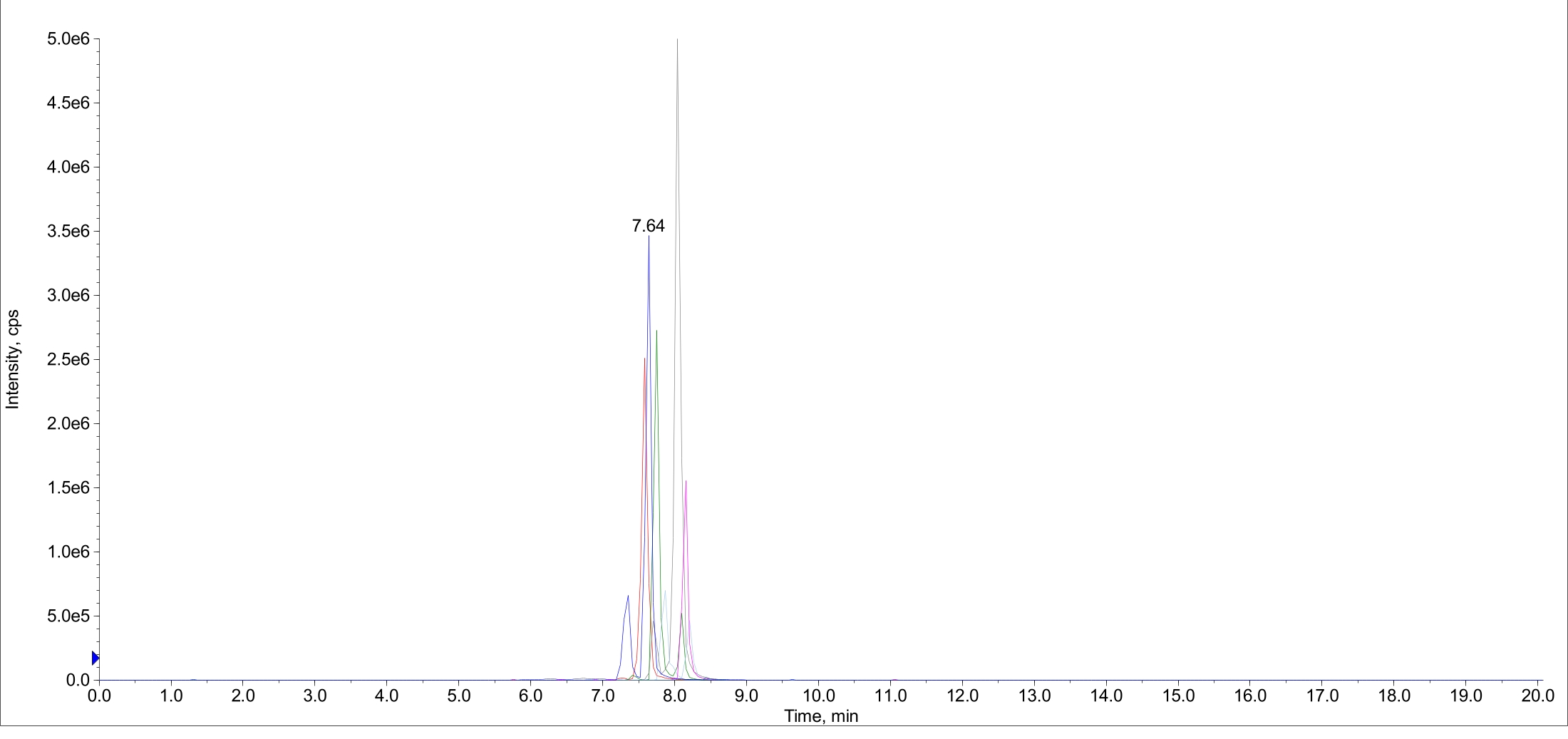


b4. PI b5. PS


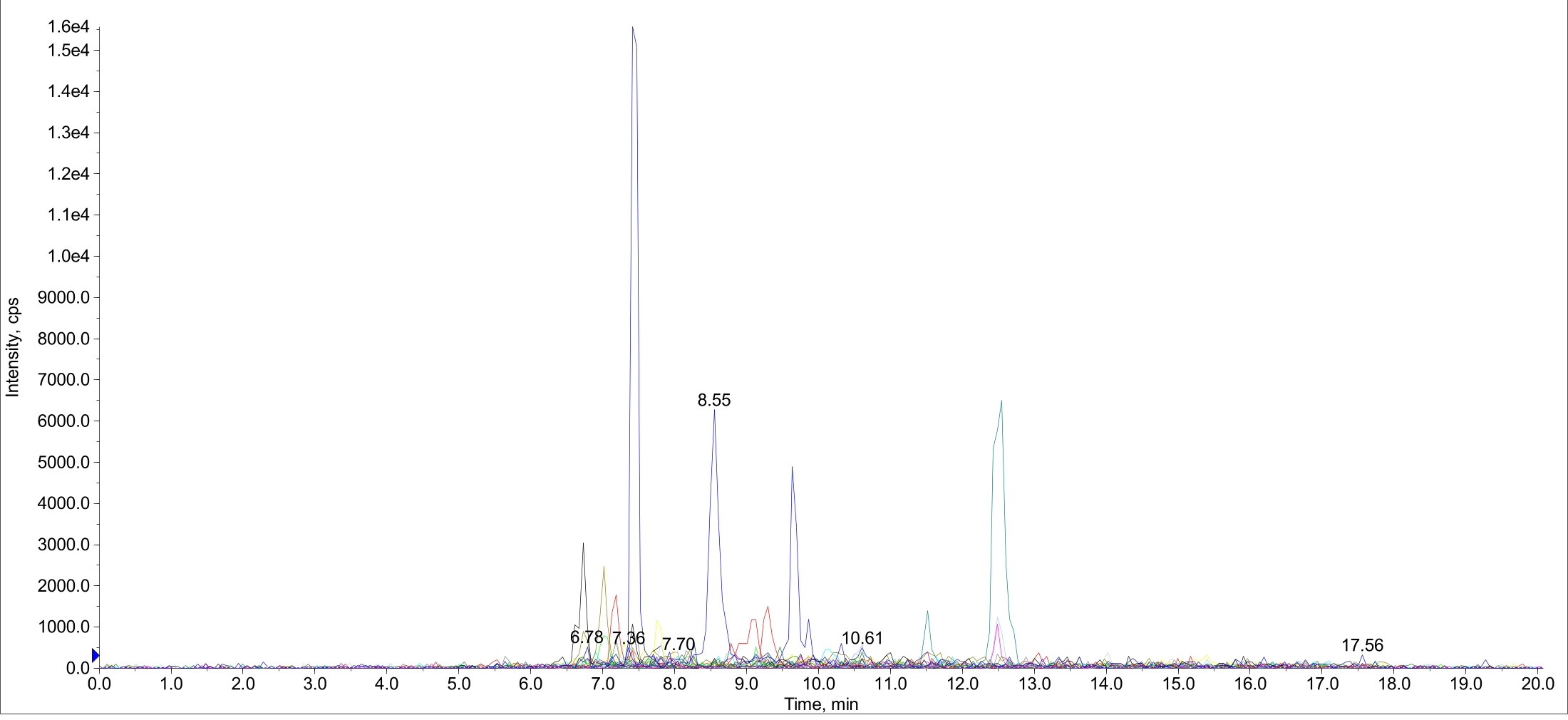

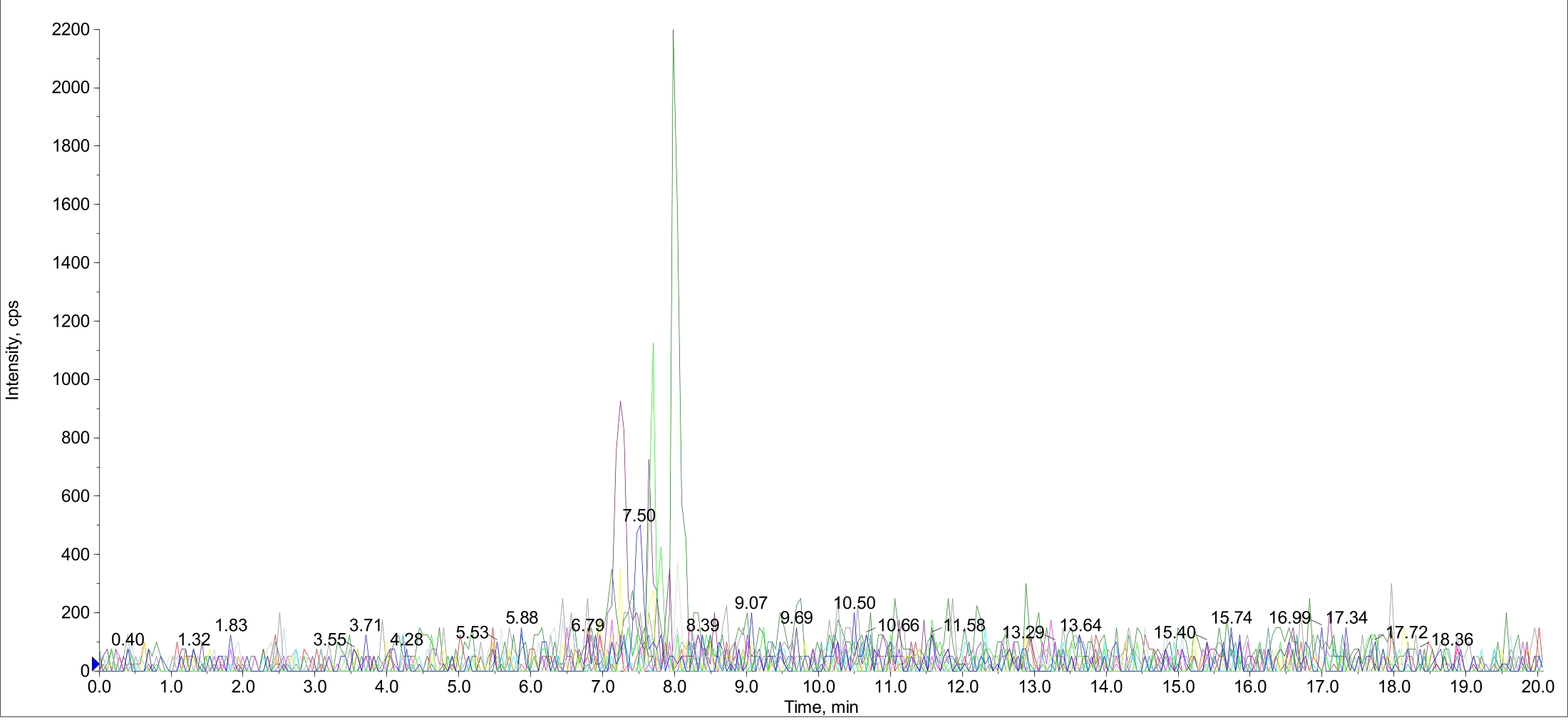


Fig. S3: Distribution percentages of quantified lipid species of TG, DG, PC, LPC, PE, PI, and PS.

1. **TL extract**


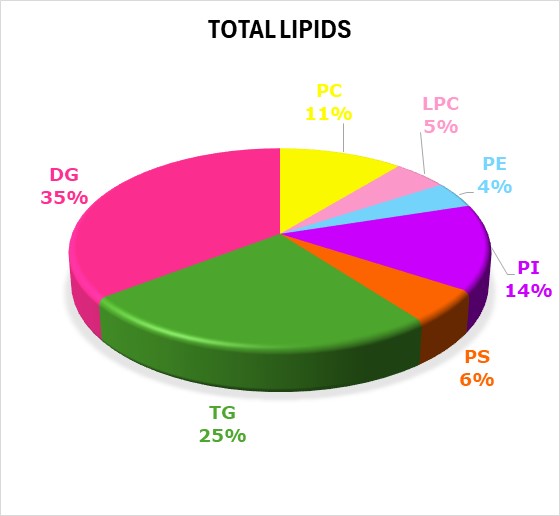


1. **CWL extract**


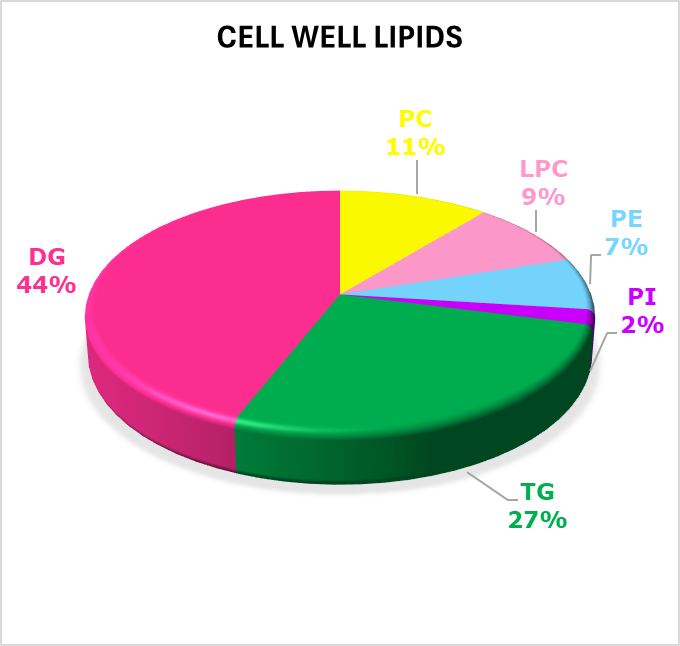


Fig.S4: Relative distribution of quantified lipid species of TG, DG, PC, LPC, PE, PI, and PS in percentage among MTB and *M. kansasii* isolates.

1. **TL extract**

1. **CWL extract**
